# Allostreptopyrroles A–E, β-alkylpyrrole derivatives from an actinomycete *Allostreptomyces* sp. RD068384

**DOI:** 10.3762/bjoc.20.174

**Published:** 2024-08-13

**Authors:** Marwa Elsbaey, Naoya Oku, Mohamed S A Abdel-Mottaleb, Yasuhiro Igarashi

**Affiliations:** 1 Pharmacognosy Department, Faculty of Pharmacy, Mansoura University, Mansoura 35516, Egypt; 2 Biotechnology Research Center and Department of Biotechnology, Toyama Prefectural University, 5180 Kurokawa, Imizu, Toyama 939-0398, Japan; 3 Computational Chemistry Lab, Department of Chemistry, Faculty of Science, Ain Shams University, 11566 Abbassia, Cairo, Egypt

**Keywords:** *Allostreptomyces*, β-alkylpyrrole, conformer, cytotoxic, DFT, 4-formylpyrrole-2-carboxylic acid

## Abstract

Five new β-alkylpyrrole derivatives, allostreptopyrroles A–E (**1**–**5**), were isolated from the culture broth of *Allostreptomyces* RD068384. Their structures were elucidated by 1D and 2D NMR spectroscopic analyses, HRESIMS, and chemical derivatization. The absolute configurations of compounds **2** and **3** were predicted by comparison of experimental and calculated specific rotation data. Compounds **1**–**5** are the first examples of natural pyrroles substituted by formyl and carboxyl functionalities. Compounds **1**, **4**, and **5** showed cytotoxicity against Kasumi-1 human acute myeloblastic leukemia cells with IC_50_ values of 103, 105, and 105 μM, respectively, which are less active than the anticancer agent cisplatin, with an IC_50_ value of 70 μM.

## Introduction

β-Alkylpyrroles are key structural motifs in biomolecules and functional organic materials [1]. For instance, β-alkylpyrroles are the main building blocks for the life-essential tetrapyrrole pigments (porphyrins) including heme, chlorophyll, and vitamin B12 [1–2] (Figure S54 in Supporting Information File 1). Porphobilinogen, the fundamental biological precursor of tetrapyrroles, is biosynthesized via asymmetric condensation of two δ-aminolevulinic acid molecules [2–3]. From another aspect, copolymerized β-alkylpyrroles are among the most investigated organic materials for their enhanced physical and electrochemical properties [4–5]. Accordingly, chemists have focused on developing selective synthetic strategies for the construction of β-alkylpyrroles [1].

**Figure 1:**
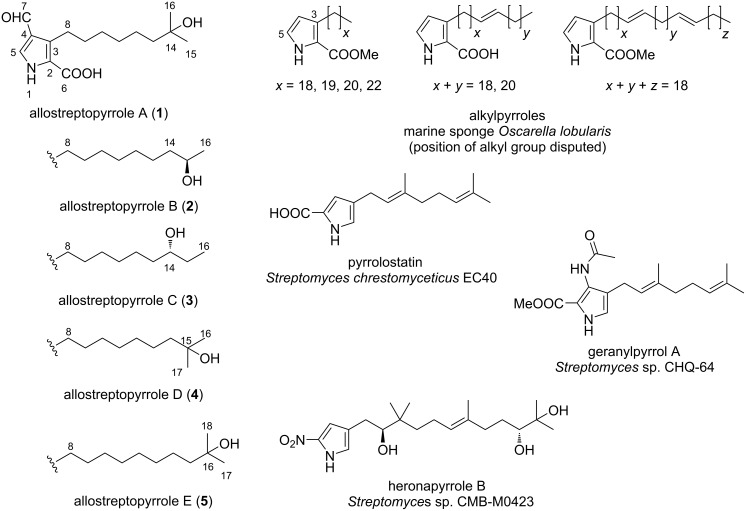
Structures of allostreptopyrroles A–E (**1**–**5**) and related metabolites.

While the pyrrole nucleus is featured in many marine natural products [6–7], pyrroles substituted with long hydrocarbon chains (pyrrole lipids) are seldomly isolated, and their presence is limited to certain marine organisms [8]. A series of 3-alkylpyrrole-2-carbaldehydes/carboxylic acid/methylcarboxylate was reported from the marine sponge *Oscarella lobularis* (Figure 1 and Figure S54 in Supporting Information File 1) [7,9], but the actual position of the alkyl chains is very likely to be on the 5 position, as Stierle and Faulkner pointed in their study on a series of 5-alkylpyrrole-2-carbaldehydes from the sponge *Laxosuberites* sp. [10]. From 1997 to 2017, over fifty 5-alkylpyrrole-2-carbaldehydes and 5-alkyl-2-hydroxymethylpyrroles with diversely functionalized alkyl side chains have been isolated from sponges of the genus *Mycale* [7,11], but no additional 3-alkylpyrroles were reported so far.

β-Alkylpyrroles are rare as microbial metabolites, and most of them are pyrroloterpenes from *Streptomyces* (Figure 1 and Figure S54 in Supporting Information File 1). Examples include pyrrolostatin [12] and its congener geranylpyrrol A [13], bearing a carboxylic group at the C2 and a geranyl group at the C4 position of the pyrrole ring, and their 2-nitro congeners, nitropyrrolins [14] and heronapyrroles [15], bearing a farnesyl chain at the C4 position. Pyrroloterpenes are proposed to be of mixed biogenesis, elaborated from an aromatic pyrrole moiety and a terpenoid chain [15]. Prodigiosin, a major metabolite of *Serratia*, is another example of β-alkylpyrrole, bearing a pentyl chain on the pyrrolyldipyrromethene core [16]. Similarly, α-alkylpyrroles are limited to a handful examples including α-pyrrolosesquiterpenes [17–19], undecylprodigiosin [16] from *Streptomyces*, and fungus-derived pyrrol-2-ylpolyenes [20].

In 2017, *Allostreptomyces* was introduced as a new genus in the family *Streptomycetaceae* [21], and two species, *A. psammosilenae* [21] and *A. indica* [22], are currently known. Only two 22-membered macrolides were reported from this genus [23] until we recently isolated five polycyclic tetramate-class macrolactams from *Allostreptomyces* sp. RD068384, including a new congener, allostreptamide [24]. Further investigation of this strain led to the isolation of five new β-alkylpyrroles, designated allostreptopyrroles A–E (**1**–**5**) (Figure 1).

## Results and Discussion

The fermentation extract of strain RD068384, cultured in A-3M medium, was fractionated on a silica gel column eluting with CHCl_3_/MeOH mixtures. Allostreptamide was obtained from the eluate with CHCl_3_/MeOH 2:1 [24]. HPLC-DAD analysis of a less polar fraction, eluted with CHCl_3_/MeOH 10:1, detected several peaks with characteristic UV absorptions, which were purified by ODS flash chromatography followed by ODS HPLC to yield compounds **1**–**5**.

**Figure 2:**
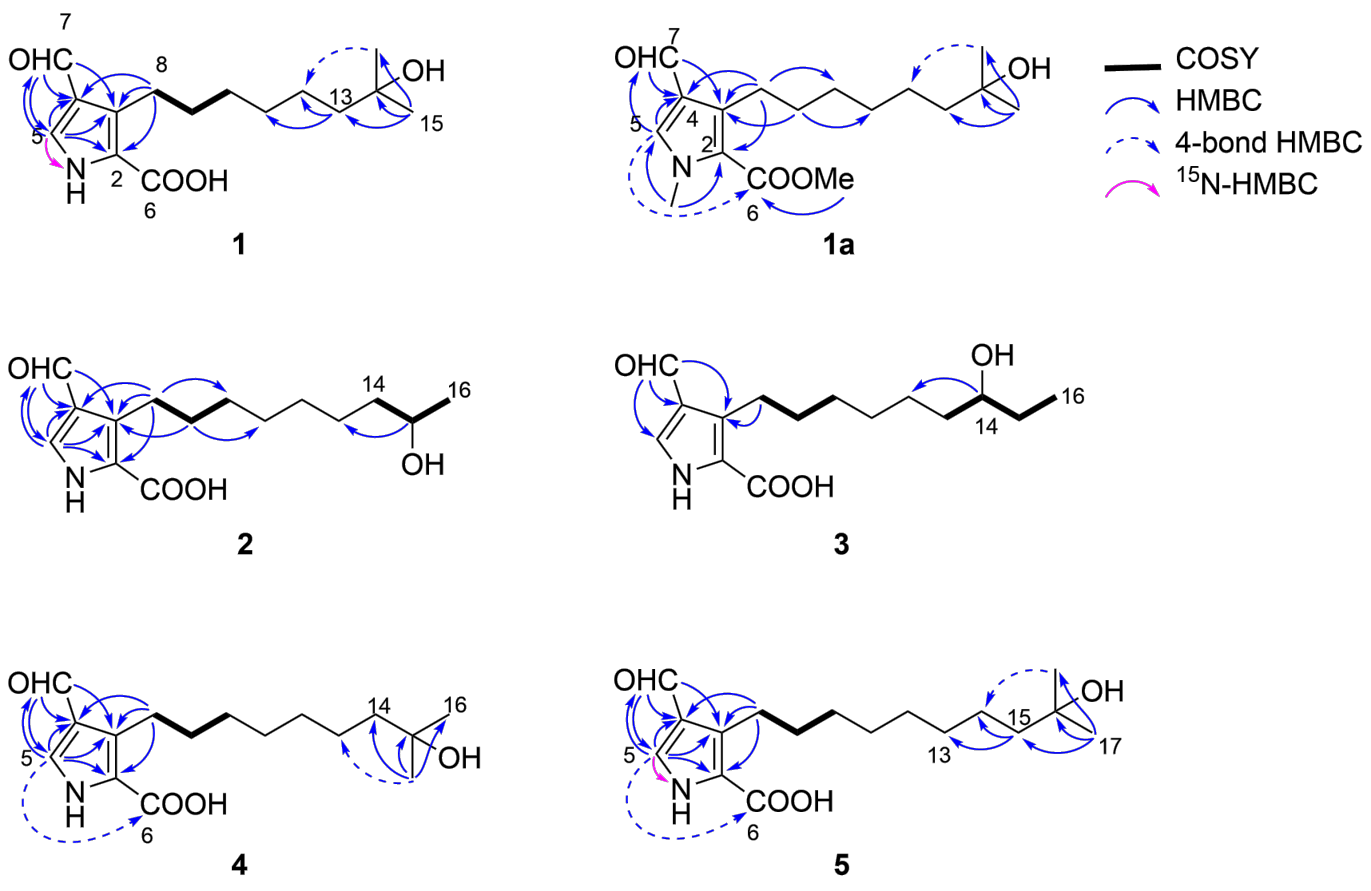
COSY, ^15^N-HMBC and key HMBC correlations of compounds **1**–**5** and **1a**.

Allostreptopyrrole A (**1**) was obtained as a greenish yellow amorphous solid. The molecular formula was determined to be C_15_H_23_NO_4_ based on a molecular ion peak at *m*/*z* 280.1550 [M − H]^−^ (calcd for 280.1554) observed in a negative HRESITOF mass spectrum. Analysis of ^1^H NMR, ^13^C NMR (Table 1), and HSQC spectra revealed a formyl group (δ_C_ 186.3/δ_H_ 9.89), an olefinic methine (δ_C_ 131.1/δ_H_ 7.64), an acyl carbonyl carbon (δ_C_ 163.3), three non-protonated olefinic carbons (δ_C_ 133.4, 126.4, and 123.1), a deshielded non-protonated sp^3^ carbon (δ_C_ 70.1), six sp^3^ methylenes (δ_C_ 25.0–44.8), and two magnetically equivalent tertiary methyl groups (δ_C_ 29.5/δ_H_ 1.13). These molecular parts accounted for four degrees of unsaturation out of five, leaving one degree for a ring structure. In addition, a highly conjugated functional group was suggested by UV maximal absorptions at 235 nm and 273 nm and HMBC correlations from the formyl and the olefinic methine protons to all sp^2^ carbons except the acyl carbonyl carbon (Figure 2 and Table S1 in Supporting Information File 1). The sp^3^ carbons, in contrast, constituted an alkyl chain: the six methylene units were connected in sequence to form a hexamethylene chain as supported by overlapping six proton resonances at δ_H_ 1.31–1.42 and by inter-unit COSY and HMBC correlations. This methylene chain was blocked by an oxypropyl group, as evident from HMBC correlations from the tertiary methyl protons (δ_H_ 1.13) to the oxygenated carbon (δ_C_ 70.1), and one of the methylene carbons (C13: δ_C_ 44.8).

**Table 1:** ^1^H and ^13^C NMR data for **1** and **1a**.^a^

	**1**		**1** ^b^		**1a**	
	
Position	δ_C_	δ_H_, mult, *J* in Hz	δ_C_	δ_H_, mult, *J* in Hz	δ_C_	δ_H_, mult, *J* in Hz

2	123.1	–	122.4	–	122.8	–
3	133.4	–	133.2	–	135.9	–
4	126.4	–	126.4	–	124.0	–
5	131.1	7.64, s	131.6	7.51, s	137.0	7.63, s
6	163.3	–	166.1^c^	–	162.5	–
7	186.3	9.89, s	188.4	9.77, s	185.6	9.83, s
8	25.4	3.13, t (7.5)	25.7	3.11, t (7.5)	26.0	3.06, t (7.8)
9	32.0	1.59, m	32.3	1.57, m	32.2	1.55, m
10	30.5^d^	1.39, m	30.6	1.38, m^e^	30.4^f^	1.28–1.44, m
11	30.9^d^	1.31, m	31.3	1.28–1.45, m	30.9	1.28–1.44, m
12	25.0	1.33–1.42, m	25.4	1.31–1.44, m	25.0	1.35–1.42, m
13	44.8	1.42, m	44.9	1.44, m	44.9	1.42, m
14	70.1	–	71.5	–	70.1	–
15	29.5^f^	1.13, s	29.1	1.15, s	29.6^f^	1.13, s
16	29.5^f^	1.13, s	29.1	1.15, s	29.6^f^	1.13, s
*N*CH_3_	–	–	161.4^g^	–	38.5	3.94, s
COOCH_3_	–	–	–	–	51.4	3.85, s

^a^NMR data were recorded in CD_3_COCD_3_ at 500 and 125 MHz for ^1^H and ^13^C, respectively. ^b^Recorded in CD_3_OD. ^c^Assigned from HMBC. ^d^Interchangeable. ^e^Assigned from COSY. ^f^Overlapping signals read from HSQC. ^g15^N chemical shift determined from ^15^N HMBC.

The formyl proton H7 showed HMBC correlations to the olefinic carbons C3, C4, and C5 and the olefinic methine proton H5 was correlated with C2, C3, C4, and C7. These correlation data allowed the assignment of a carbon sequence C2–C3–C4–C5 and the attachment of the formyl group at C4. Furthermore, HMBC correlations from two methylene protons H_2_8 to the olefinic carbons C2, C3, and C4 connected the chain part at C3. A ^1^H,^15^N-HMBC correlation was seen from H5 to a nitrogen at δ_N_ 161.4, which suggested the presence of a nitrogen atom adjacent to C5. A correlation to the acyl carbonyl carbon (C6) was not available at this stage. In order to obtain further information for connectivity, compound **1** was reacted with methyl iodide and K_2_CO_3_ to give a bismethylated derivative **1a**. A methyl proton at δ_H_ 3.94 was of an *N*-methyl group (δ_C_ 38.5) and displayed two strong HMBC correlations to C2 and C5, which connected these carbons through a nitrogen atom to establish a pyrrole ring, and also a hydroxy group at the alkyl terminus. Another methyl proton at δ_H_ 3.85 was of a methoxy group (δ_C_ 51.4) and had only one HMBC correlation to C6, which provided a methoxycarbonyl (–COOMe) fragment. Finally, this fragment was placed at C2 by an HMBC correlation from H5 to C6 to complete the gross structure of **1**.

Both compounds **2** and **3** were obtained as greenish yellow amorphous and their molecular formula were suggested to be the same as that of **1** from HRESITOFMS and NMR analytical data (Table 2), inferring that compounds **2** and **3** were isomers of **1**. In fact, their NMR spectra were closely similar to those for **1** except a little difference in the alkyl side chain terminus. In a COSY spectrum of **2**, the terminal doublet methyl proton was correlated with an oxymethine H15, which in turn was correlated with a methylene H_2_14. The pyrrole moiety with the same substituents as **1** was deduced from HMBC correlations. Therefore, compound **2** was determined to have a non-branched alkyl chain with a hydroxy group at C15. Meanwhile, **3** possessed a terminal ethyl group, which was connected to an oxymethine H14 in a COSY spectrum, thereby establishing a non-branched alkyl chain with a hydroxy group at C14.

**Table 2:** ^1^H and ^13^C NMR data for compounds **2** and **3** in CD_3_COCD_3_.

	**2**		**3**	
	
Position	δ_C_	δ_H,_ mult, *J* in Hz	δ_C_	δ_H,_ mult, *J* in Hz

2	123.0	–	122.8	–
3	133.5	–	133.7	–
4	126.5	–	126.5	–
5	131.2	7.64, s	131.3	7.66, s
6	163.0	–	162.9	–
7	186.3	9.89, s	186.3	9.90, s
8	25.3	3.13, t (7.0)	25.4	3.14, brs
9	32.0	1.59, m	32.0	1.59, m
10	30.6^a^	1.38, m^b^	30.6^c^	1.39, m^b^
11	30.4^a^	1.25–1.46, m	31.1^a^	1.26–1.48, m
12	30.5^a^	1.25–1.46, m	26.5	1.32–1.44, m
13	26.6	1.31–1.41, m	38.0	1.37, m
14	40.3	1.36–1.40, m	72.7	3.42, br
15	67.5	3.68, m	31.0^a^	1.36, 1.46, m^b^
16	24.0	1.10, d (6.1)	10.5	0.90, t (7.1)

^a^Interchangeable. ^b^Assigned from COSY. ^c^Overlapping signals read from HSQC.

The specific rotation values of **2** and **3** were calculated to predict their absolute configurations. For the flexible molecules **2** and **3**, thousands of conformers may exist (over 52400 conformers). However, only a few are usually significantly populated (i.e., the compound exists as a rapidly equilibrating mixture of multiple conformers). In this situation, the spectroscopic properties of a molecule can be calculated as the average over the conformers, weighted according to their populations [25]. The calculated specific rotations −11.4 and +16.1 were obtained for *R*-configured **2** and **3** from the DFT computations (see DFT methodology section), respectively, which were in good agreement with the experimentally obtained values, −6.1 for **2** and +15 for **3**. Thus, *R*-configurations were proposed for compounds **2** and **3**. However, this prediction was not confirmed by chemical derivatization due to their limited availability.

^1^H and ^13^C NMR spectra of compounds **4** and **5** were superimposable to those of **1** except for methylene resonances, supporting that both **4** and **5** possess the same substituted pyrrole ring and hydroxyisopropyl terminus as compound **1** (Table 3). HRESITOFMS analysis determined the molecular formula of **4** to be C_16_H_25_NO_4_ and that of **5** to be C_17_H_27_NO_4_, which established that **4** and **5** are one- and two-methylene-longer congeners of **1**.

**Table 3:** ^1^H and ^13^C NMR data for **4** and **5**.

	**4** in CD_3_COCD_3_		**5** in CD_3_OD	
	
Position	δ_C_	δ_H,_ mult, *J* in Hz	δ_C_	δ_H,_ mult, *J* in Hz

2	122.4	–	125.2	–
3	133.8	–	133.6	–
4	126.5	–	126.4	–
5	131.4	7.68, s	131.8	7.54, s
6	162.5	–	165.8	–
7	186.3	9.90, s	188.4	9.78, s
8	25.3	3.13, t (7.8)	25.7	3.10, t (7.3)
9	32.0	1.60, m	32.4	1.56, m
10	30.2^a^	1.38, m^b^	30.6^c^	1.39, m^b^
11	31.1^c^	1.26–1.44, m	30.7^c^	1.27–1.46, m
12	31.0^c^	1.26–1.44, m	30.6^c^	1.27–1.46, m
13	25.0	1.32–1.41, m	31.4	1.27–1.46, m
14	44.8	1.42, m	25.4	1.31–1.41, m
15	70.1	–	44.9	1.44, m
16	29.5^a^	1.14, s	71.5	–
17	29.5^a^	1.14, s	29.1	1.16, s
18	–	–	29.1	1.16, s
*N*	–	–	162.3^d^	–

^a^Overlapping signals read from HSQC. ^b^Assigned from COSY. ^c^Interchangeable. ^d15^N chemical shift determined from ^15^N HMBC.

Compounds **1**, **4**, and **5** showed moderate cytotoxicity against Kasumi-1 human acute myeloblastic leukemia cells with IC_50_ values of 103, 105, and 105 while **2** and **3** were less active with IC_50_ values of 200 and 333 μM, respectively. Under the same experimental conditions, cisplatin, a positive control, inhibited the cell growth with an IC_50_ value of 70 μM. Compounds **1**–**5** were merely inhibitory against tyrosinase, showing 19, 13, 9.6, 18, and 15% inhibition at 200 μM, respectively, while a positive control, kojic acid, inhibited the same enzyme by 95%.

## Conclusion

In summary, five new alkylpyrroles, allostreptopyrroles A–E (**1**–**5**), were discovered from a fermentation extract of *Allostreptomyces* sp. RD068384, a strain belonging to an almost unstudied actinomycetes genus within the family *Streptomycetaceae*.

Compounds **1**–**5** are characterized by a pyrrole-2-carboxylic acid core decorated with a formyl group and an alkyl side chain. Secondary metabolites of this specific composition have not been reported. The pyrrole-2-carboxyl skeleton is a recurring framework in pyrrolic natural products including microbial pyrrolostatin and aminocoumarin antibiotics [2], plant-derived brachystemidines [26], and lamellarins from marine invertebrates [6] (Figure S55 in Supporting Information File 1). Biosynthetically, pyrrole-2-carboxylic acid is known to be derived from ʟ-proline [2]. Similarly, pyrrole-2-carbaldehydes have been isolated from various natural sources including plants, marine invertebrates, and fungi [7], while **1**–**5** are the first to have formyl and carboxyl functionalities. Furthermore, a β-alkyl substitution is not very common in pyrrolic secondary metabolites. The most related metabolites to **1**–**5** are the reported alkylpyrroles from a marine sponge *Oscarella lobularis* [7] and pyrroloterpenes from *Streptomyces* [12–15], although the substitution patterns are different (Figure 1). Natural alkylpyrroles were shown to have cytotoxicity [27], antidiabetic activity [28], anti-lipid peroxidation [12], in vivo antihypoxic activity [12], and antibacterial activity [15]. Though not impressive in cytotoxicity and tyrosinase-inhibitory evaluations, compounds **1**–**5** could be more potent in some other bioassays, which is a subject of future studies. Finally, these results supported that actinomycetes genera with little or no chemical study are a fruitful reservoir for discovering new natural molecules.

## Experimental

### Microorganism, fermentation, extraction, and isolation

Details on the supplier of *Allostreptomyces* sp. RD068384, fermentation, extraction, and fractionation are described in Supporting Information File 1. While a CHCl_3_/MeOH 2:1-eluting fraction by silica gel open column chromatography eventually yielded allostreptamide [24], a less polar CHCl_3_/MeOH 10:1 fraction contained compounds with characteristic UV absorption. This fraction, obtained as 395 mg of brown solid from 4 L culture in A-3M medium, was fractionated by octadecyldimethylsilyl (ODS) silica gel column chromatography with a gradient of MeCN/0.1% HCO_2_H solution (2:8, 3:7, 4:6, 5:5, 6:4, 7:3, and 8:2, v/v). The third (4:6) and fourth fractions (5:5) contained the peaks of our target, which were combined (35 mg) and purified by ODS-HPLC (Cosmosil C18 AR-II, 10 × 250 mm, 4 mL/min, UV detection at 254 nm) eluted with 33% MeCN/0.1% HCO_2_H solution to yield **1** (1.2 mg, *t*_R_ 10.1 min), **2** (0.9 mg, *t*_R_ 12.1 min), **3** (0.8 mg, *t*_R_ 13.7 min), and **4** (2.3 mg, *t*_R_ 17.3 min). At time 22 min, the MeCN concentration was raised to 35%, which eluted **5** (1.7 mg) at *t*_R_ 28.9 min. To obtain higher amounts of compounds, fermentation with 4 L culture and isolation were repeated twice to afford in total 6.5 mg of **1**, 3.1 mg of **2**, 2.6 mg of **3**, 7.2 mg of **4**, and 5.6 mg of **5** from 12 L culture.

Allostreptopyrrole A (**1**): greenish yellow amorphous solid; UV (MeOH) λ_max_ nm (log ε) 234 (3.86), 273 sh (3.44); IR (ATR) ν_max_: 3275, 2964, 2928, 2855, 1658, 1554, 1418 cm^−1^; ^1^H and ^13^C NMR data, see Table 1; HRESITOFMS (*m*/*z*): [M – H]^–^ calcd for C_15_H_22_NO_4_, 280.1554; found, 280.1550.

Allostreptopyrrole B (**2**): greenish yellow amorphous solid; 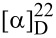 +15 (*c* 0.10, MeOH); UV (MeOH) λ_max_, nm (log ε): 235 (3.87), 273 sh (3.49); IR (ATR) ν_max_: 3263, 2964, 2925, 2854, 1658, 1556, 1417 cm^−1^; ^1^H and ^13^C NMR data, see Table 2; HRESITOFMS (*m*/*z*): [M – H]^–^ calcd for C_15_H_22_NO_4_, 280.1554; found, 280.1554.

Allostreptopyrrole C (**3**): greenish yellow amorphous solid; 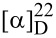 −6.1 (*c* 0.10, MeOH); UV (MeOH) λ_max_, nm (log ε): 235 (3.82), 276 sh (3.46); IR (ATR) ν_max_: 3265, 2925, 2856, 1657, 1555, 1417 cm^−1^; ^1^H and ^13^C NMR data, see Table 2; HRESITOFMS (*m*/*z*): [M – H]^–^ calcd for C_15_H_22_NO_4_, 280.1554; found, 280.1556.

Allostreptopyrrole D (**4**): greenish yellow amorphous solid; UV (MeOH) λ_max_, nm (log ε): 234 (3.87), 273 sh (3.49); IR ν_max_: 3263, 2966, 2926, 2854, 1659, 1557, 1417 cm^−1^; ^1^H and ^13^C NMR data, see Table 3; HRESITOFMS (*m*/*z*): [M – H]^–^ calcd for C_16_H_24_NO_4_, 294.1711; found, 294.1704.

Allostreptopyrrole E (**5**): greenish yellow amorphous solid; UV (MeOH) λ_max_, nm (log ε): 235 (3.90), 273 sh (3.49); IR ν_max_: 3270, 2964, 2927, 2858, 1659, 1555, 1416 cm^−1^; ^1^H and ^13^C NMR data, see Table 3; HRESITOFMS (*m*/*z*): [M + Na]^+^ calcd for C_17_H_27_NO_4_Na, 332.1832; found, 332.1838.

### Methylation of **1**

Allostreptopyrrole A (**1**, 2.0 mg, 0.007 mmol) and K_2_CO_3_ (4.4 mg, 0.032 mmol) were stirred in dry DMF (0.5 mL) at 50 °C for 10 min. Methyl iodide (19 μL, 0.32 mmol) was added and the mixture was stirred at this temperature for 12 h [29]. Reaction completion was monitored by TLC. The solution was diluted with water and extracted with EtOAc three times. The organic layer was washed with brine and evaporated to dryness to afford bismethylated derivative of **1** (**1a**, 1.9 mg, 88% yield).

### DFT methodology

Prior to the calculations of the molecular properties of compounds **2** and **3**, their conformational ensembles, datasets of a structure and population of each conformer [25], were determined using a Spartan 24 parallel package conformational search tool (Wavefunction Inc, USA), followed by geometry optimization of the most weighted conformers (Boltzmann distribution weight lower to 0.004). Eleven and seven weighted conformers for compounds **2** and **3** were obtained, respectively, each forming equilibrium mixtures. The quantum chemical method for the calculation of conformer distribution was ωB97X-V/6-311+G(2df,2p)[6-311G*] [30]. The CPCM solvation model for methanol was used [31]. Range-separated hybrid GGA (RSH-GGA) functional, including dispersive interaction with 6-31G* as the polarization basis set (ωB97X-D/6-31G* method), was used for energy and geometry optimization [32].

### Bioactivity

Cytotoxicity and tyrosinase assays were carried out according to the procedures previously described [33–34]. The detailed procedures are available in Supporting Information File 1.

## Supporting Information

File 1 1D and 2D NMR, MS, UV, and IR spectra of compounds **1**–**5**; experimental section including general experimental procedures, microorganism, detailed procedures for fermentation, extraction, isolation, and bioassays.

## Data Availability

All data that supports the findings of this study is available in the published article and/or the supporting information to this article.
