# Supplementary material for: Allostreptopyrroles A–E, β-alkylpyrrole derivatives from an actinomycete Allostreptomyces sp. RD068384
Source: Beilstein J Org Chem. 2024 Aug 13;20:1981–7. doi: 10.3762/bjoc.20.174 (PMC11331545; doi:10.3762/bjoc.20.174)
Supplement: File 1 — 1D and 2D NMR, MS, UV, and IR spectra of compounds 1–5; experimental section including general experimental procedures, microorganism, detailed procedures for fermentation, extraction, isolation, and bioassays. [file Beilstein_J_Org_Chem-20-1981-s001.pdf]

BEILSTEIN JOURNAL OF ORGANIC CHEMISTRY

Supporting Information

for

Allostreptopyrroles A–E, β-alkylpyrrole derivatives from an actinomycete *Allostreptomyces* sp. RD068384

Marwa Elsbaey, Naoya Oku, Mohamed S. A. Abdel-Mottaleb and Yasuhiro Igarashi

Beilstein J. Org. Chem. **2024,** *20,* 1981–1987. doi:10.3762/bjoc.20.174

1D and 2D NMR, MS, UV, and IR spectra of compounds 1–5; experimental section including general experimental procedures, microorganism, detailed procedures for fermentation, extraction, isolation, and bioassays

License and Terms: This is a supporting information file under the terms of the Creative Commons Attribution License (https://creativecommons.org/licenses/by/4.0). Please note that the reuse, redistribution and reproduction in particular requires that the author(s) and source are credited and that individual graphics may be subject to special legal provisions.
The license is subject to the *Beilstein Journal of Organic Chemistry* terms and conditions: (https://www.beilstein-journals.org/bjoc/terms)

Table of contents

**Figure S1.** ^{1}H NMR spectrum of **1** (500 MHz, CD_3COCD_3)
**Figure S2.** ^{13}C NMR spectrum of **1** (125 MHz, CD_3COCD_3)
**Figure S3.** COSY spectrum of **1** (500 MHz, CD_3COCD_3)
**Figure S4.** HSQC spectrum of **1** (500 MHz, CD_3COCD_3)
**Figure S5.** HMBC spectrum of **1** (500 MHz, CD_3COCD_3)
**Figure S6.** Negative HRESITOF mass spectrum of **1**
**Figure S7.** IR spectrum of **1**
**Figure S8.** UV spectrum of **1**
**Figure S9.** ^{1}H NMR spectrum of **1** (500 MHz, CD_3OD)
**Figure S10.** ^{13}C NMR spectrum of **1** (125 MHz, CD_3OD)
**Figure S11.** COSY spectrum of **1** (500 MHz, CD_3OD)
**Figure S12.** HSQC spectrum of **1** (500 MHz, CD_3OD)
**Figure S13.** HMBC spectrum of **1** (500 MHz, CD_3OD)
**Figure S14.** ^{15}N-HMBC spectrum of **1** (500 MHz, CD_3OD)
**Figure S15.** ^{1}H NMR spectrum of **1a** (500 MHz, CD_3COCD_3)
**Figure S16.** ^{13}C NMR spectrum of **1a** (125 MHz, CD_3COCD_3)
**Figure S17.** HSQC spectrum of **1a** (500 MHz, CD_3COCD_3)
**Figure S18.** HMBC spectrum of **1a** (500 MHz, CD_3COCD_3)
**Figure S19.** Positive ESITOF mass spectrum of **1a**
**Figure S20.** ^{1}H NMR spectrum of **2** (500 MHz, CD_3COCD_3)
**Figure S21.** ^{13}C NMR spectrum of **2** (125 MHz, CD_3COCD_3)
**Figure S22.** COSY spectrum of **2** (500 MHz, CD_3COCD_3)
**Figure S23.** HSQC spectrum of **2** (500 MHz, CD_3COCD_3)
**Figure S24.** HMBC spectrum of **2** (500 MHz, CD_3COCD_3)
**Figure S25.** Negative HRESITOF mass spectrum of **2**
**Figure S26.** IR spectrum of **2**
**Figure S27.** UV spectrum of **2**
**Figure S28.** ^{1}H NMR spectrum of **3** (500 MHz, CD_3COCD_3)
**Figure S29.** ^{13}C NMR spectrum of **3** (125 MHz, CD_3COCD_3)
**Figure S30.** COSY spectrum of **3** (500 MHz, CD_3COCD_3)
**Figure S31.** HSQC spectrum of **3** (500 MHz, CD_3COCD_3)
**Figure S32.** HMBC spectrum of **3** (500 MHz, CD_3COCD_3)
**Figure S33.** Negative HRESITOF mass spectrum of **3**
**Figure S34.** IR spectrum of **3**
**Figure S35.** UV spectrum of **3**

Table of contents cont.

**Figure S36.** ^{1}H NMR spectrum of **4** (500 MHz, CD_3COCD_3)
**Figure S37.** ^{13}C NMR spectrum of **4** (125 MHz, CD_3COCD_3)
**Figure S38.** COSY spectrum of **4** (500 MHz, CD_3COCD_3)
**Figure S39.** HSQC spectrum of **4** (500 MHz, CD_3COCD_3)
**Figure S40.** HMBC spectrum of **4** (500 MHz, CD_3COCD_3)
**Figure S41.** Negative HRESITOF mass spectrum of **4**
**Figure S42.** IR spectrum of **4**
**Figure S43.** UV spectrum of **4**
**Figure S44.** ^{1}H NMR spectrum of **5** (500 MHz, CD_3OD)
**Figure S45.** ^{13}C NMR spectrum of **5** (125 MHz, CD_3OD)
**Figure S46.** COSY spectrum of **5** (500 MHz, CD_3OD)
**Figure S47.** HSQC spectrum of **5** (500 MHz, CD_3OD)
**Figure S48.** HMBC spectrum of **5** (500 MHz, CD_3OD)
**Figure S49.** ^{15}N-HMBC spectrum of **5** (500 MHz, CD_3OD)
**Figure S50.** Positive HRESITOF mass spectrum of **5**
**Figure S51.** Negative ESITOF mass spectrum of **5**
**Figure S52.** IR spectrum of **5**
**Figure S53.** UV spectrum of **5**
**Figure S54.** Representatives for natural products incorporating a *β*-alkyl-pyrrole scaffold
**Figure S55.** Representatives for secondary metabolites incorporating a pyrrole-2-carboxylic acid scaffold
**Table S1.** HMBC (^{1}H to ^{13}C) for compounds (**1–5** and **1a**) at 500 MHz
**Experimental**

Figure S1. ^{1}H NMR spectrum of **1** (500 MHz, CD_3COCD_3)

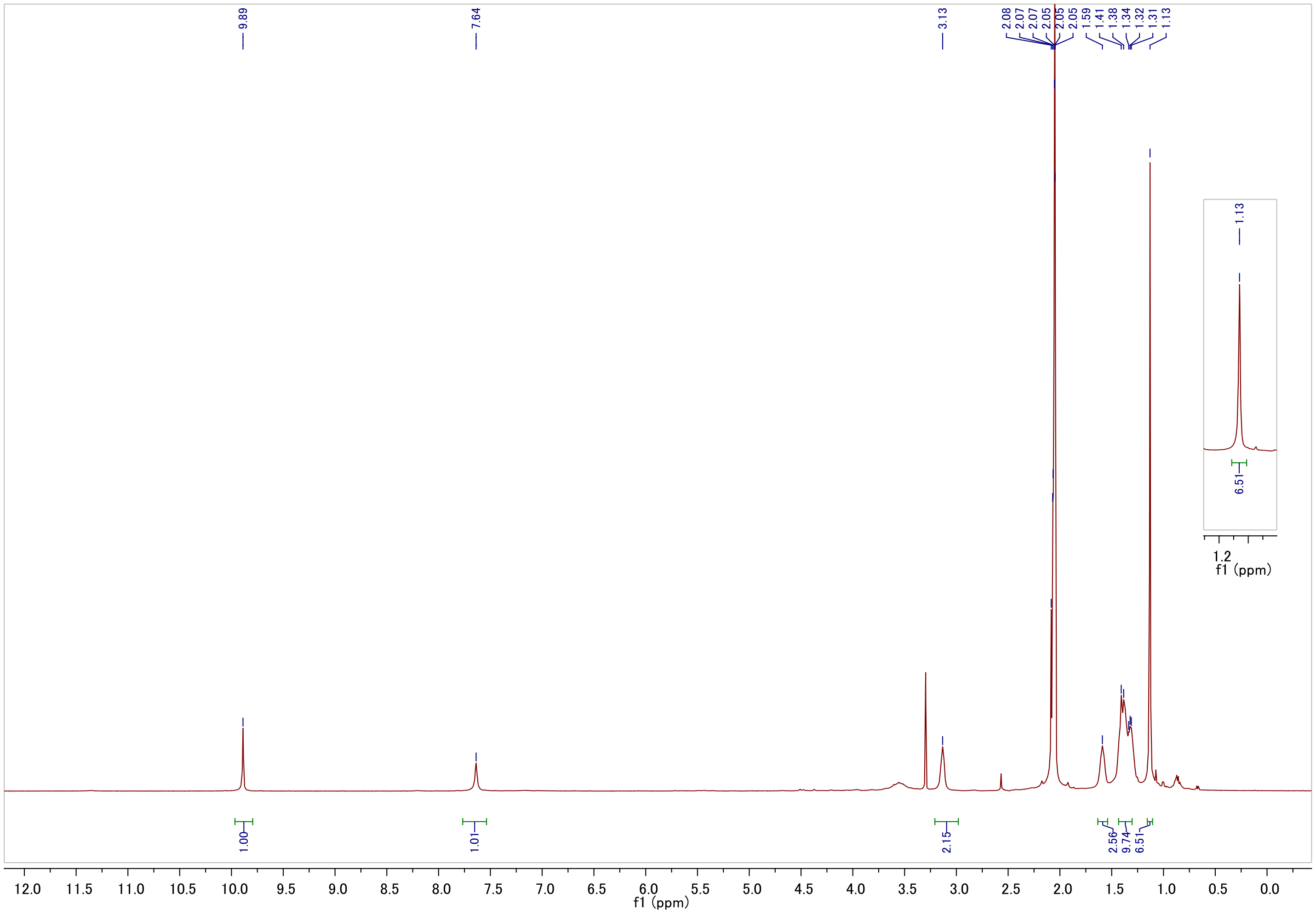

Figure S2. ^{13}C NMR spectrum of **1** (125 MHz, CD_3COCD_3)

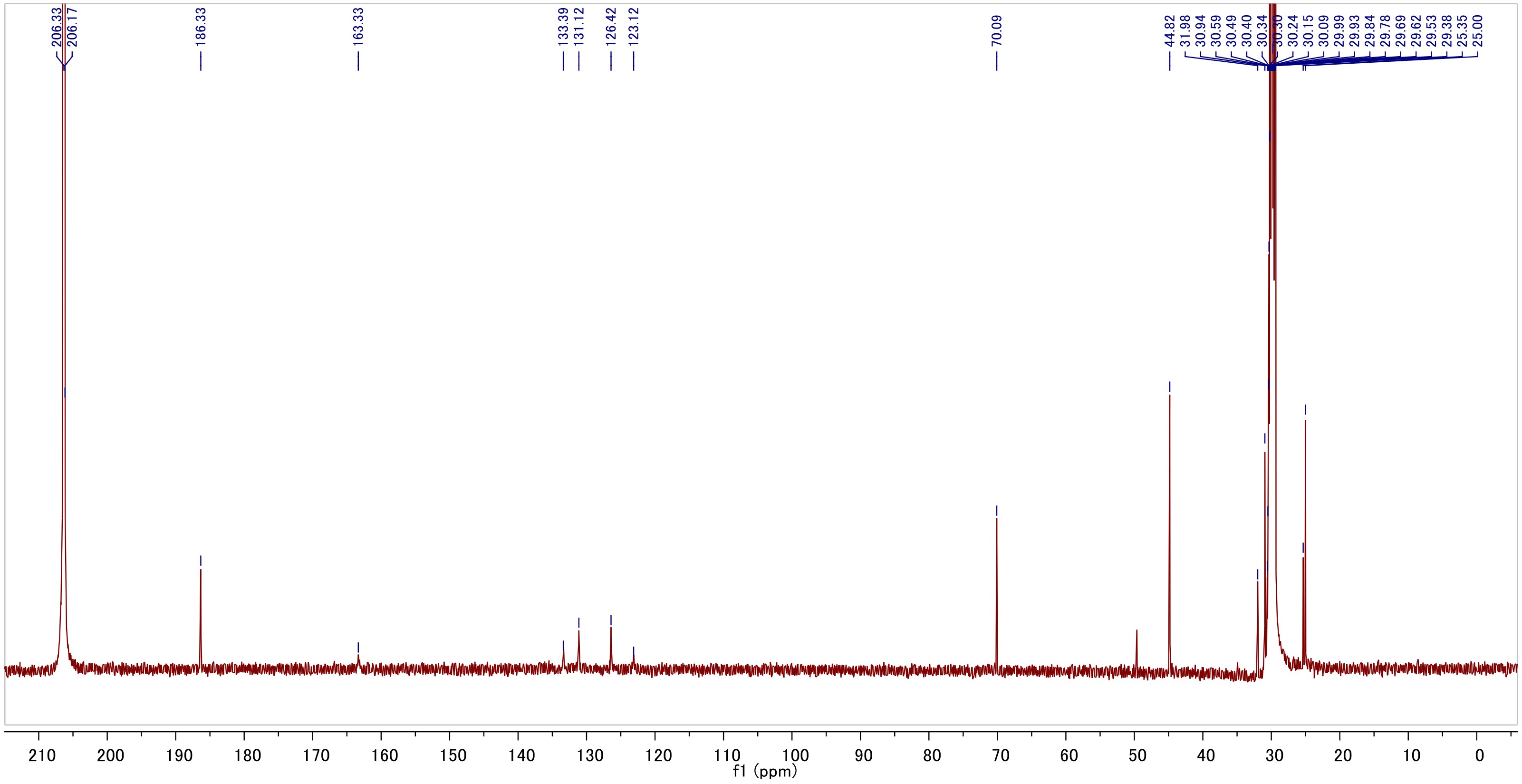

Figure S3. COSY spectrum of **1** (500 MHz, CD_3COCD_3)

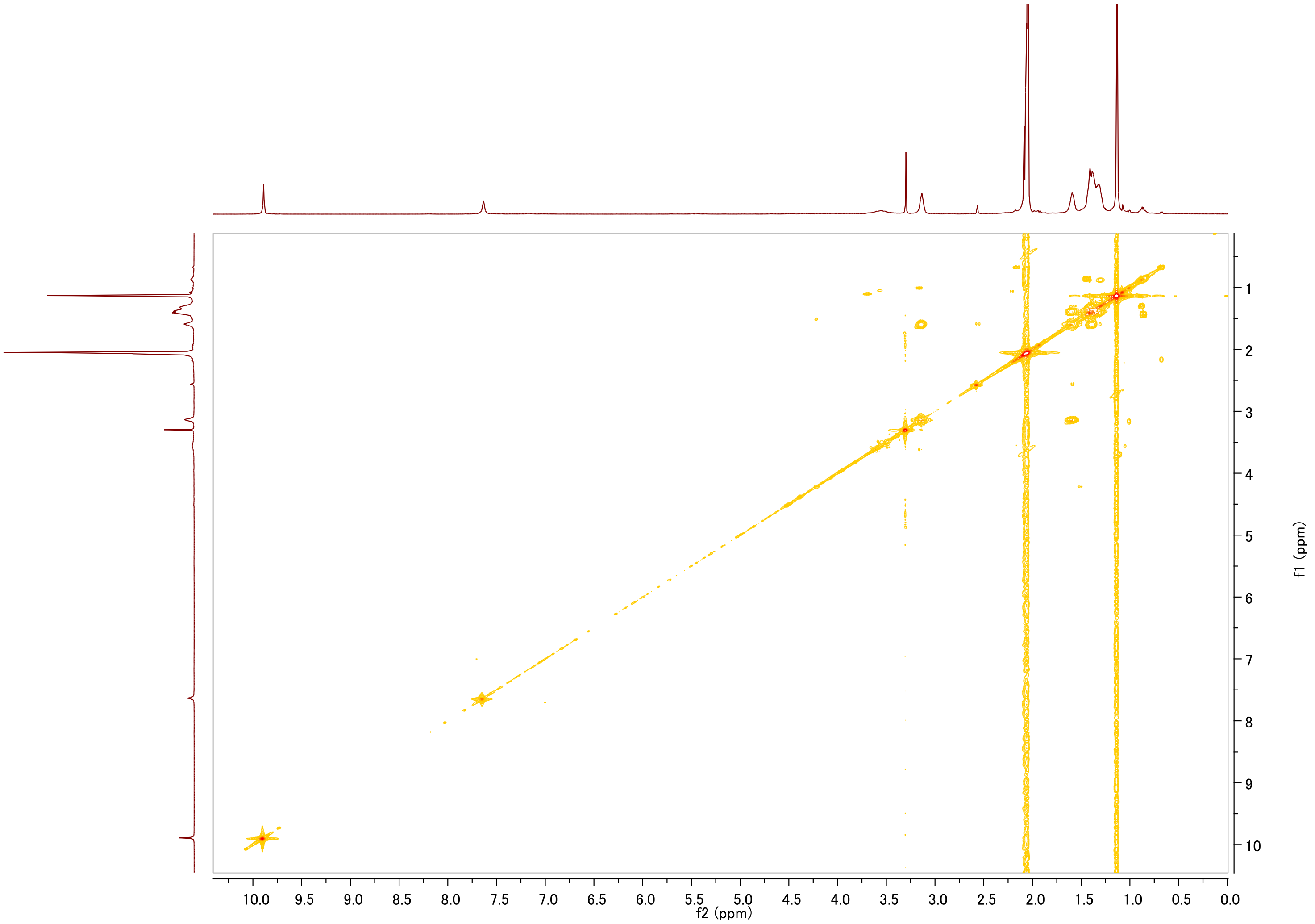

Figure S4. HSQC spectrum of **1** (500 MHz, CD_3COCD_3)

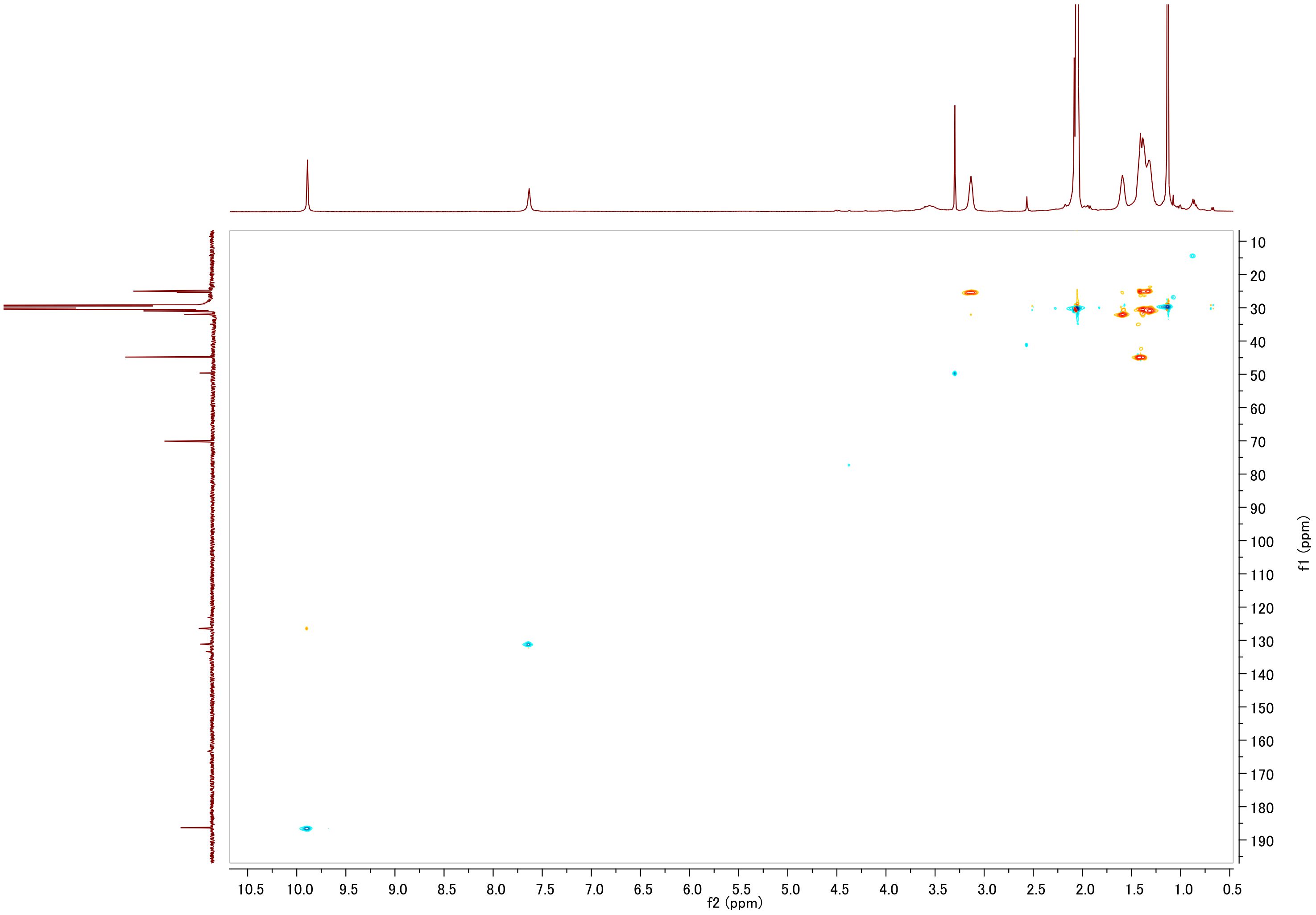

Figure S5. HMBC spectrum of **1** (500 MHz, CD_3COCD_3)

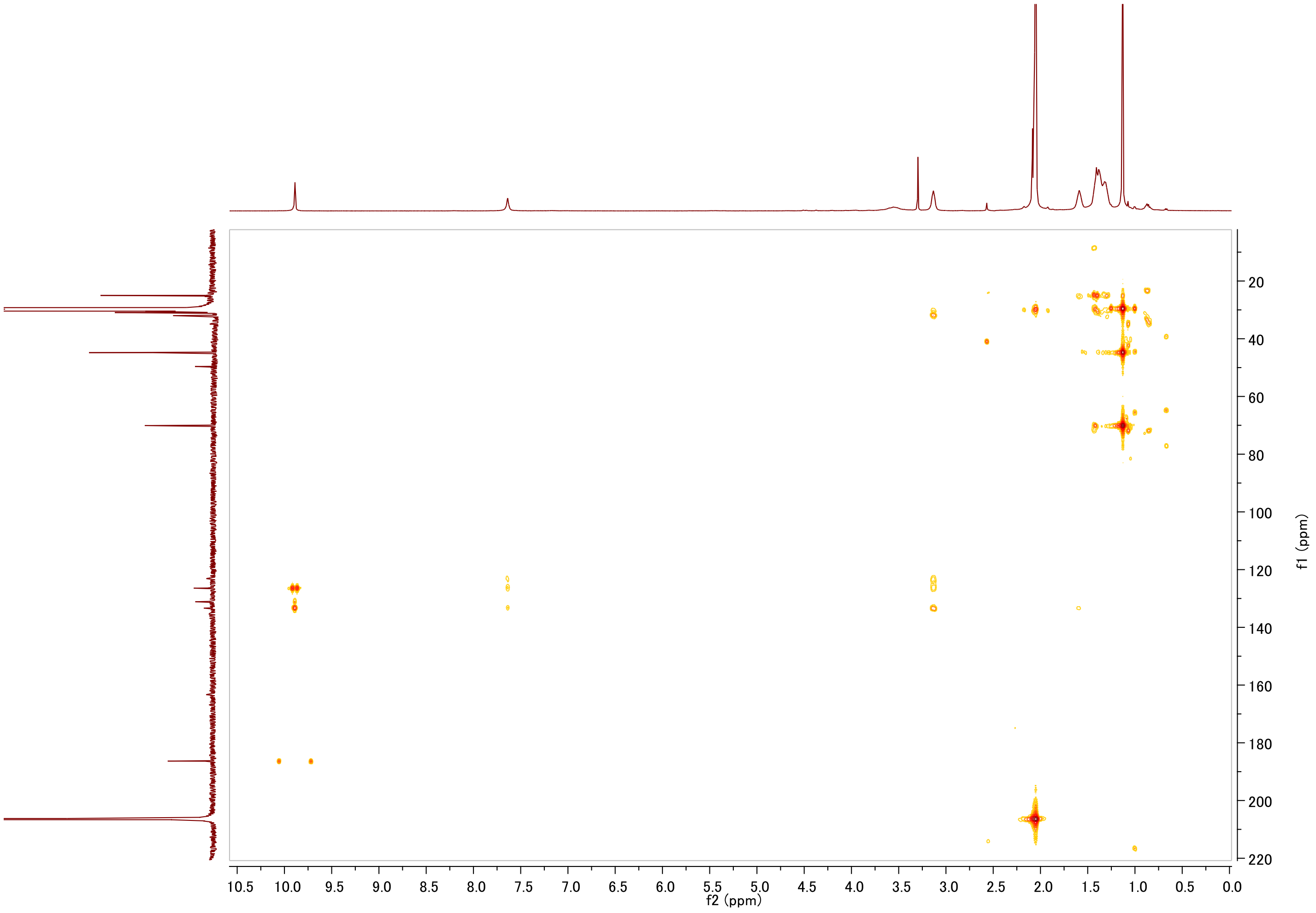

Figure S6. Negative HRESITOF mass spectrum of **1**

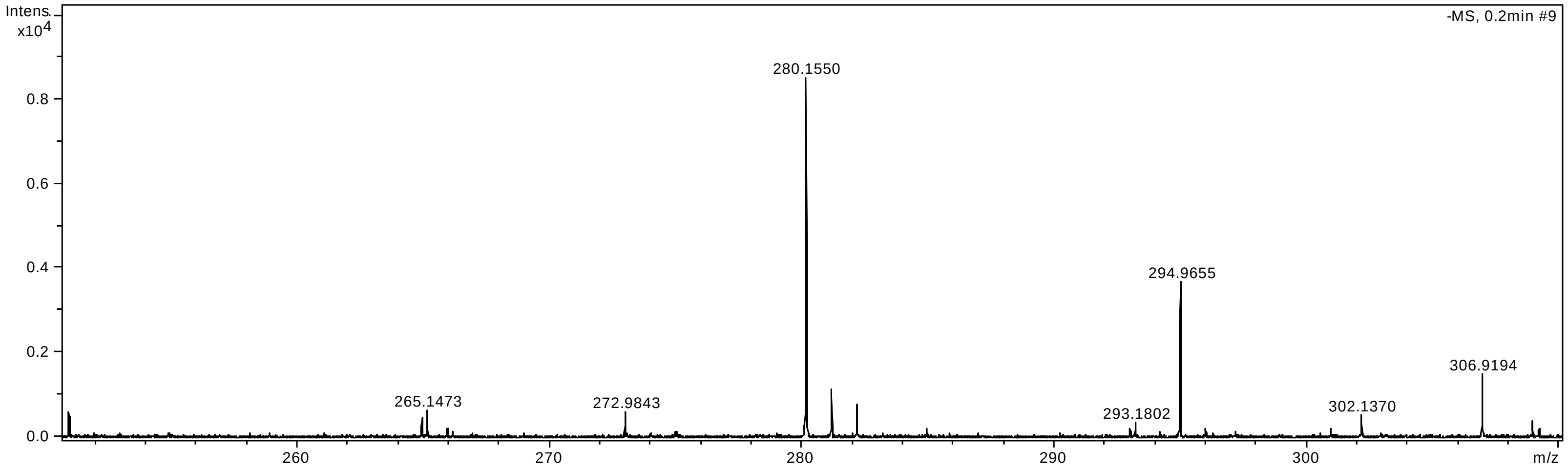

Figure S7. IR spectrum of **1**

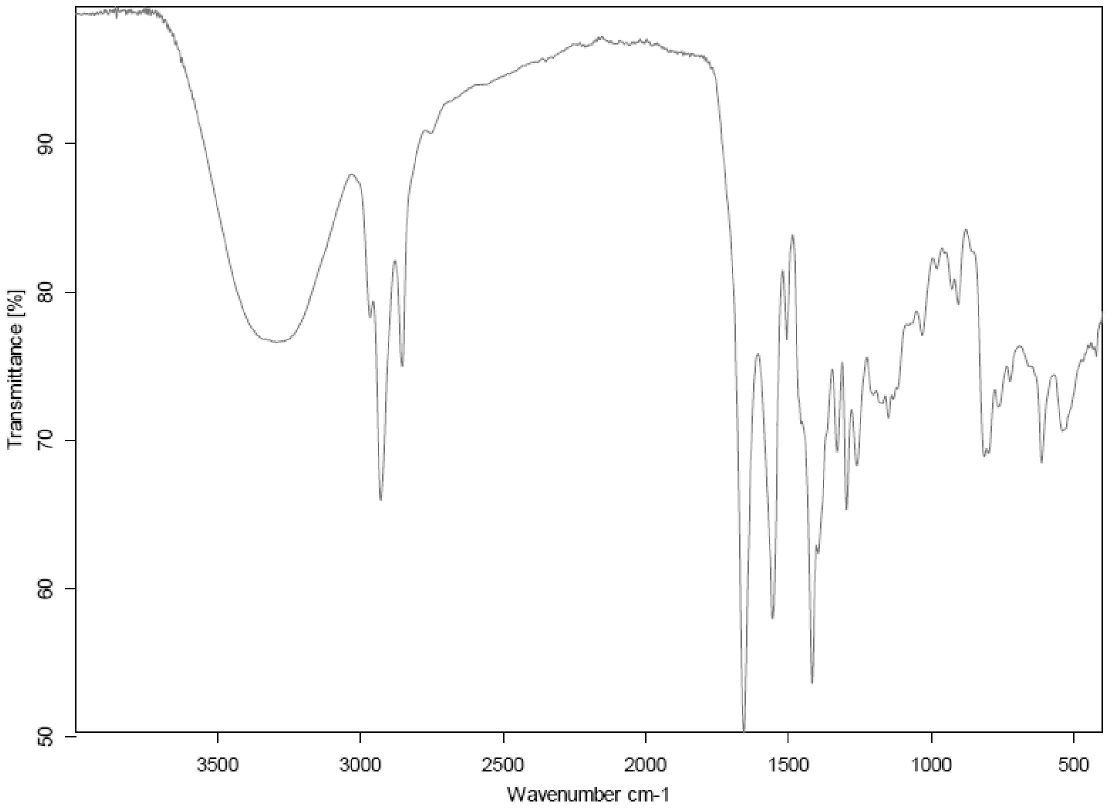

Figure S8. UV spectrum of **1**

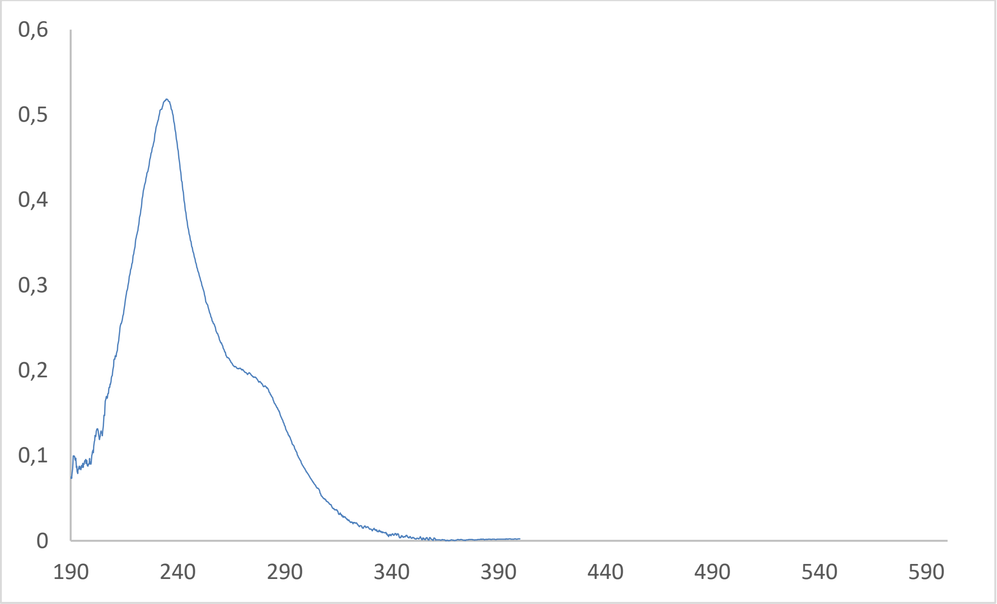

Figure S9. ^{1}H NMR spectrum of **1** (500 MHz, CD_3OD).

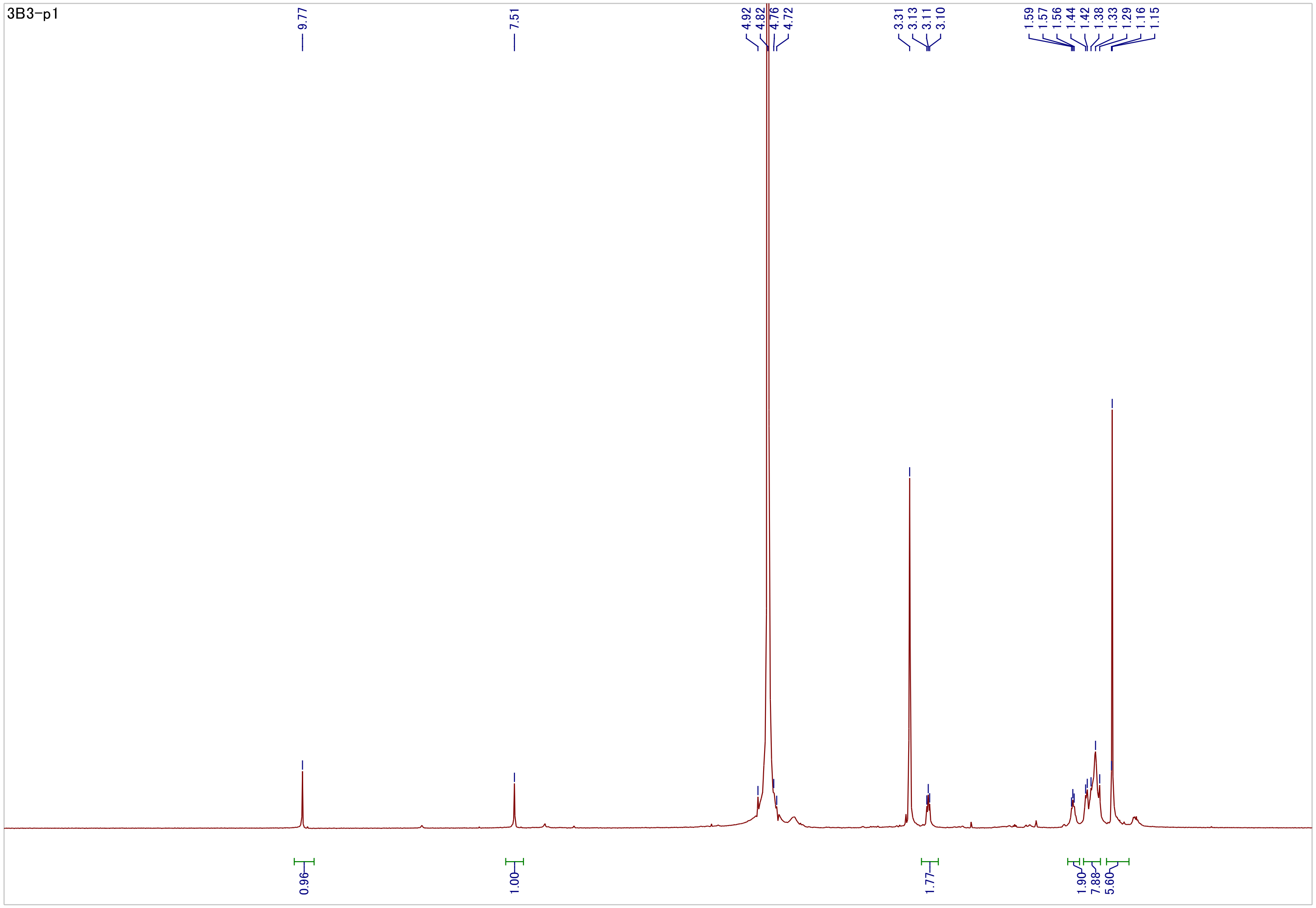

Figure S10. ^{13}C NMR spectrum of **1** (125 MHz, CD_3OD).

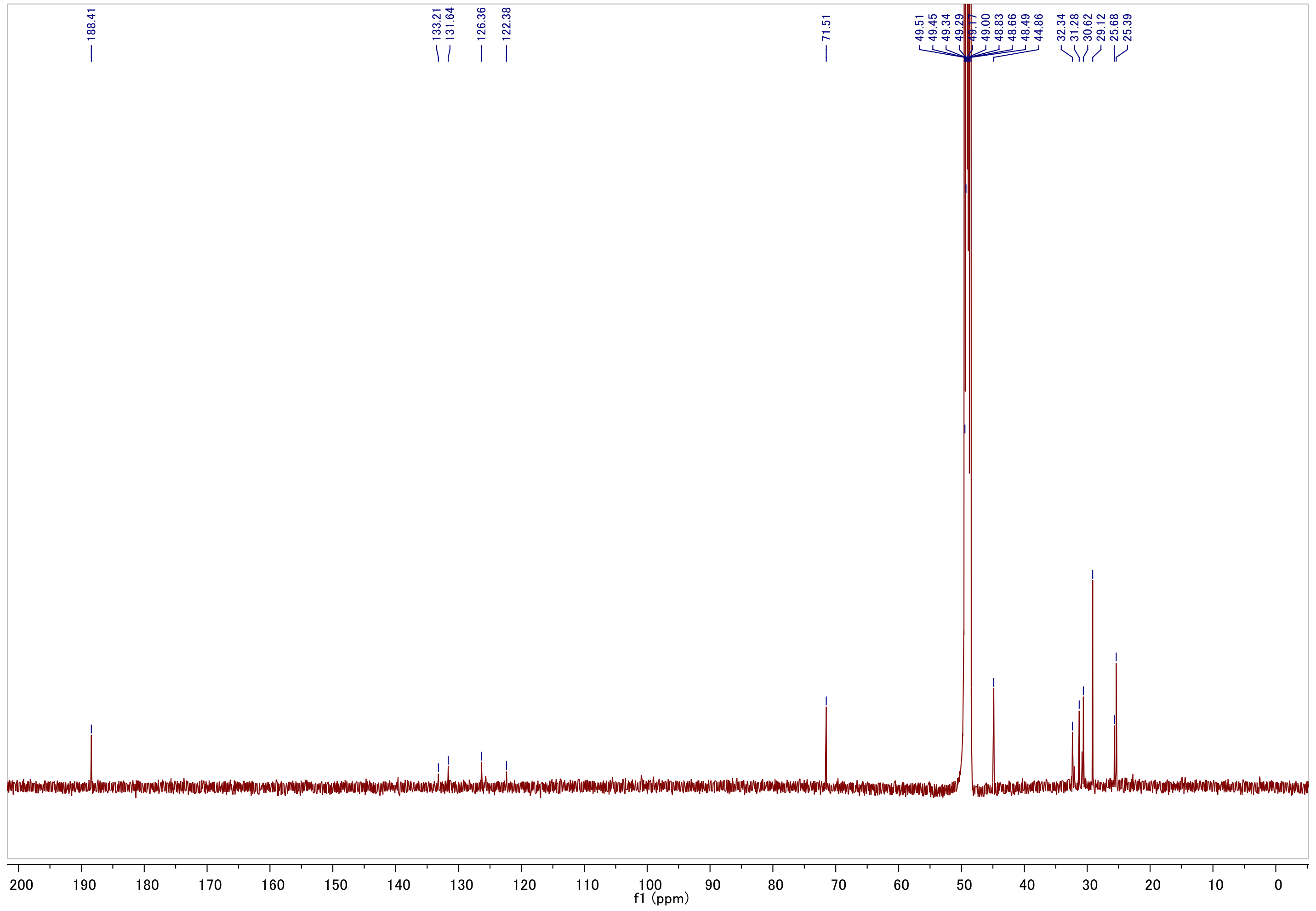

Figure S11. COSY spectrum of **1** (500 MHz, CD_3OD).

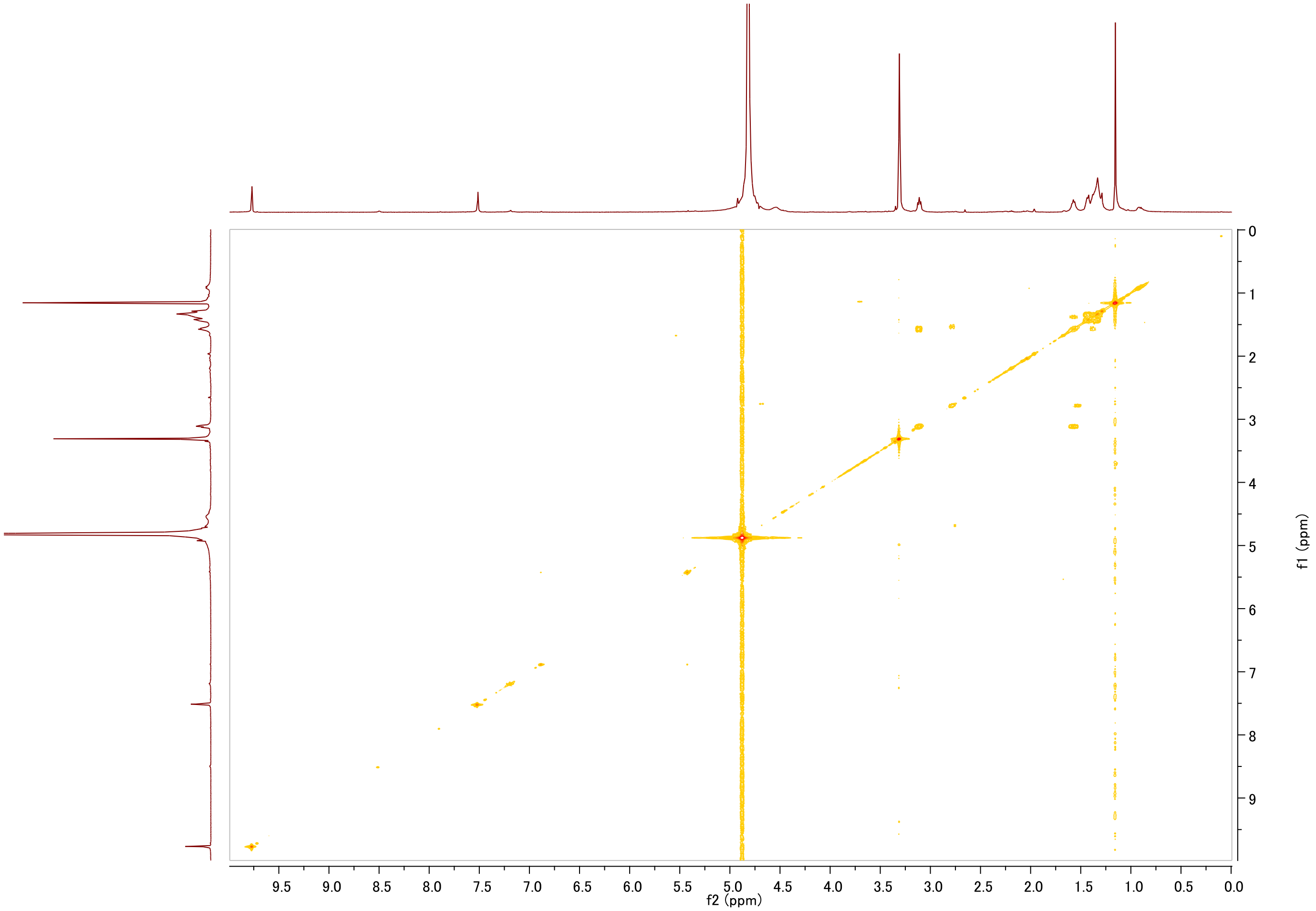

Figure S12. HSQC spectrum of **1** (500 MHz, CD_3OD).

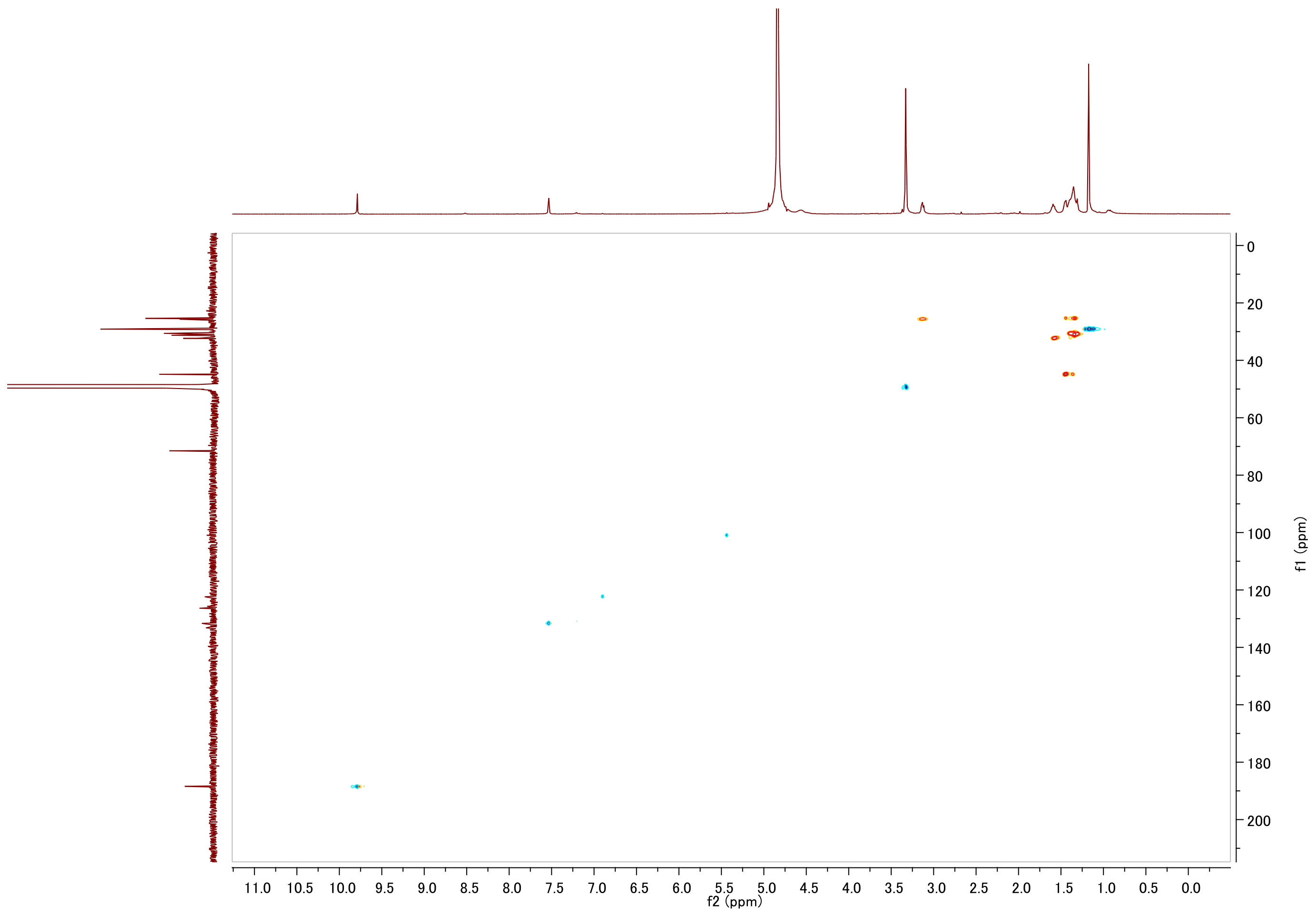

Figure S13. HMBC spectrum of **1** (500 MHz, CD_3OD).

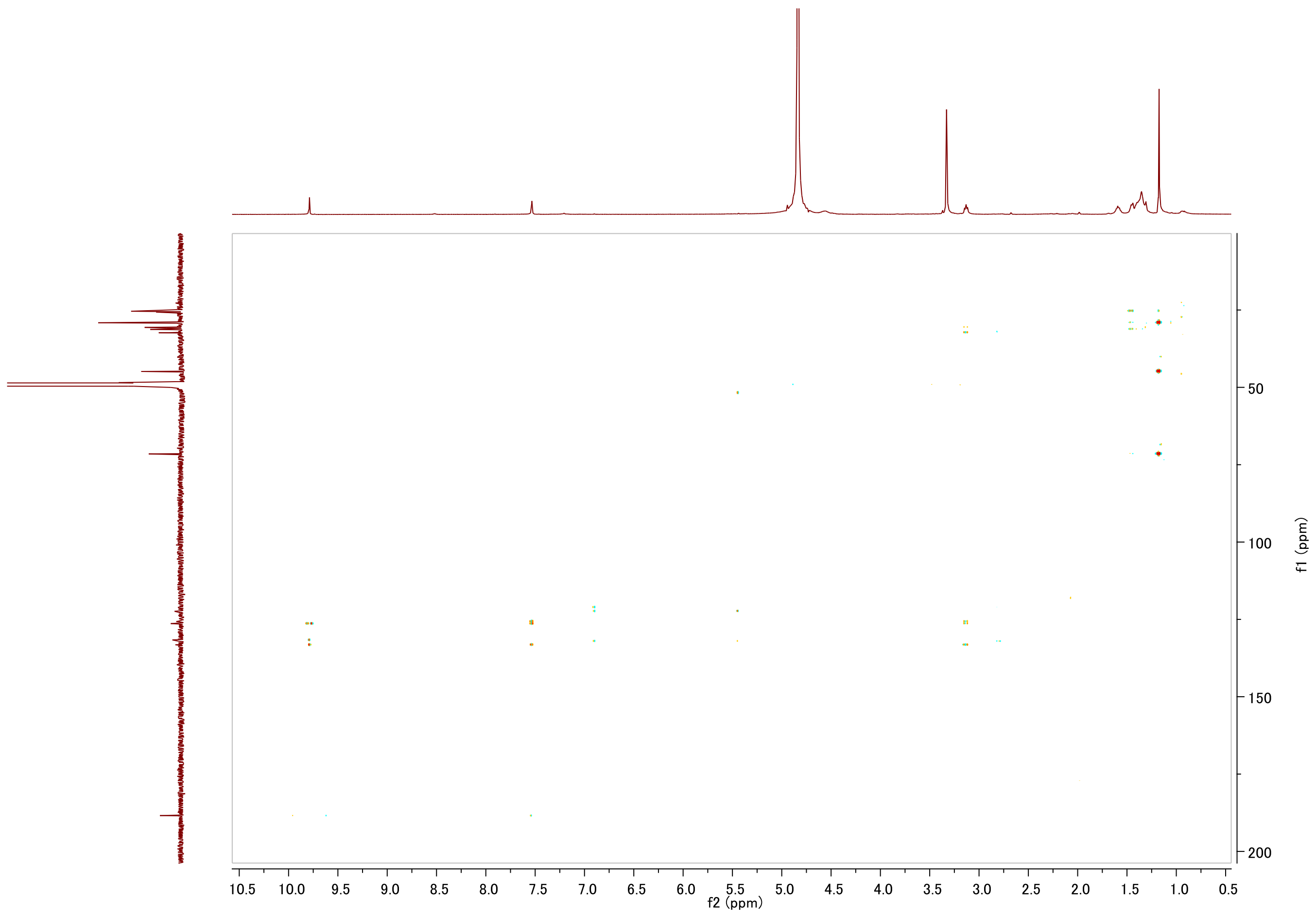

Figure S14. ^{15}N-HMBC spectrum of **1** (500 MHz, CD_3OD).

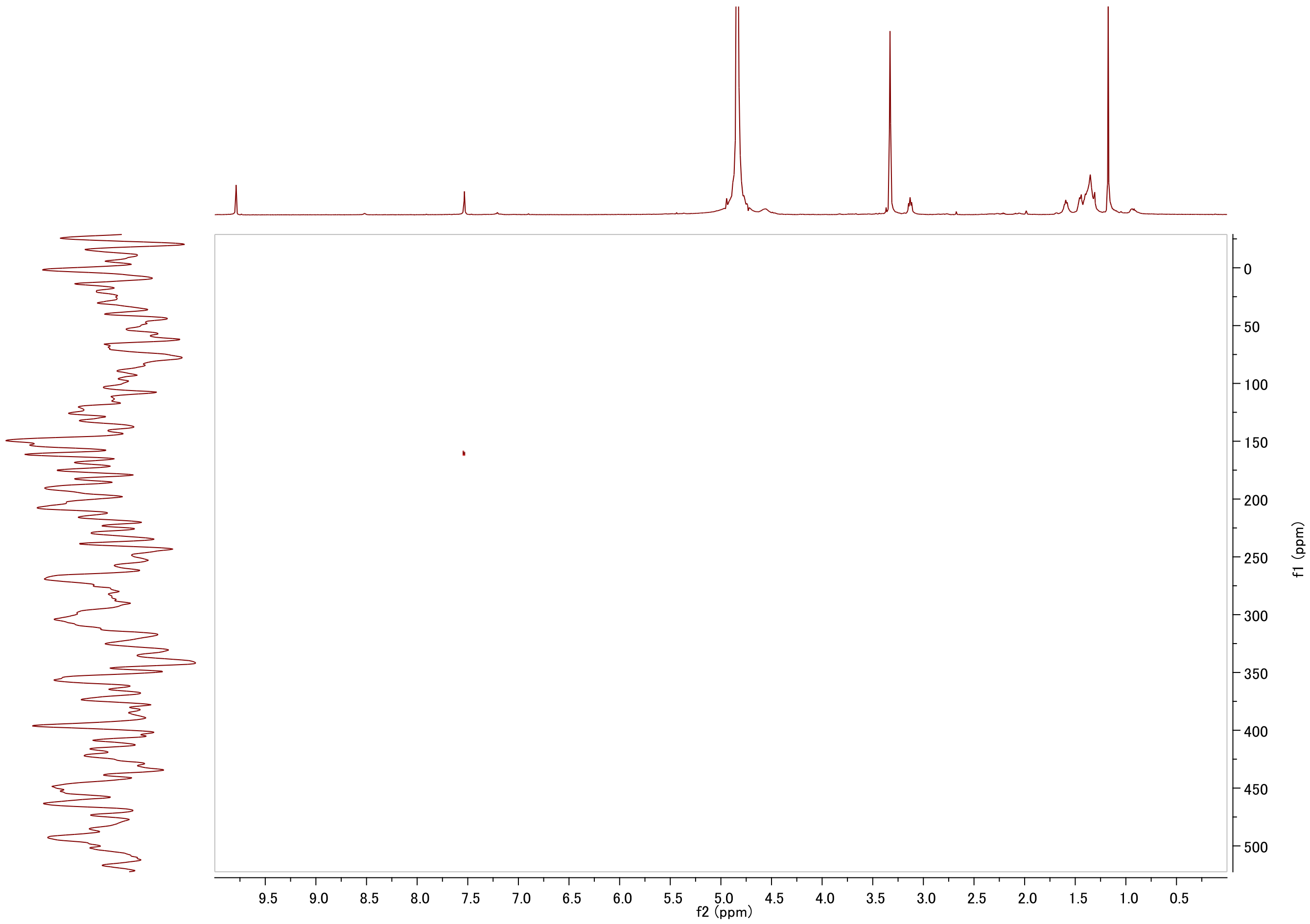

Figure S15. ^{1}H NMR spectrum of **1a** (500 MHz, CD_3COCD_3)

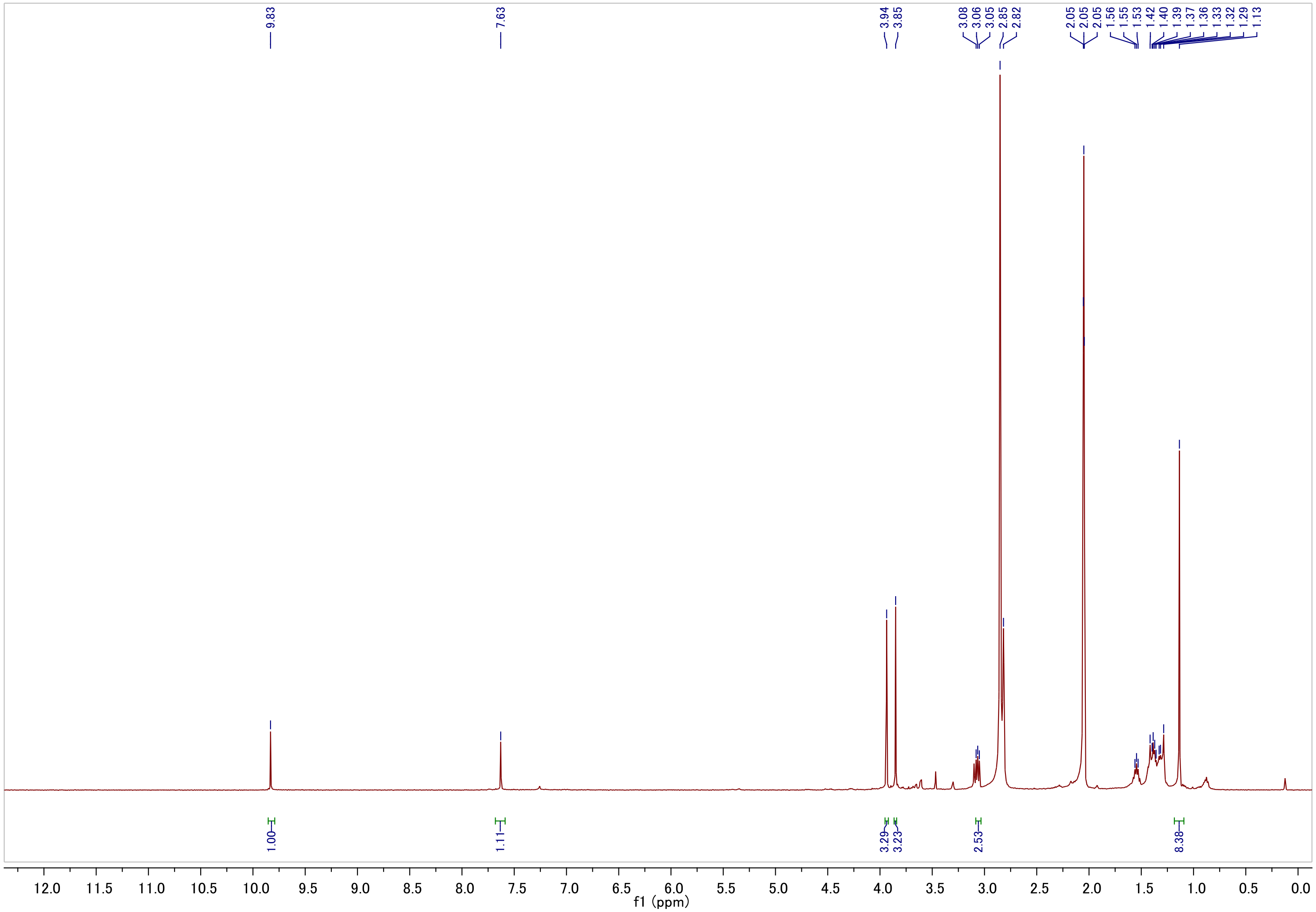

Figure S16. ^{13}C NMR spectrum of **1a** (125 MHz, CD_3COCD_3)

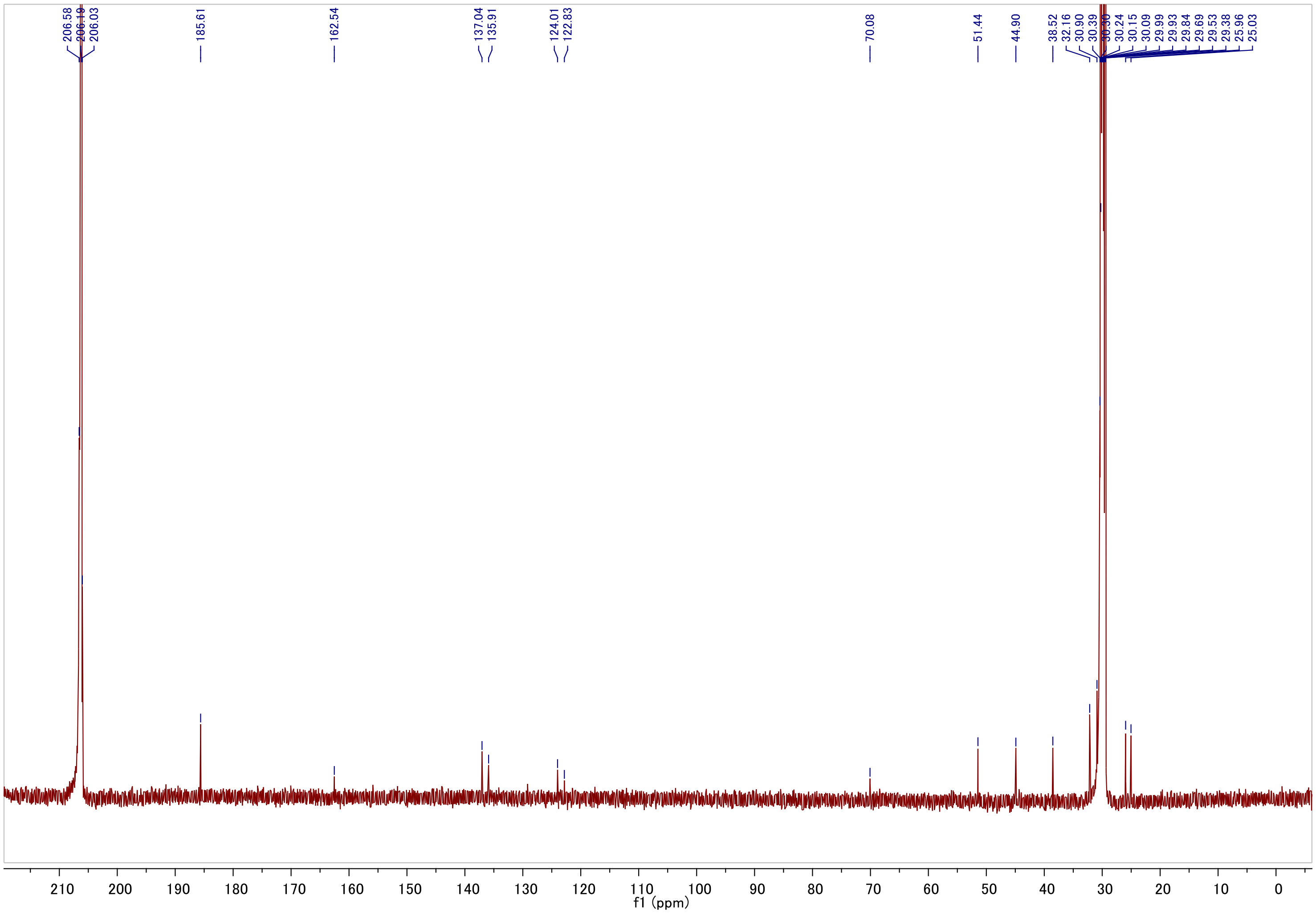

Figure S17. HSQC spectrum of **1a** (500 MHz, CD_3COCD_3)

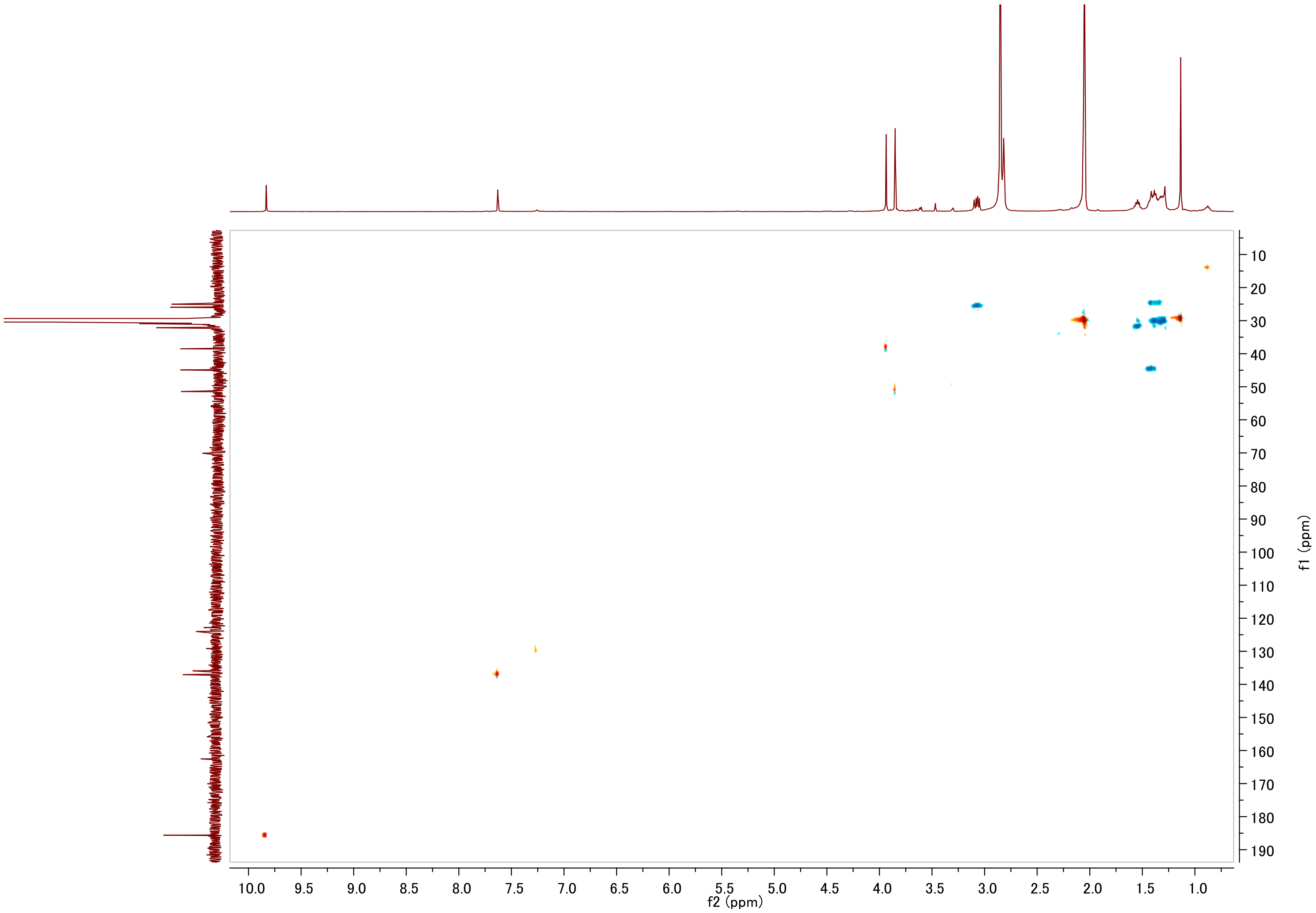

Figure S18. HMBC spectrum of **1a** (500 MHz, CD_3COCD_3)

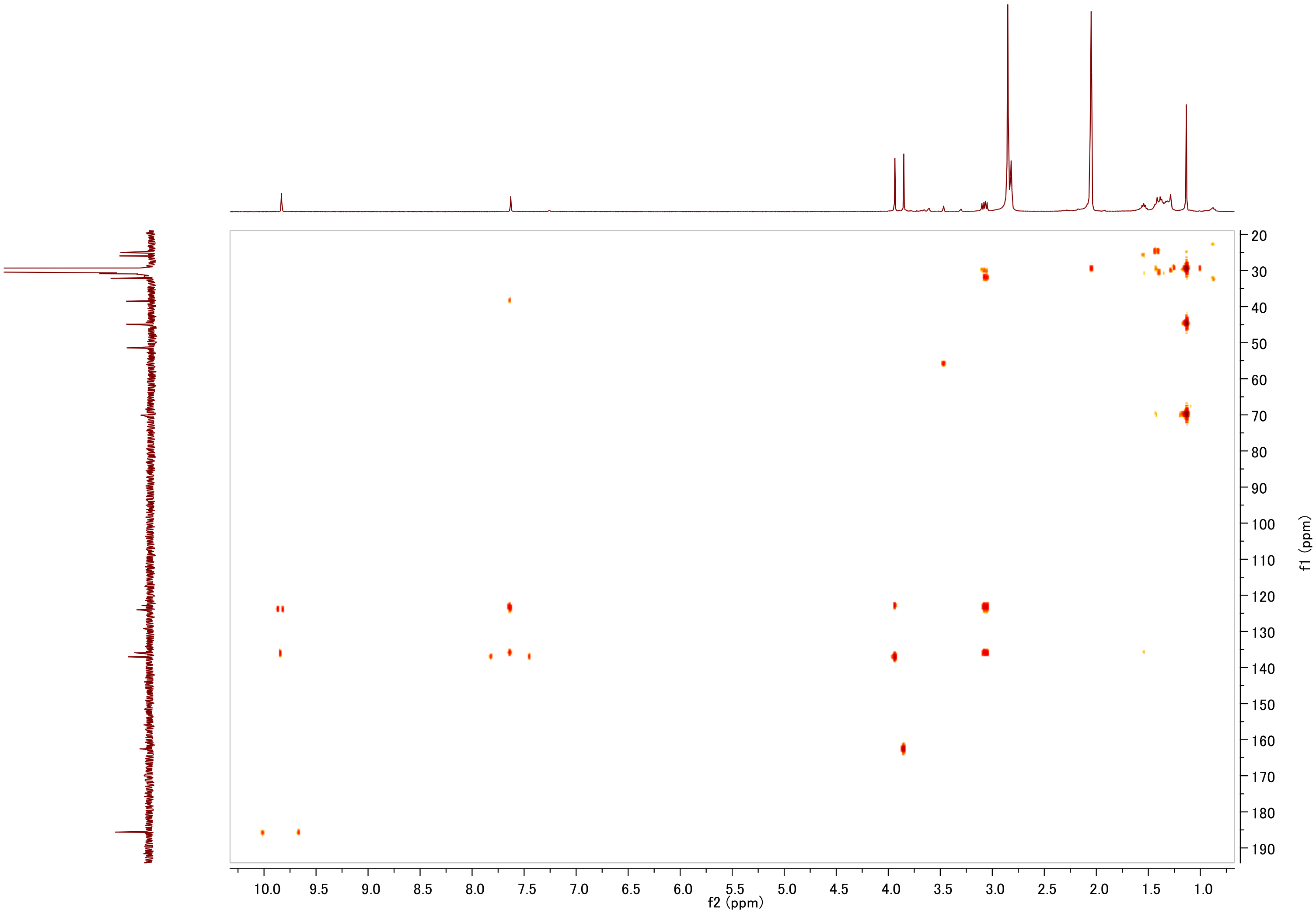

Figure S19. Positive ESITOF mass spectrum of **1a**

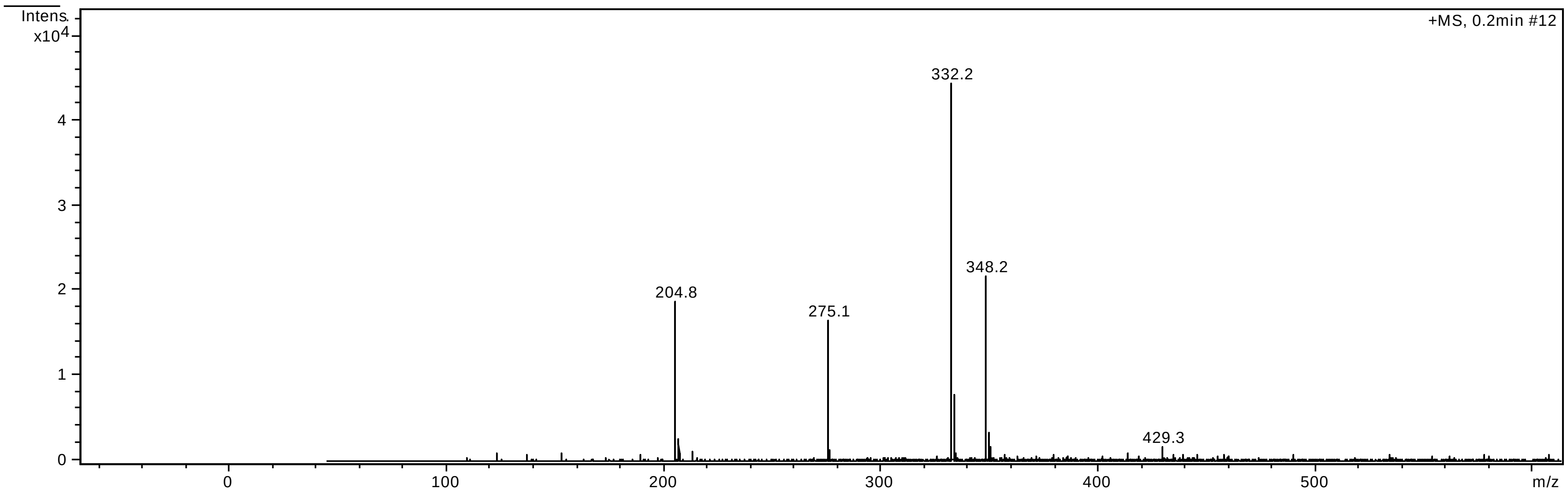

Figure S20. ^{1}H NMR spectrum of **2** (500 MHz, CD_3COCD_3)

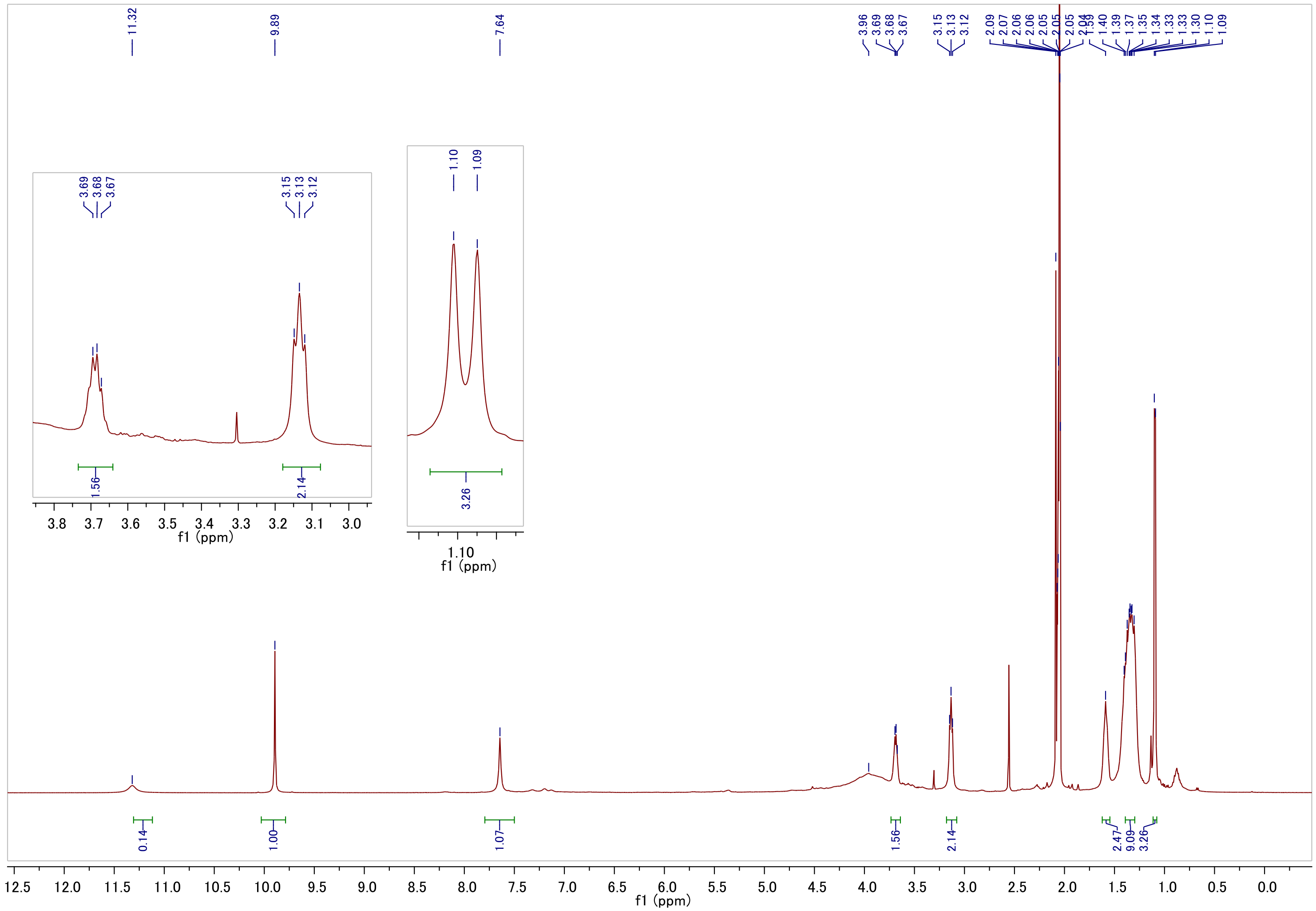

Figure S21. ^{13}C NMR spectrum of **2** (125 MHz, CD_3COCD_3)

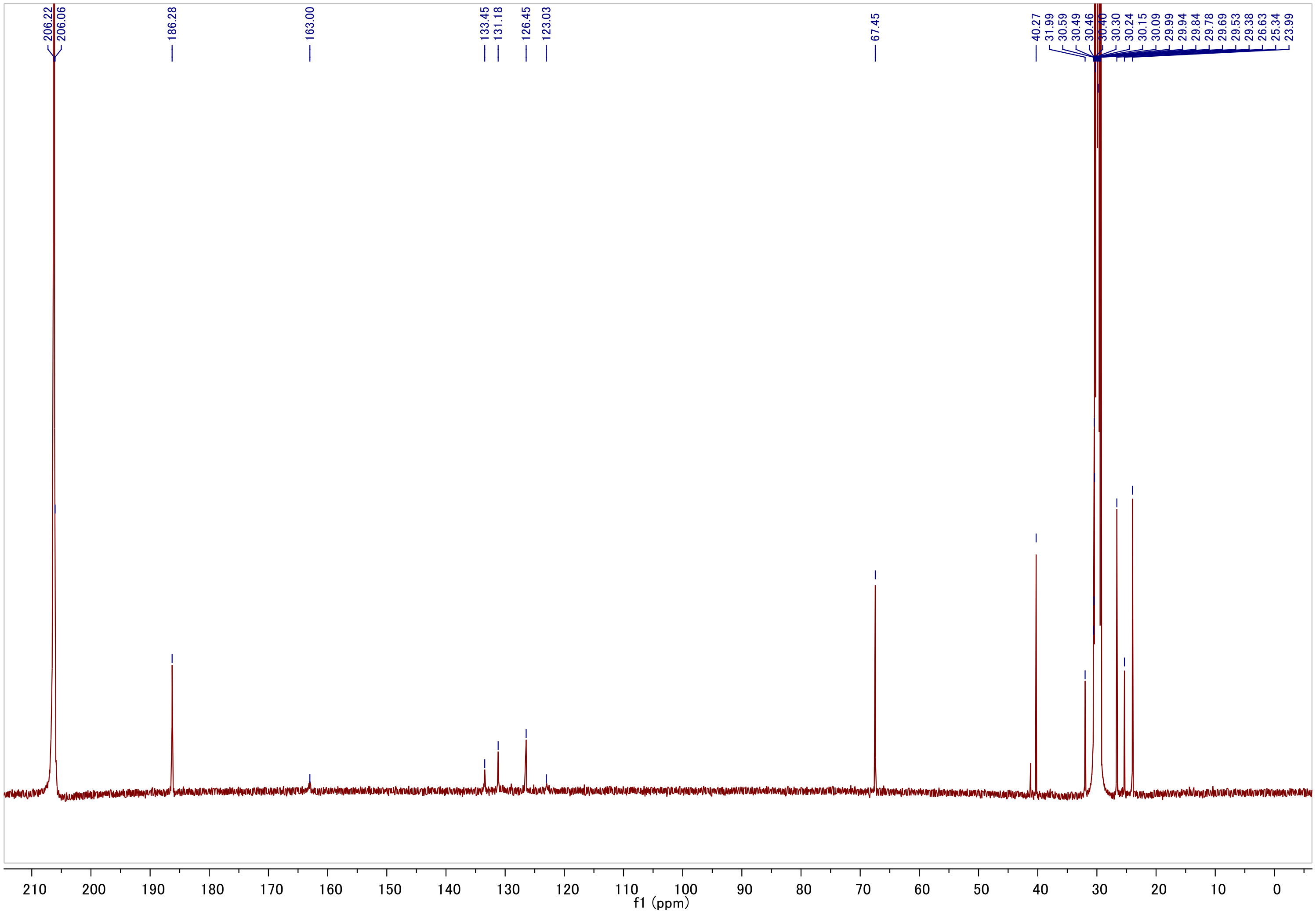

Figure S22. COSY spectrum of **2** (500 MHz, CD_3COCD_3)

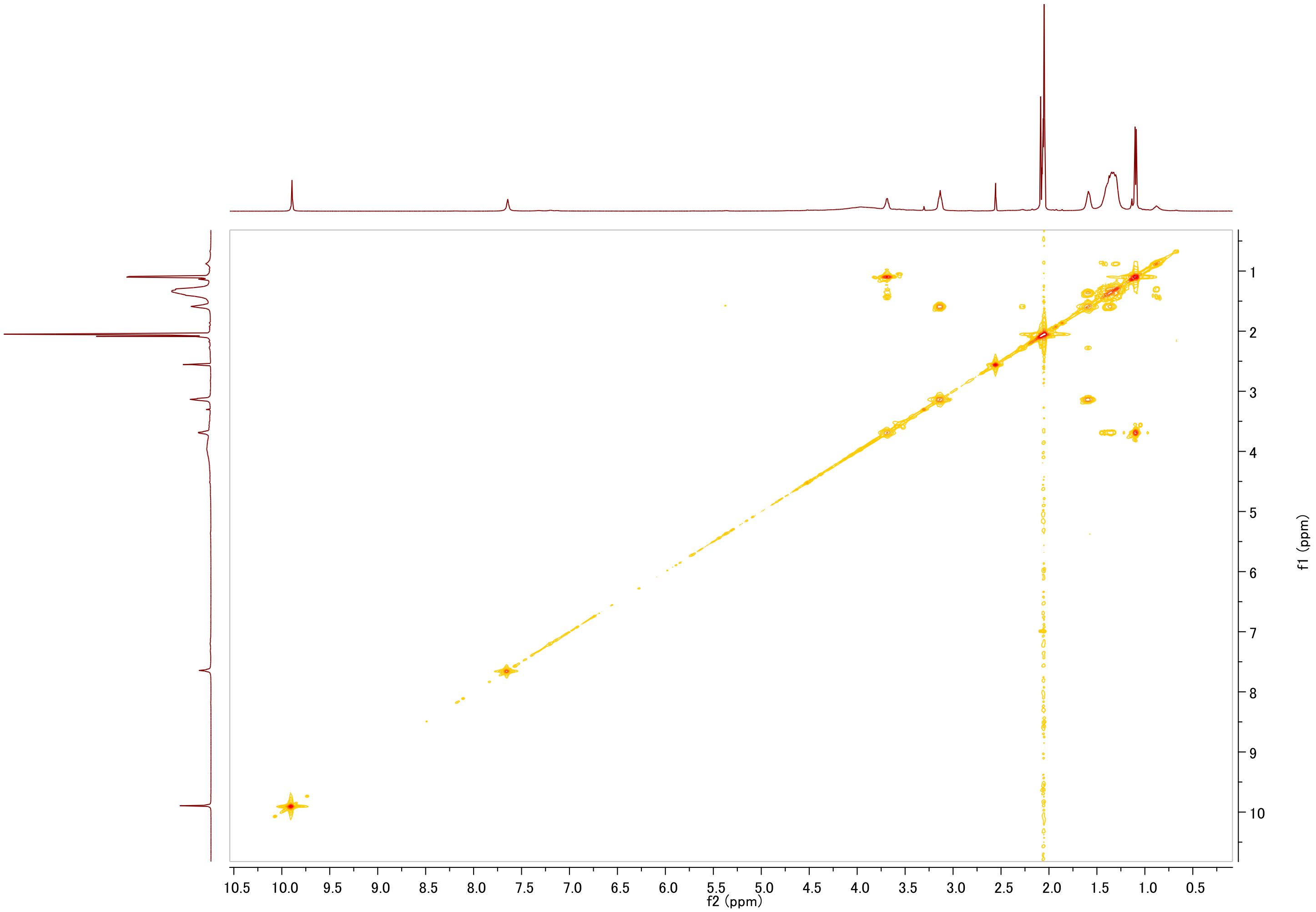

Figure S23. HSQC spectrum of **2** (500 MHz, CD_3COCD_3)

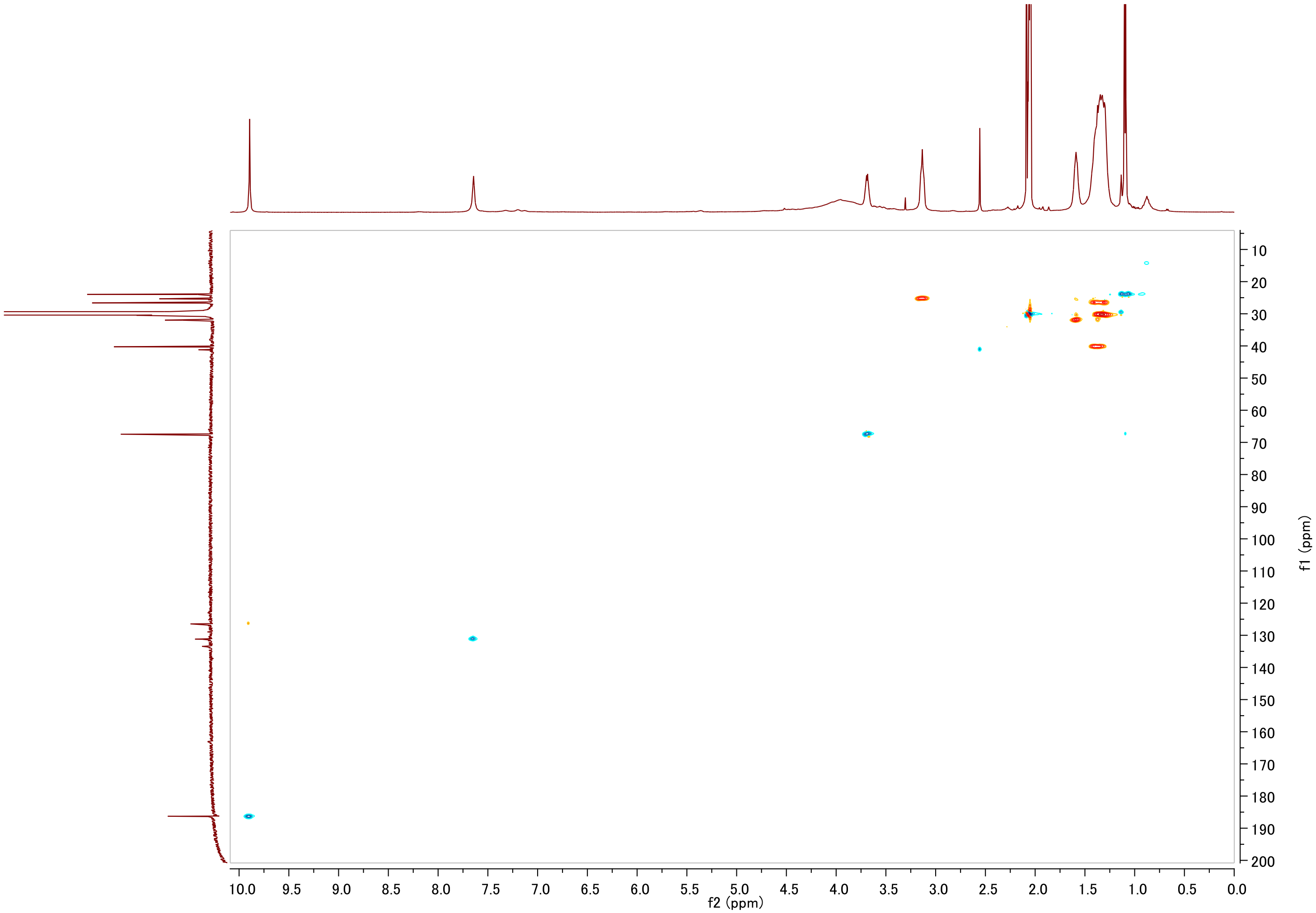

Figure S24. HMBC spectrum of **2** (500 MHz, CD_3COCD_3)

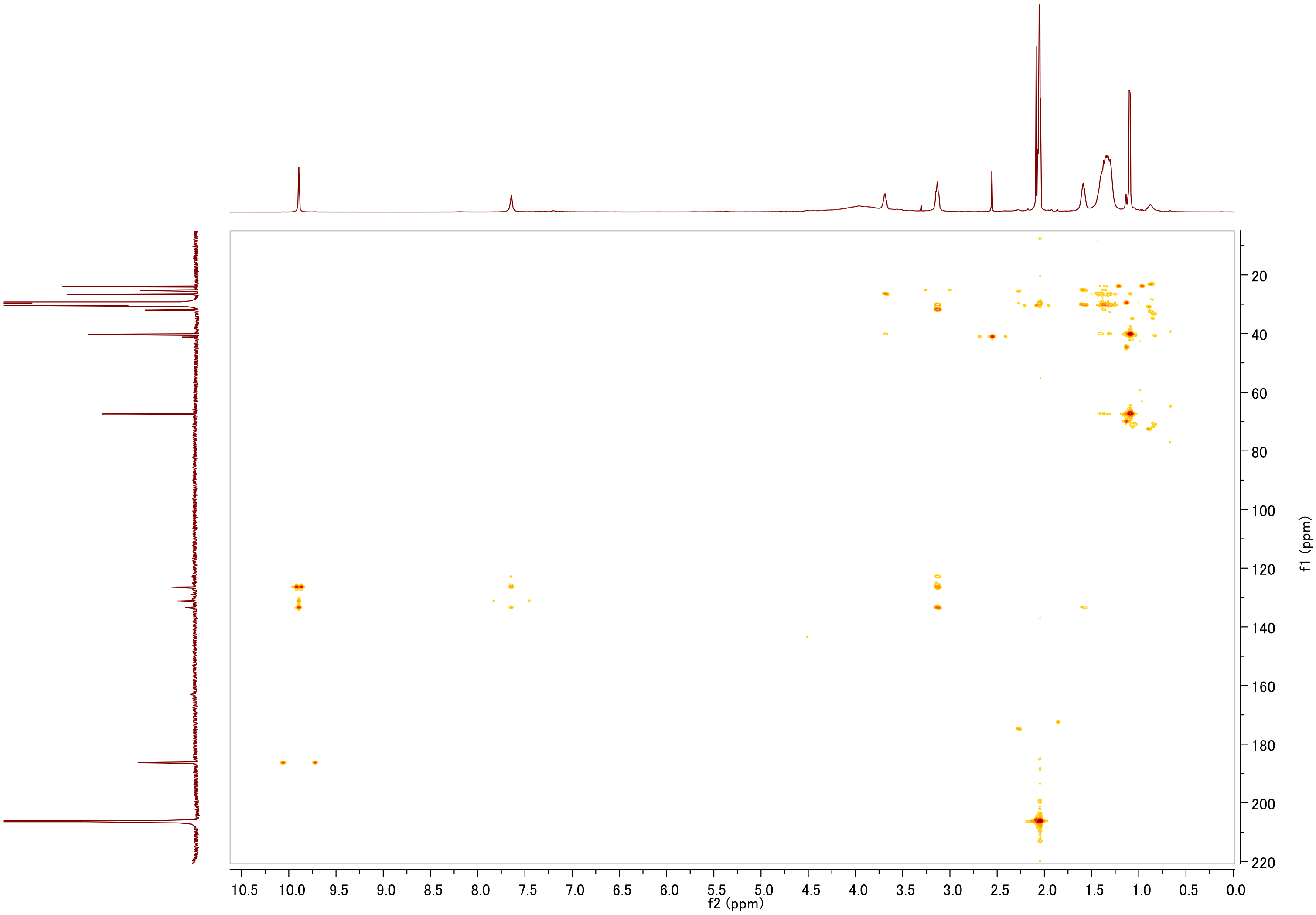

Figure S25. Negative HRESITOF mass spectrum of **2**

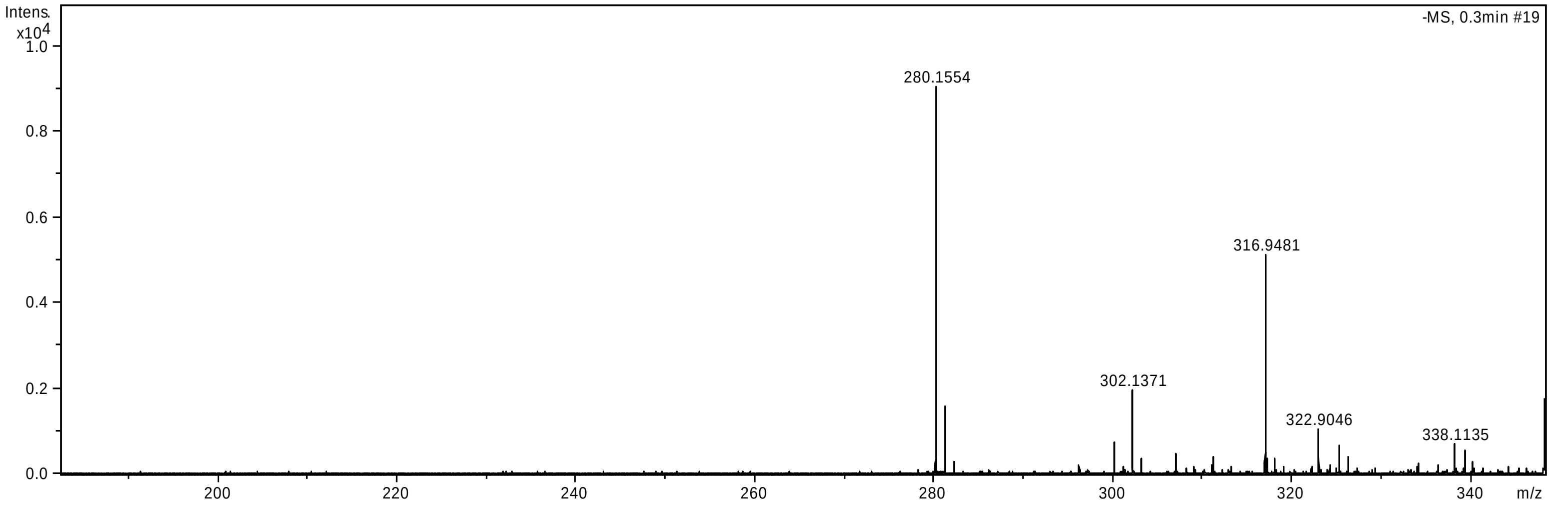

Figure S26. IR spectrum of **2**

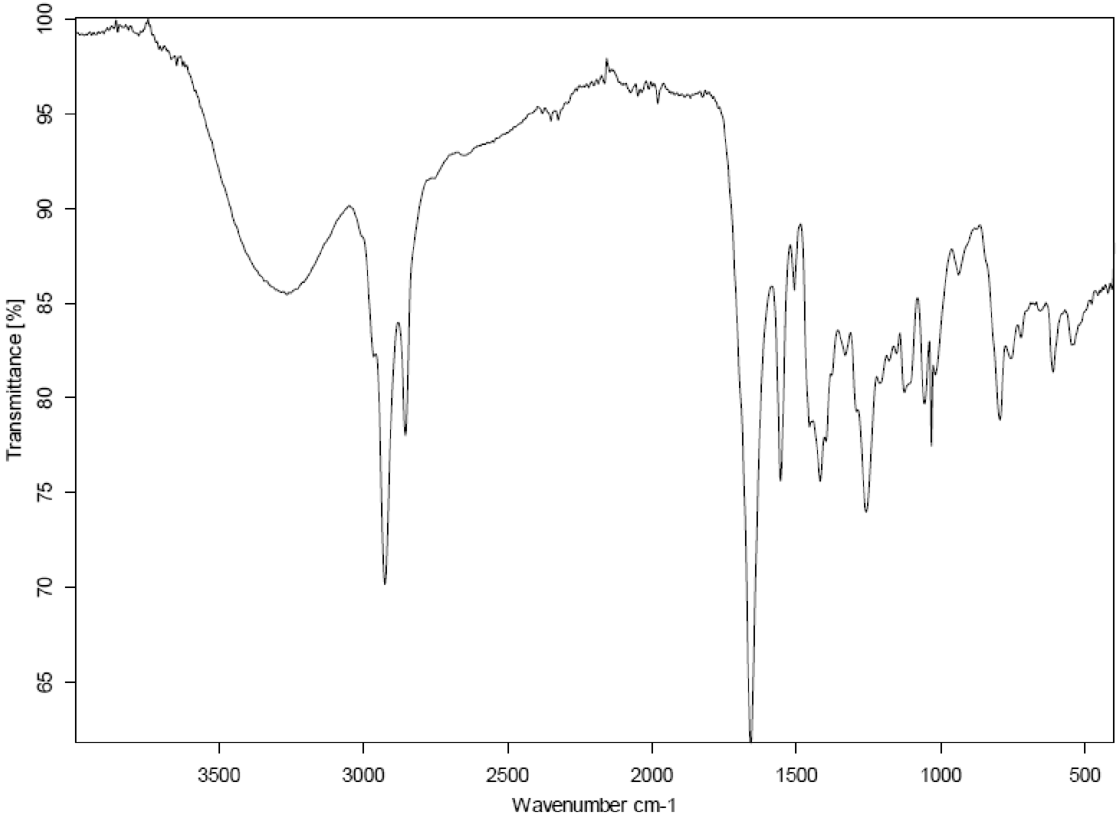

Figure S27. UV spectrum of **2**

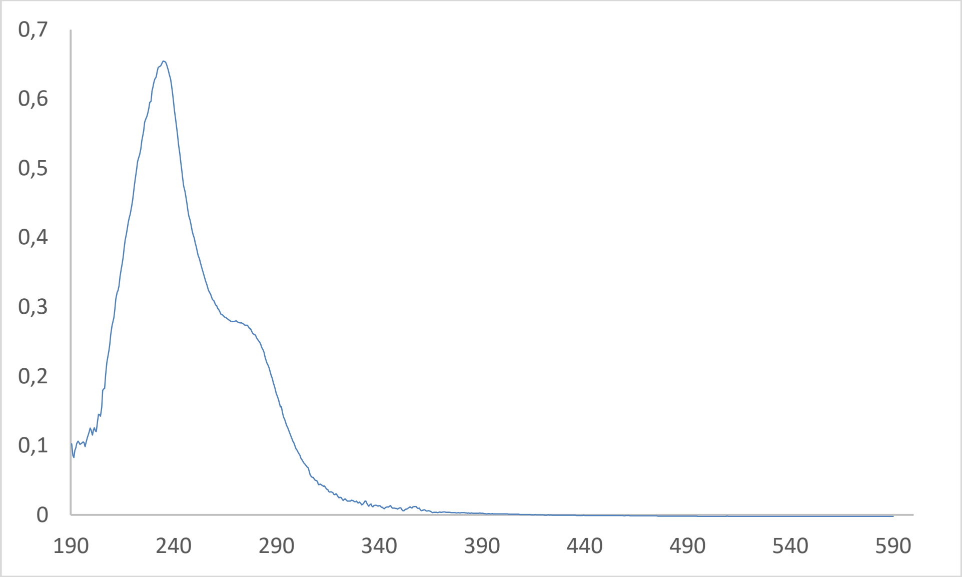

Figure S28. ^{1}H NMR spectrum of **3** (500 MHz, CD_3COCD_3)

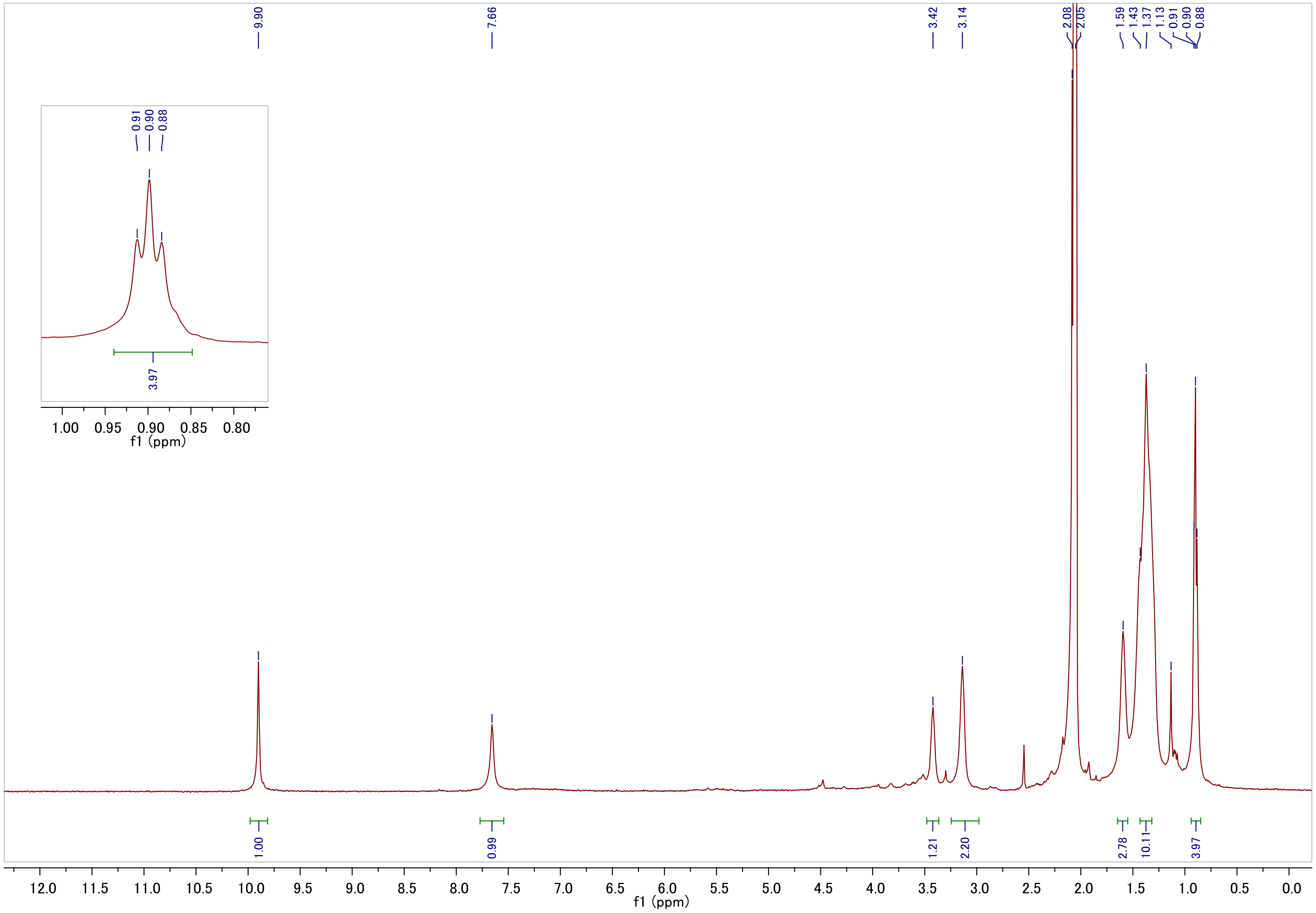

Figure S29. ^{13}C NMR spectrum of **3** (125 MHz, CD_3COCD_3)

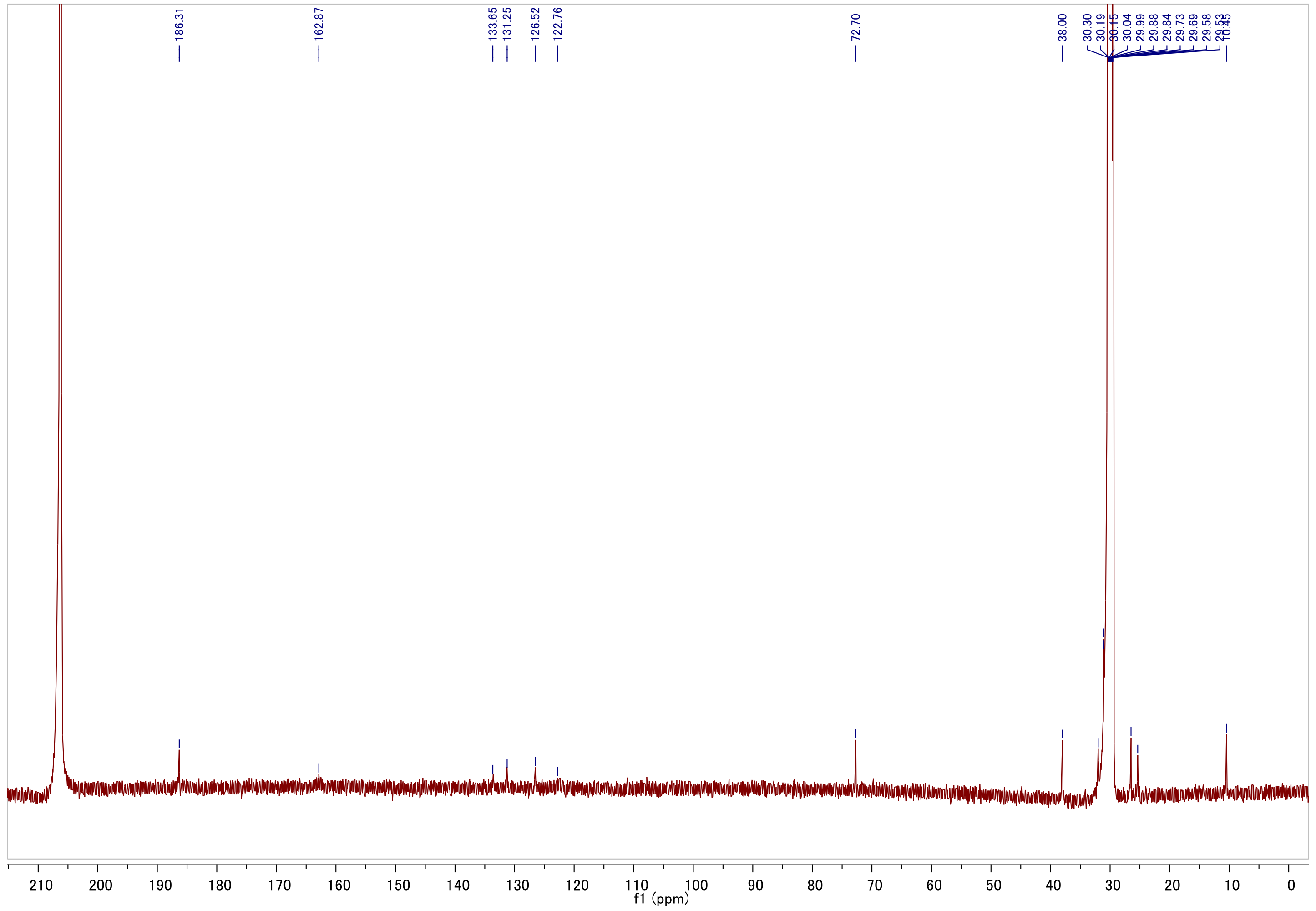

Figure S30. COSY spectrum of **3** (500 MHz, CD_3COCD_3)

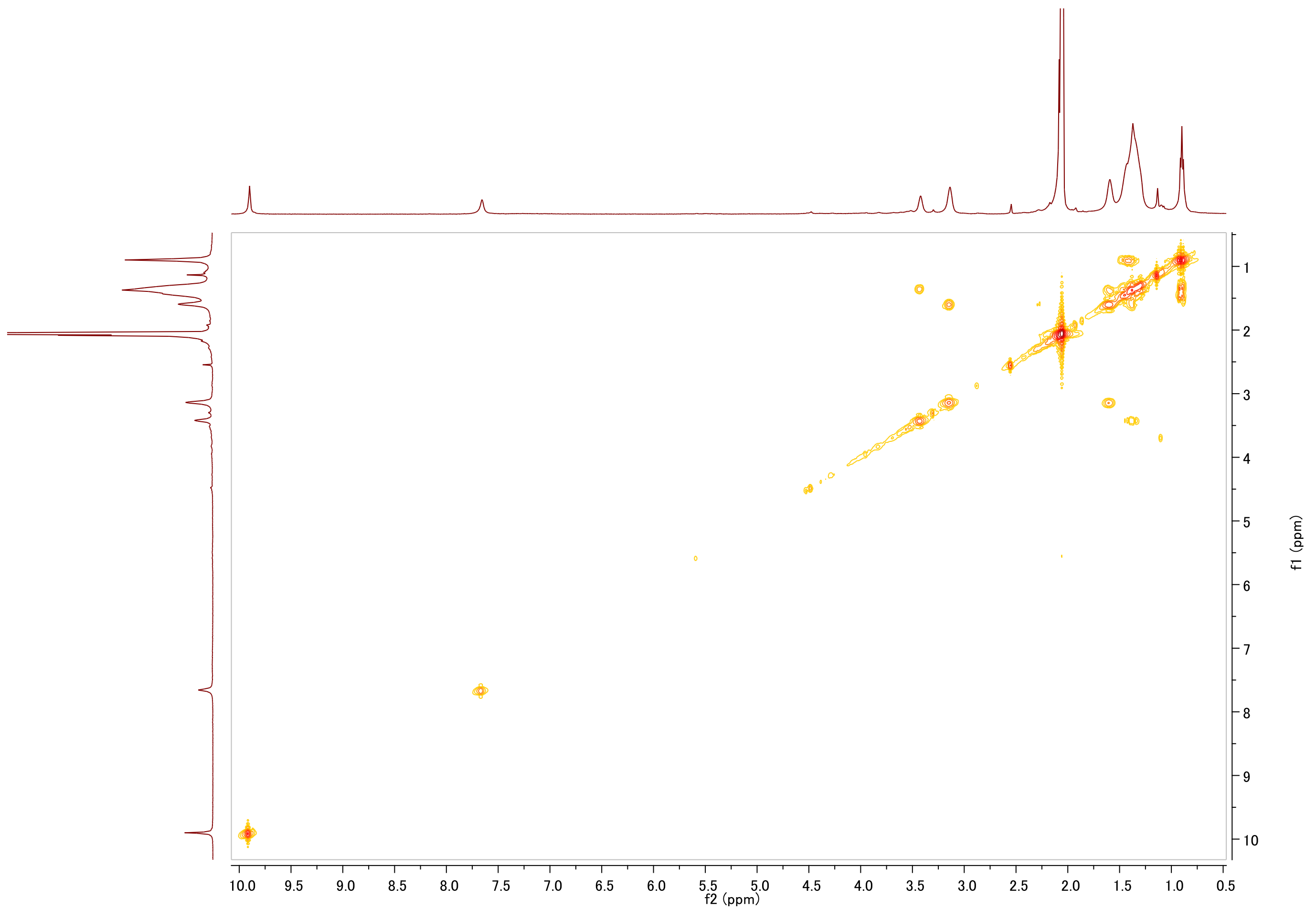

Figure S31. HSQC spectrum of **3** (500 MHz, CD_3COCD_3)

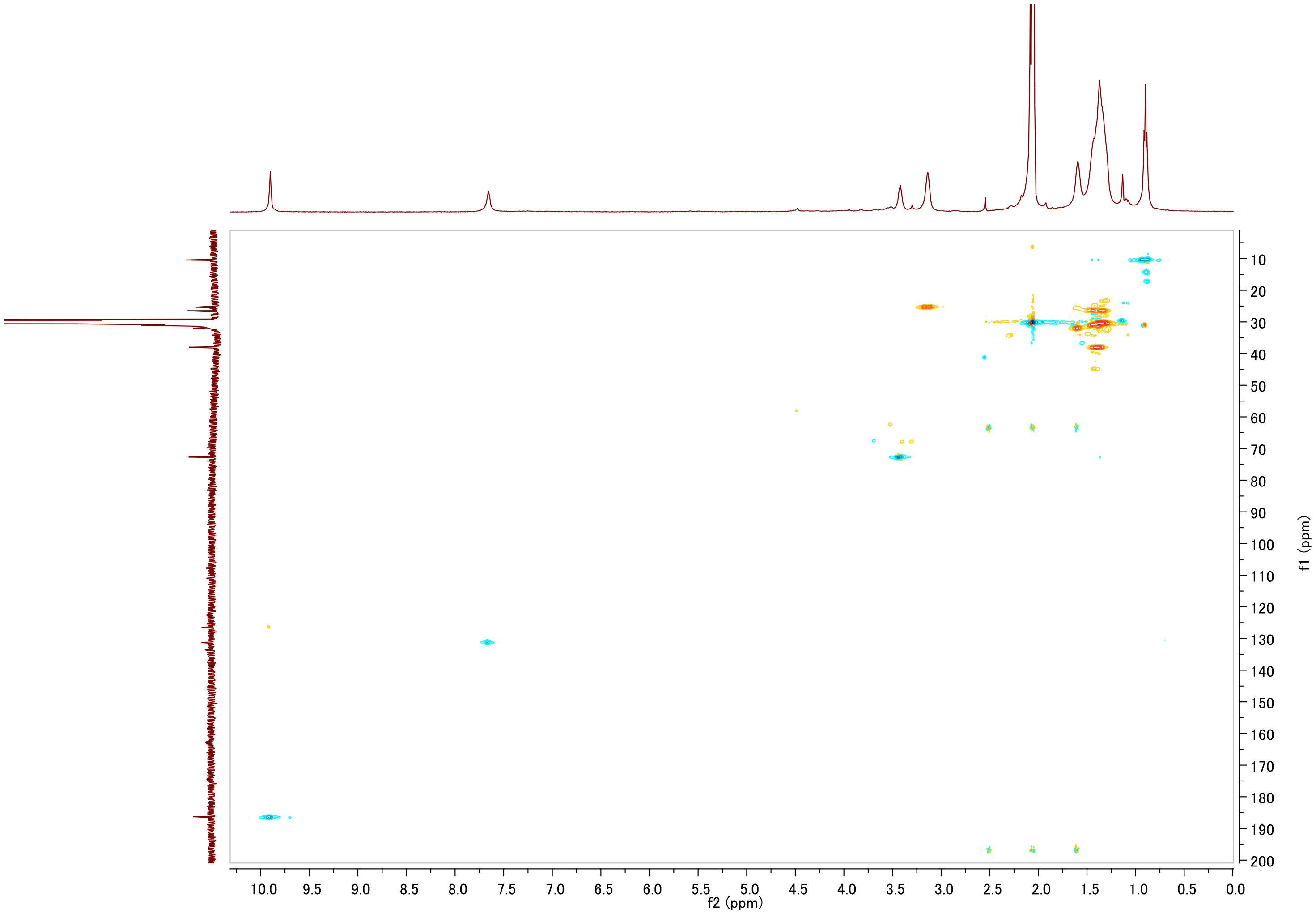

Figure S32. HMBC spectrum of **3** (500 MHz, CD_3COCD_3)

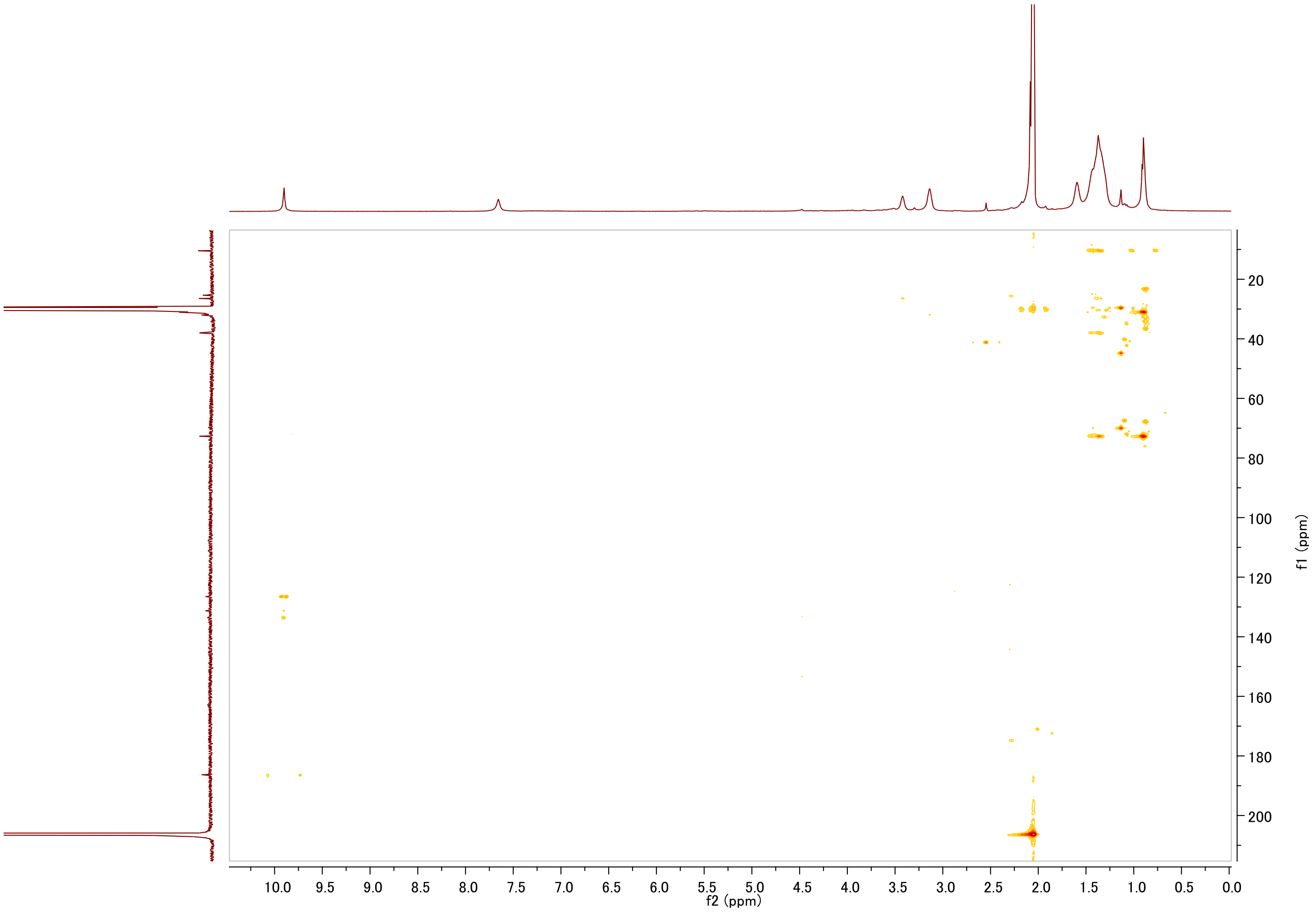

Figure S33. Negative HRESITOF mass spectrum of **3**

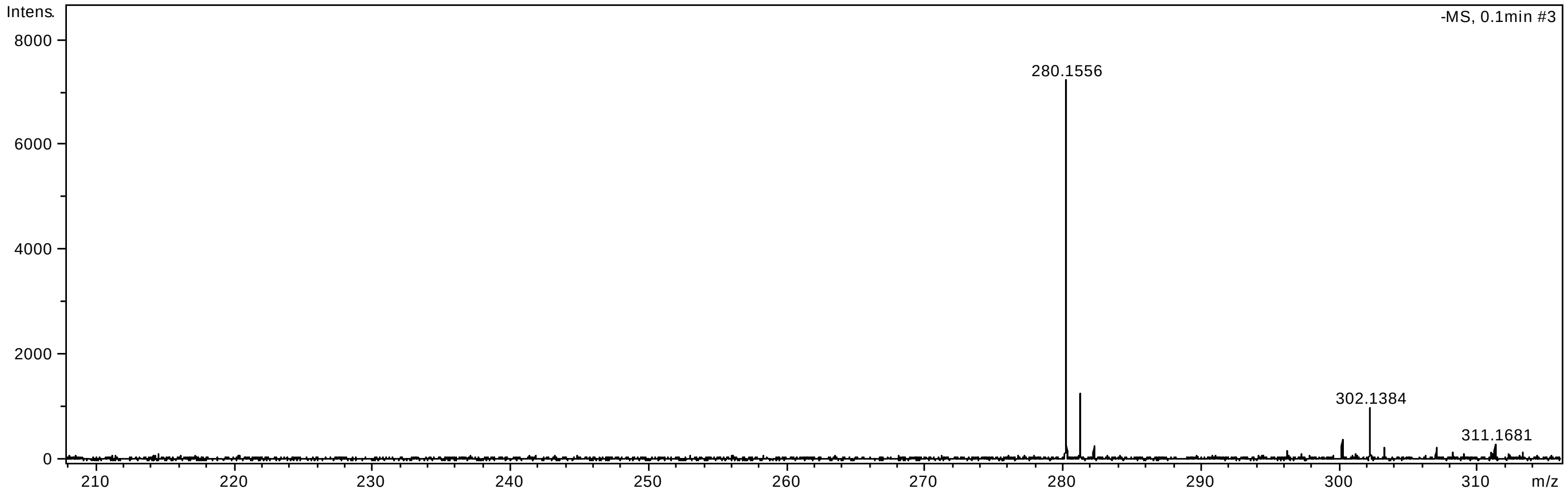

Figure S34. IR spectrum of **3**

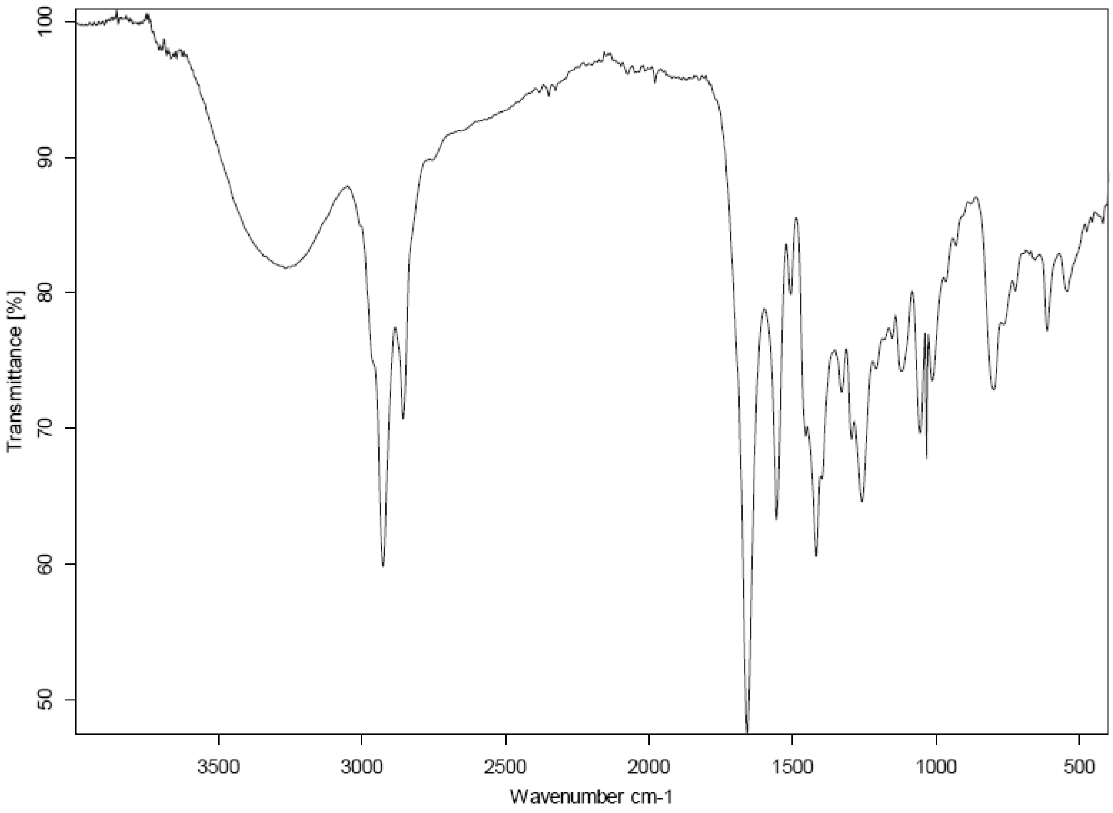

Figure S35. UV spectrum of **3**

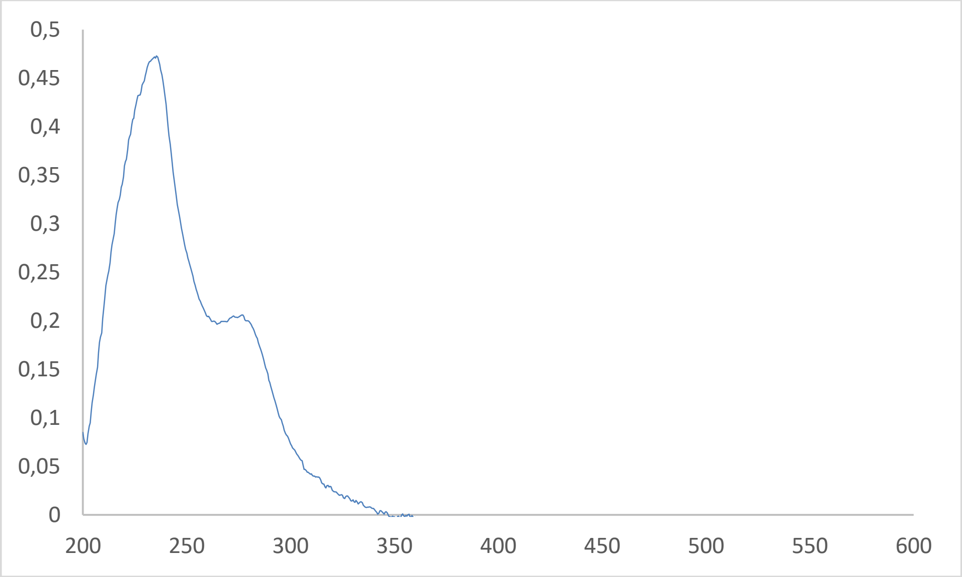

Figure S36. ^{1}H NMR spectrum of **4** (500 MHz, CD_3COCD_3)

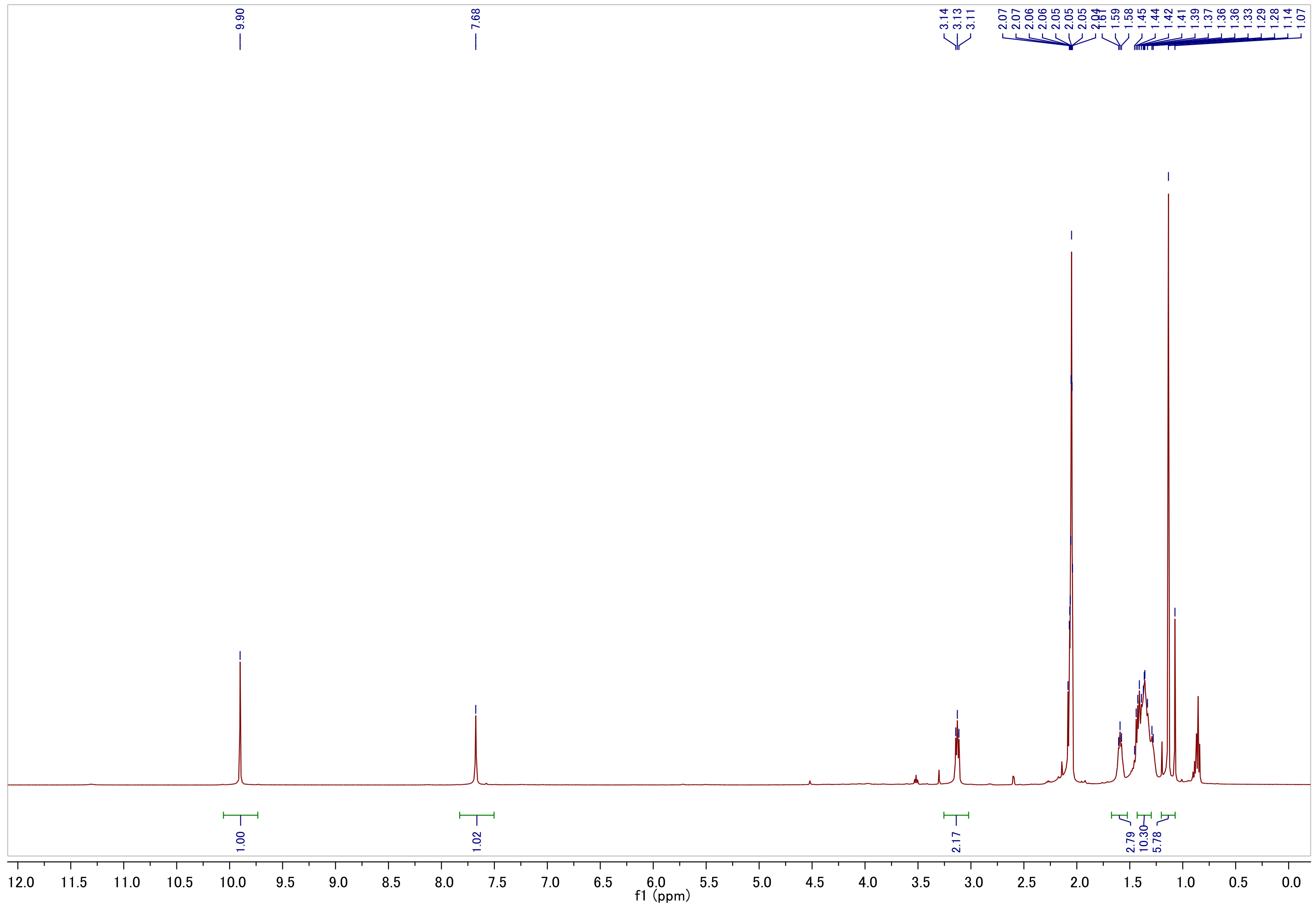

Figure S37. ^{13}C NMR spectrum of **4** (125 MHz, CD_3COCD_3)

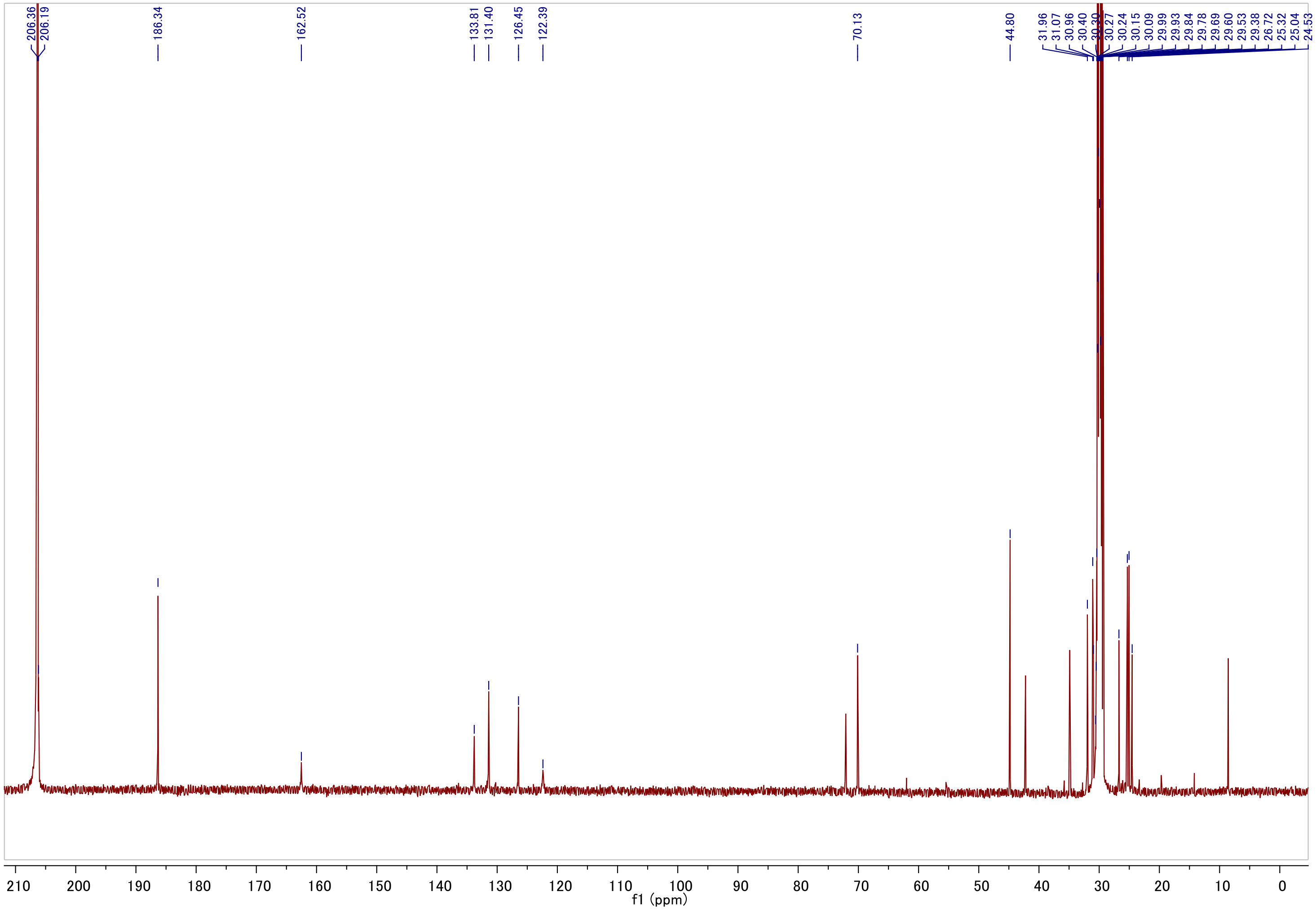

Figure S38. COSY spectrum of **4** (500 MHz, CD_3COCD_3)

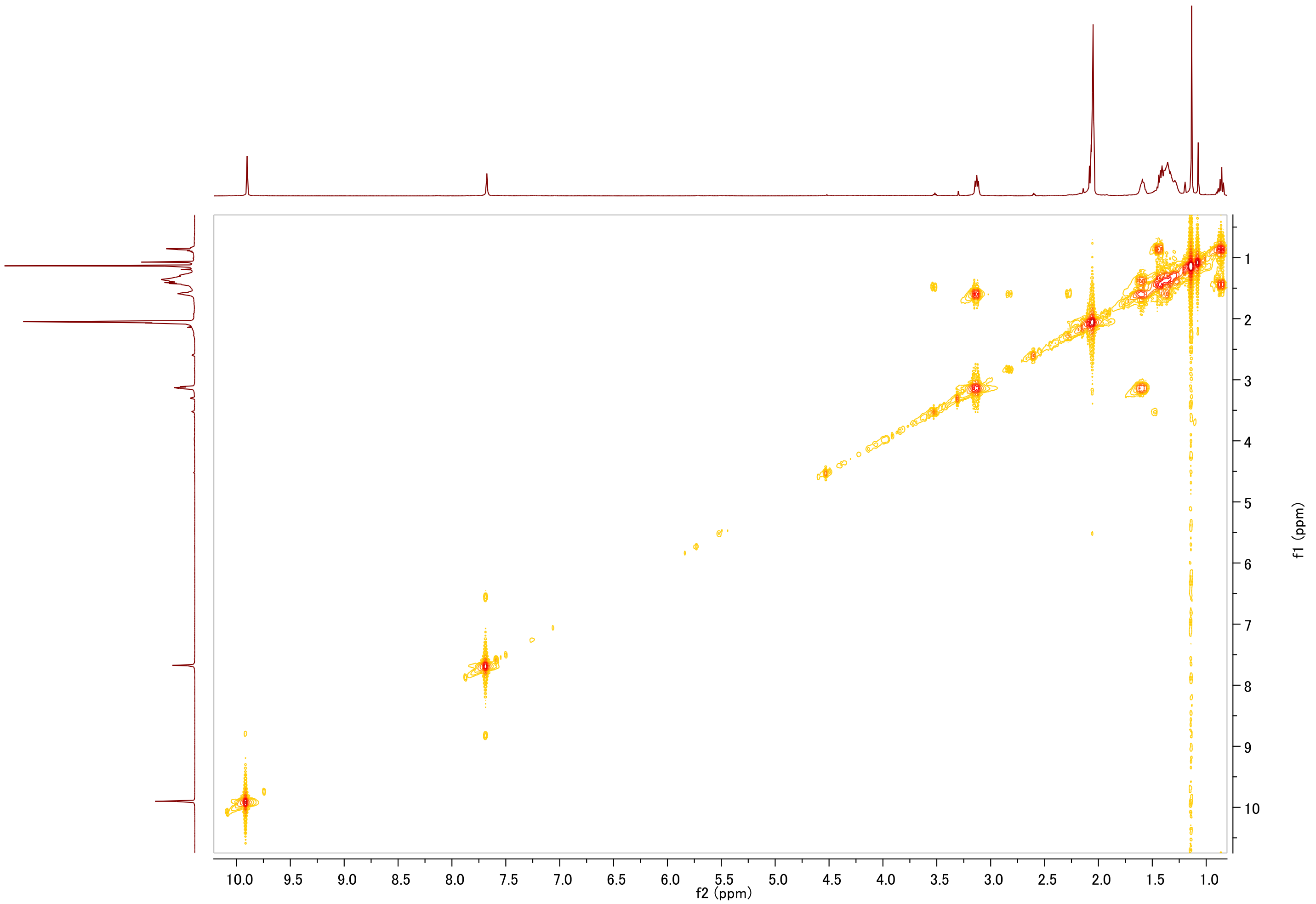

Figure S39. HSQC spectrum of **4** (500 MHz, CD_3COCD_3)

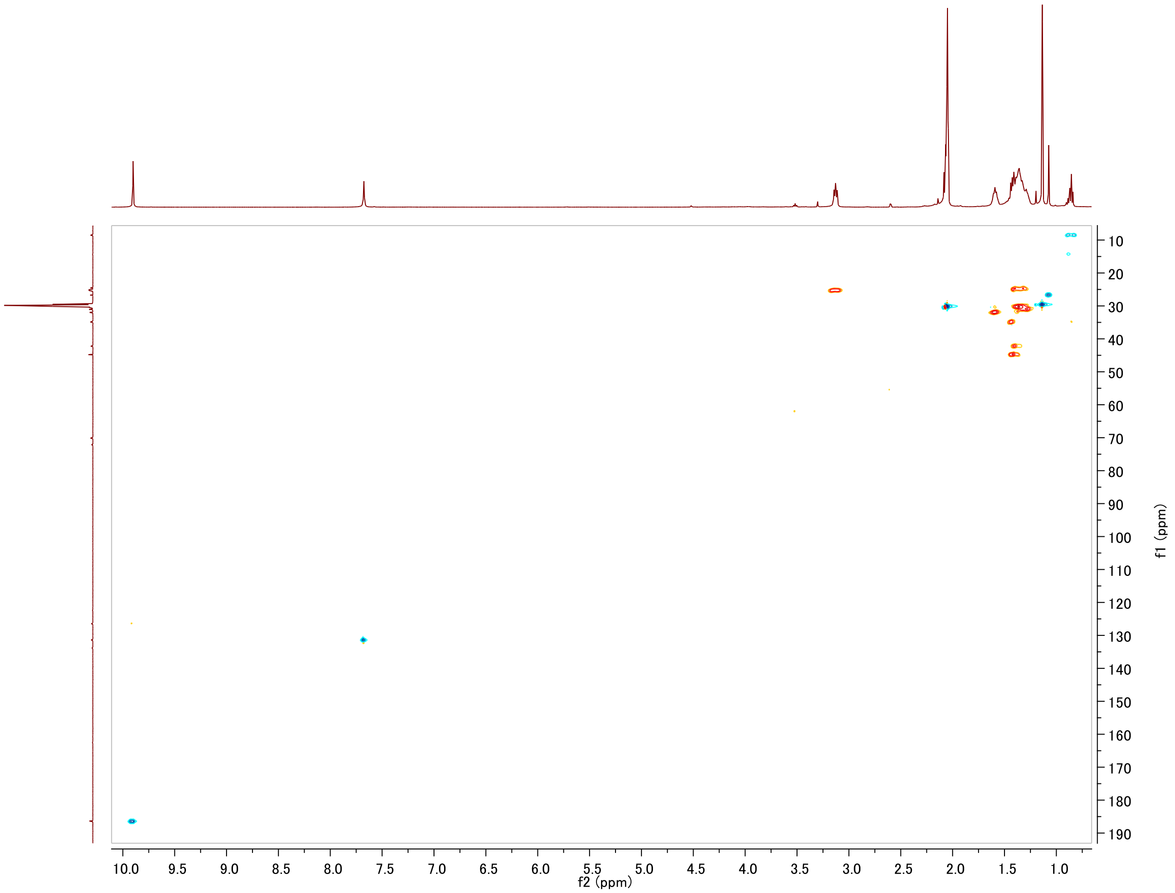

Figure S40. HMBC spectrum of **4** (500 MHz, CD_3COCD_3)

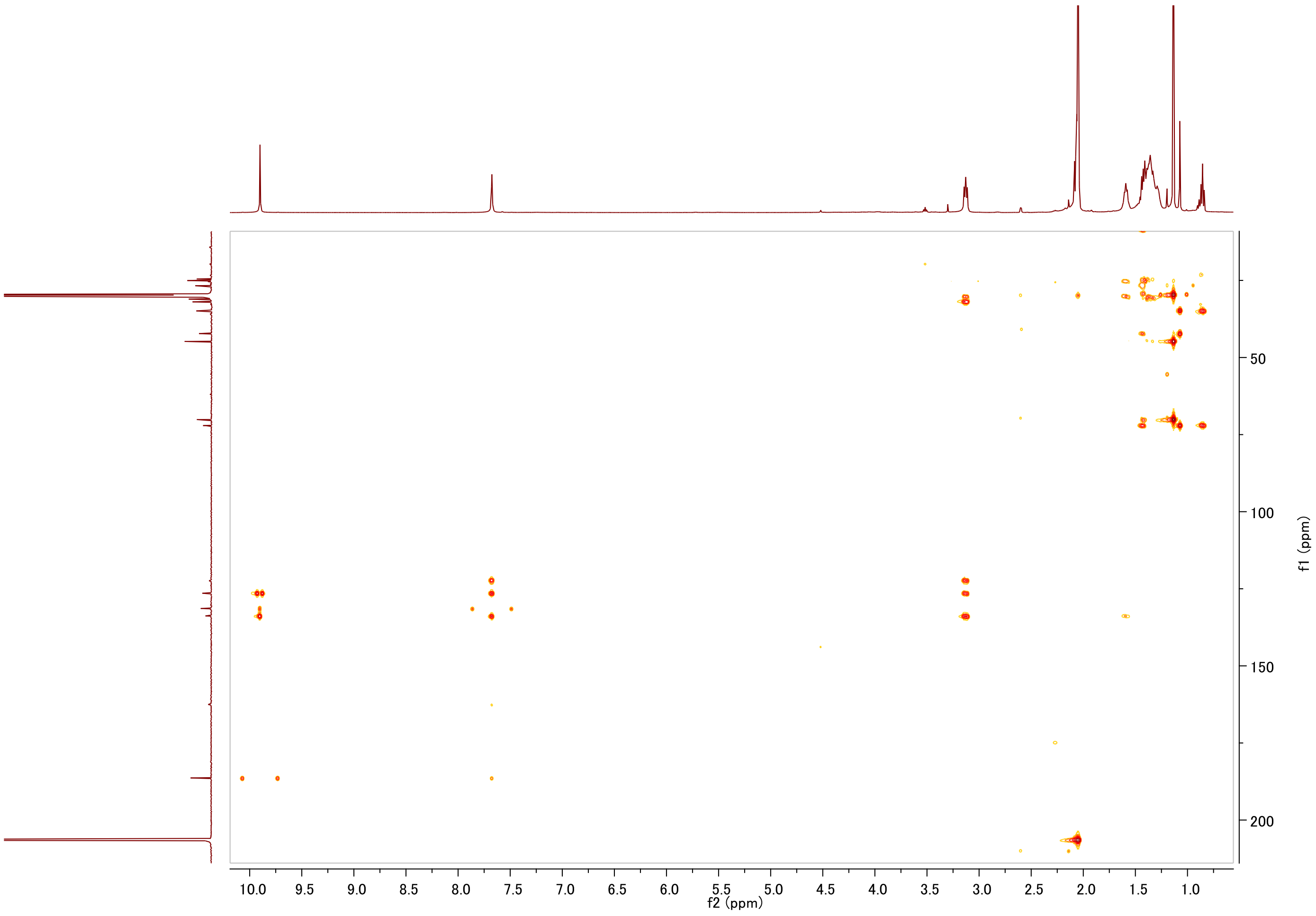

Figure S41. Negative HRESITOF mass spectrum of **4**

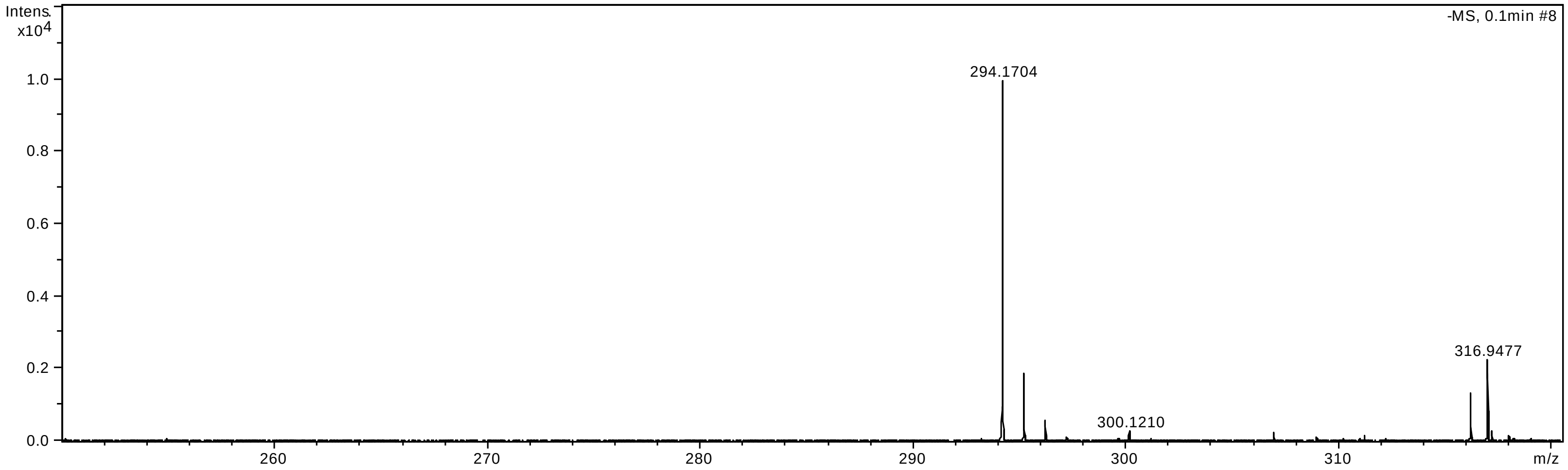

Figure S42. IR spectrum of **4**

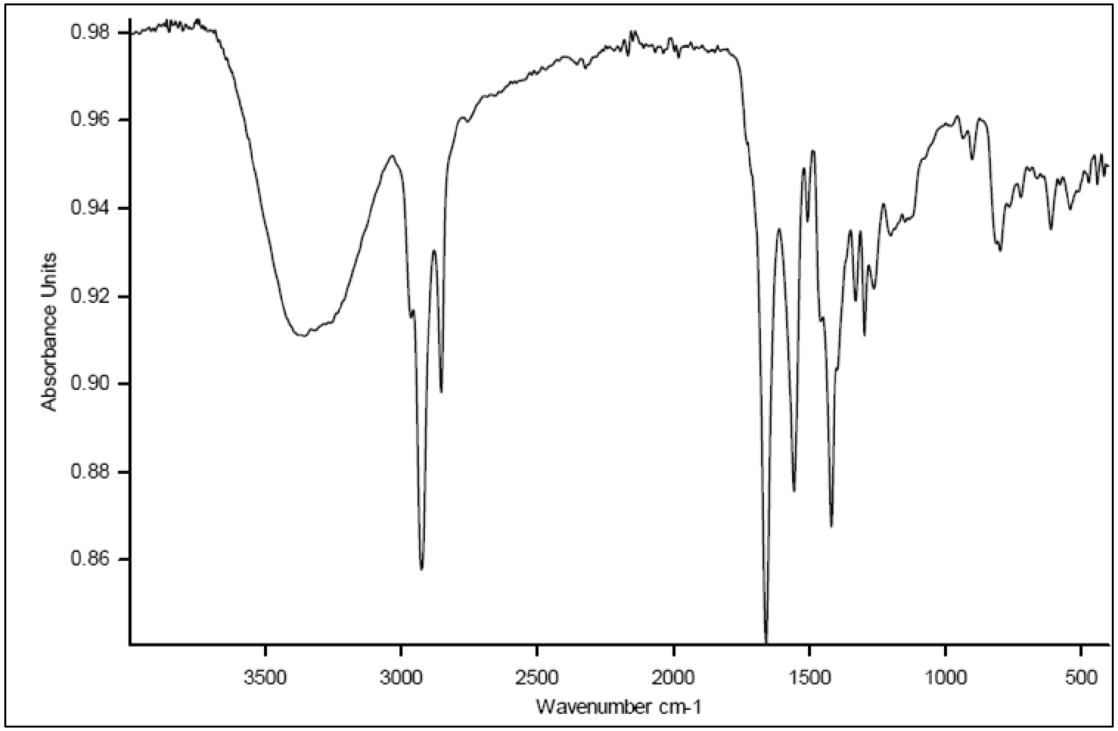

Figure S43. UV spectrum of **4**

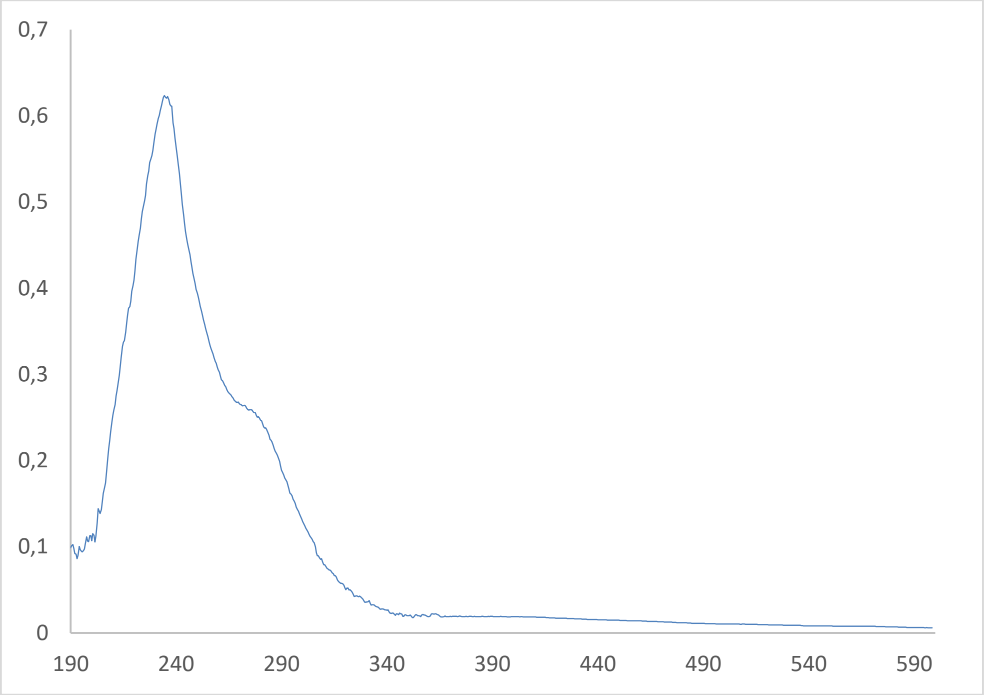

Figure S44. ^{1}H NMR spectrum of **5** (500 MHz, CD_3OD).

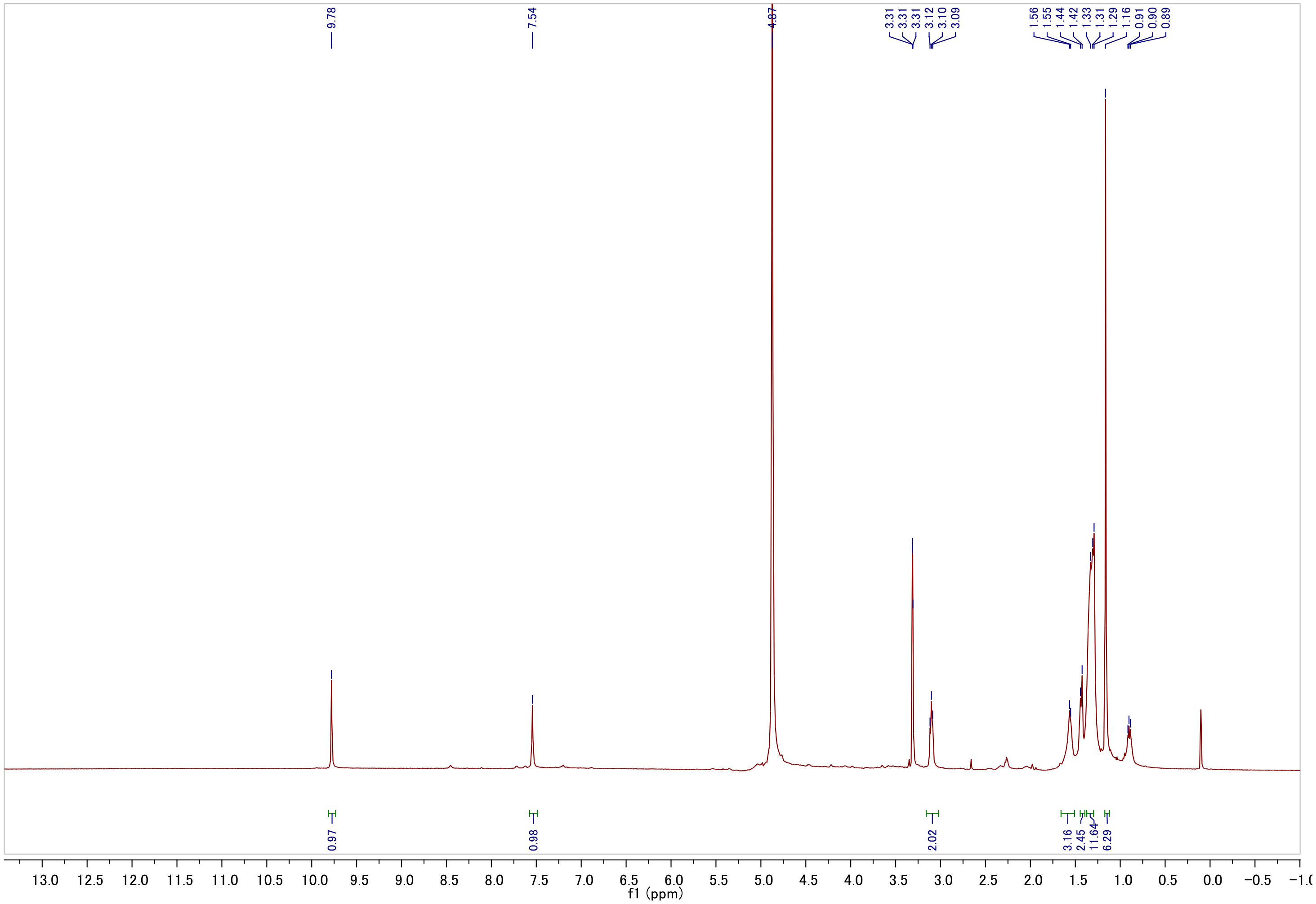

Figure S45. ^{13}C NMR spectrum of **5** (125 MHz, CD_3OD).

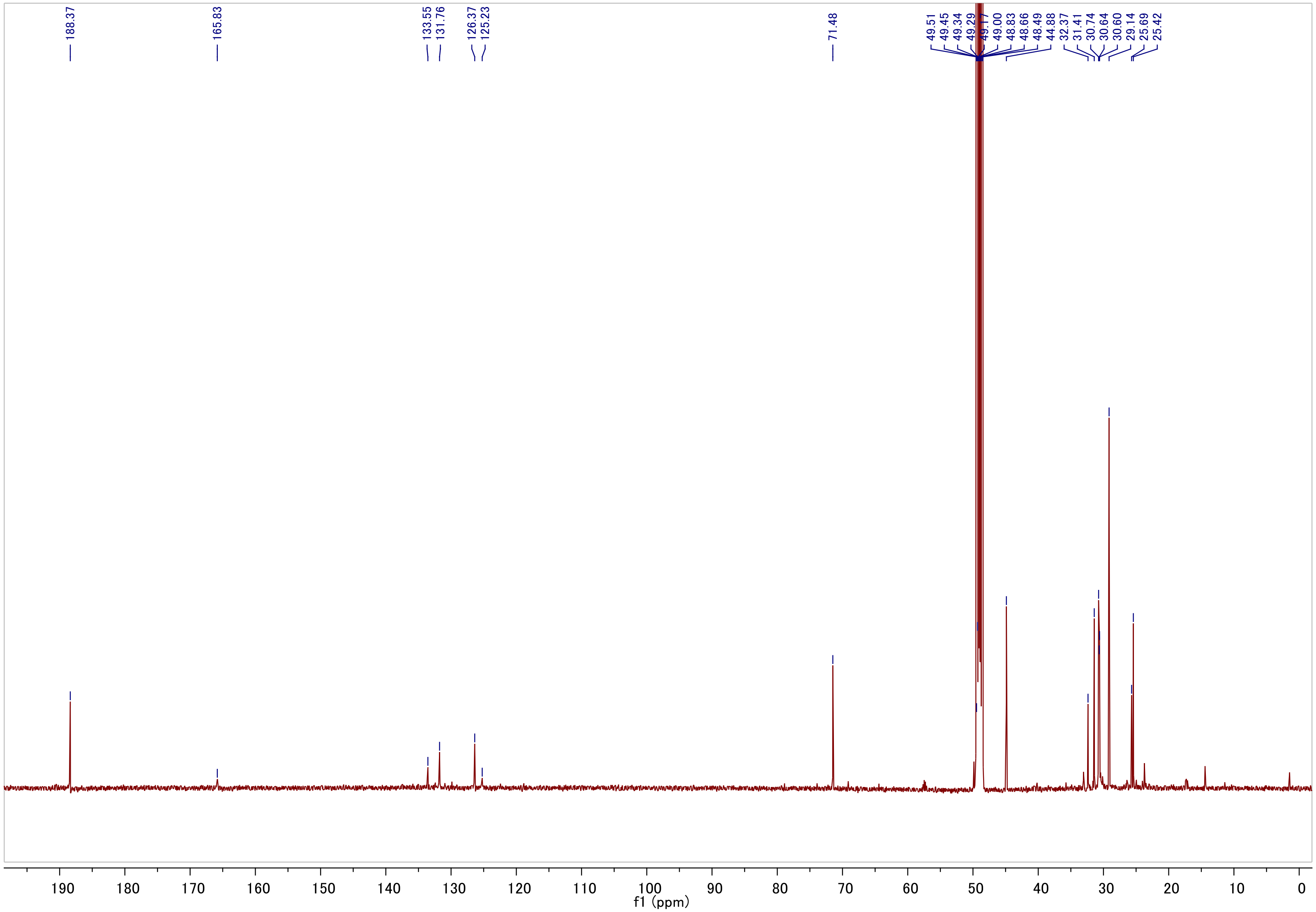

Figure S46. COSY spectrum of **5** (500 MHz, CD_3OD).

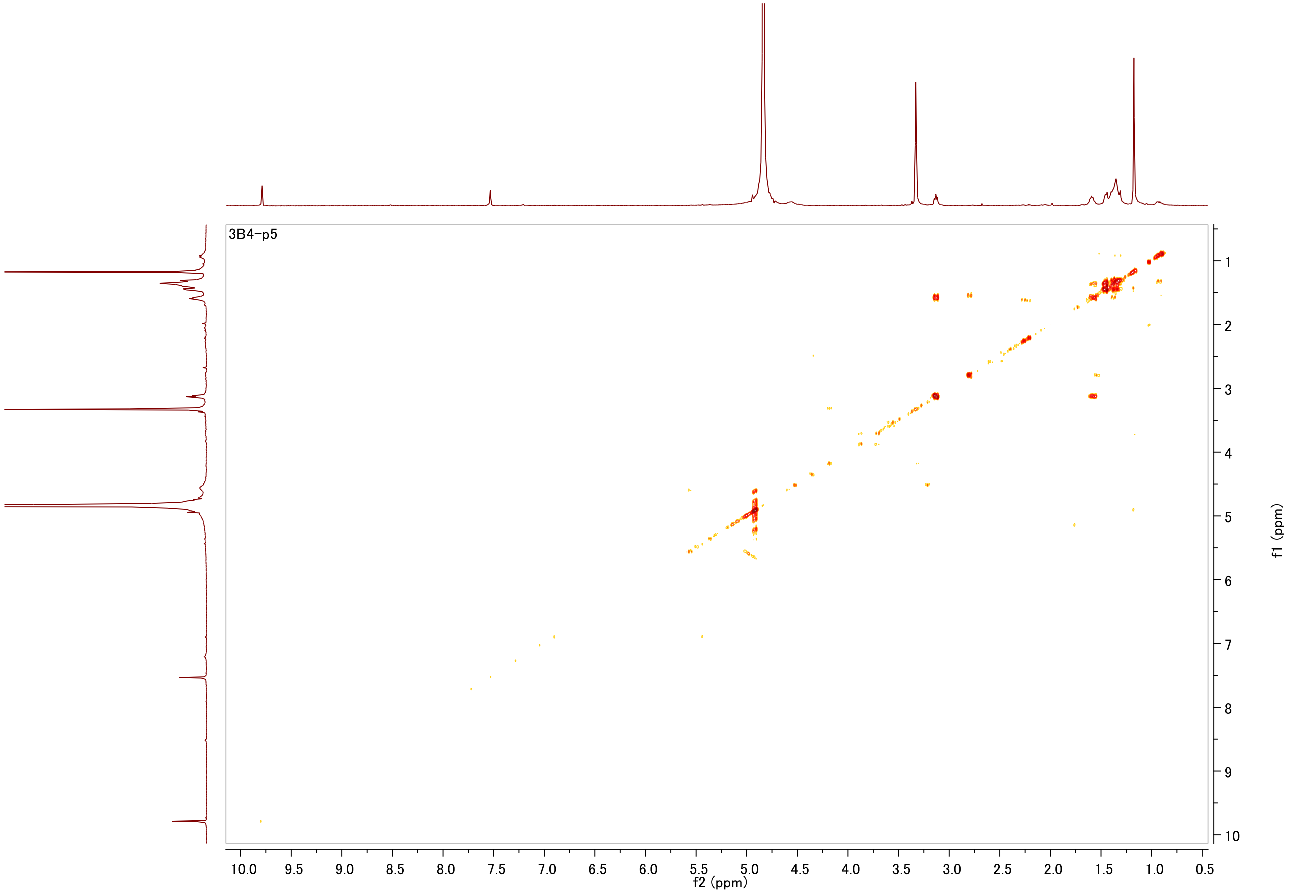

Figure S47. HSQC spectrum of **5** (500 MHz, CD_3OD).

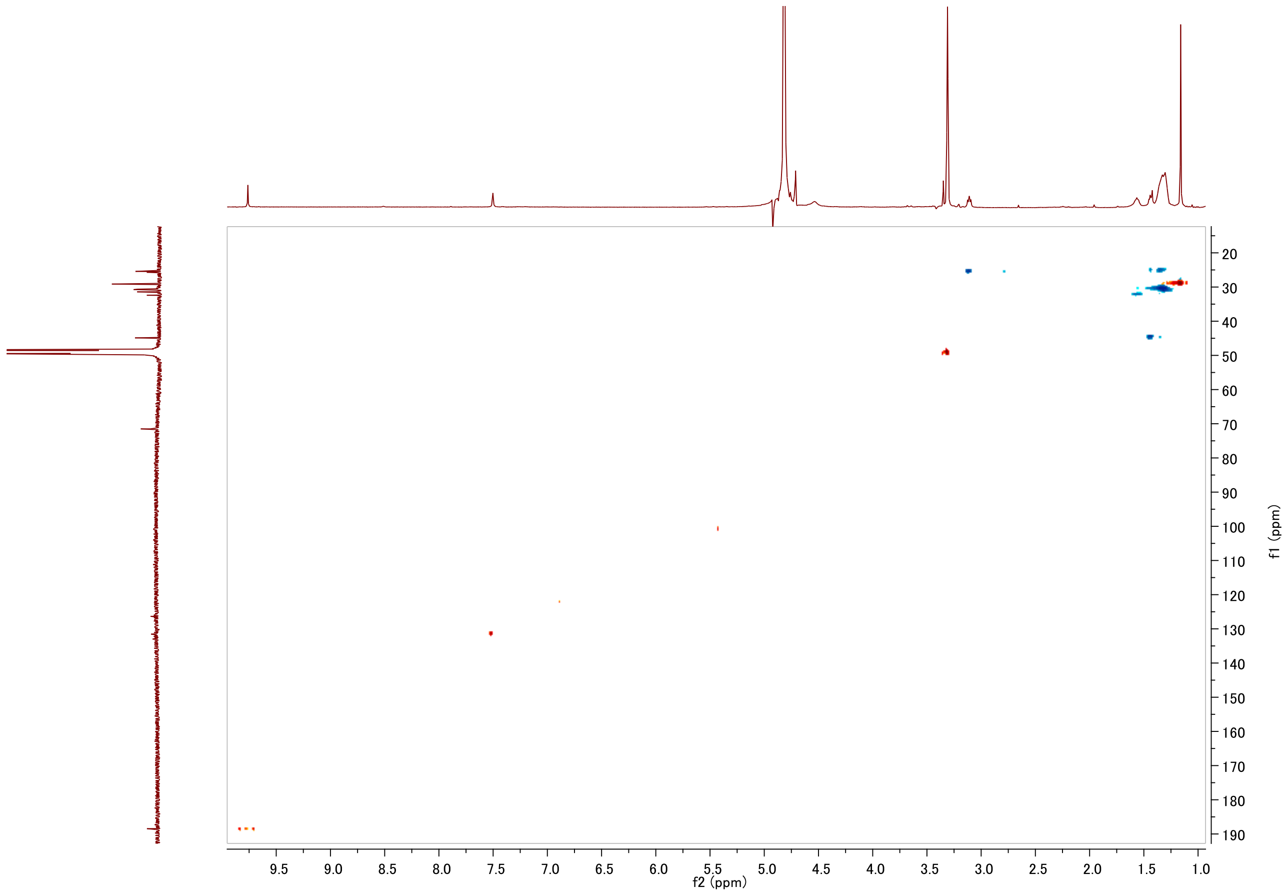

Figure S48. HMBC spectrum of **5** (500 MHz, CD_3OD).

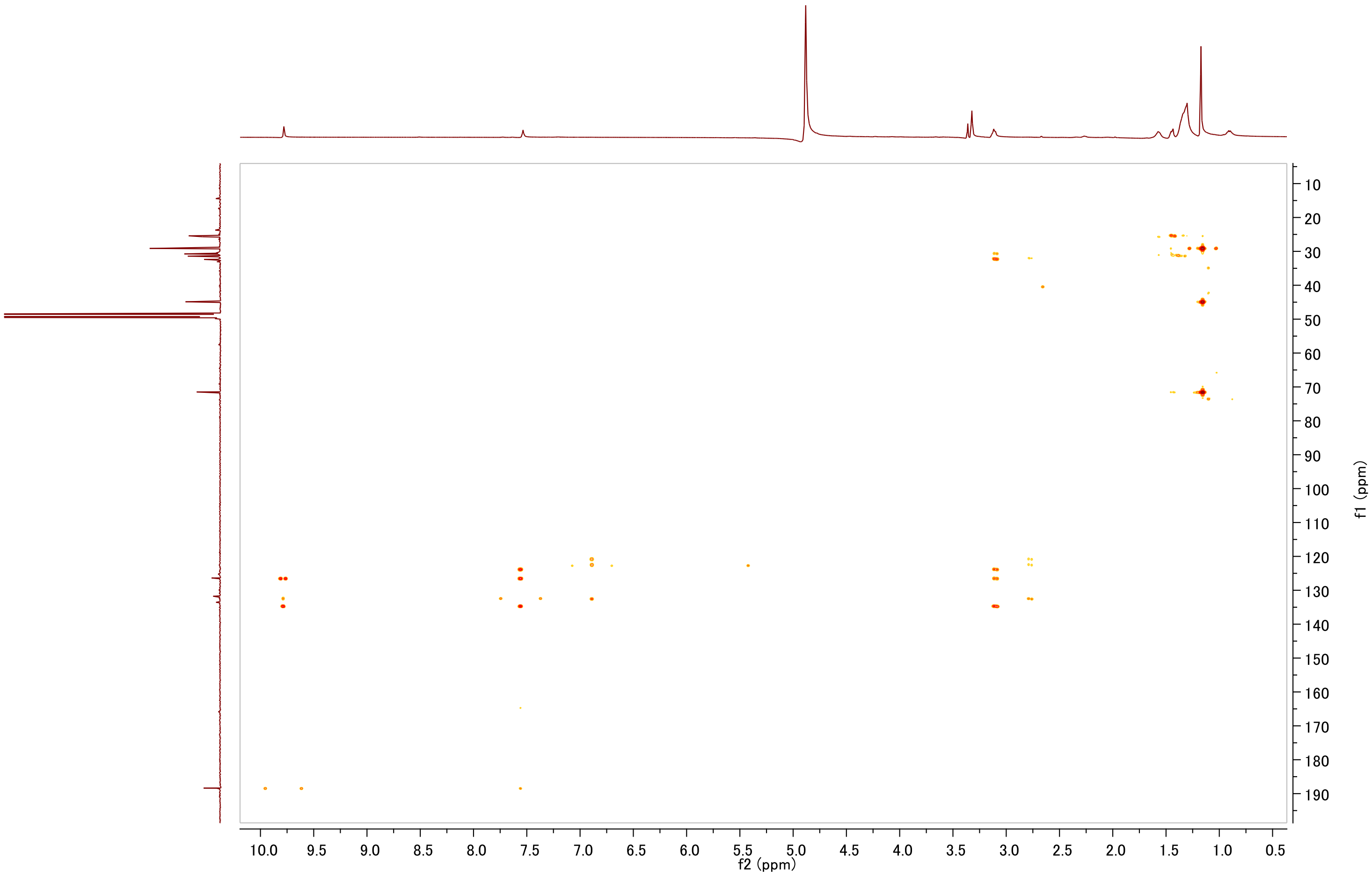

Figure S49. ^{15}N-HMBC spectrum of **5** (500 MHz, CD_3OD).

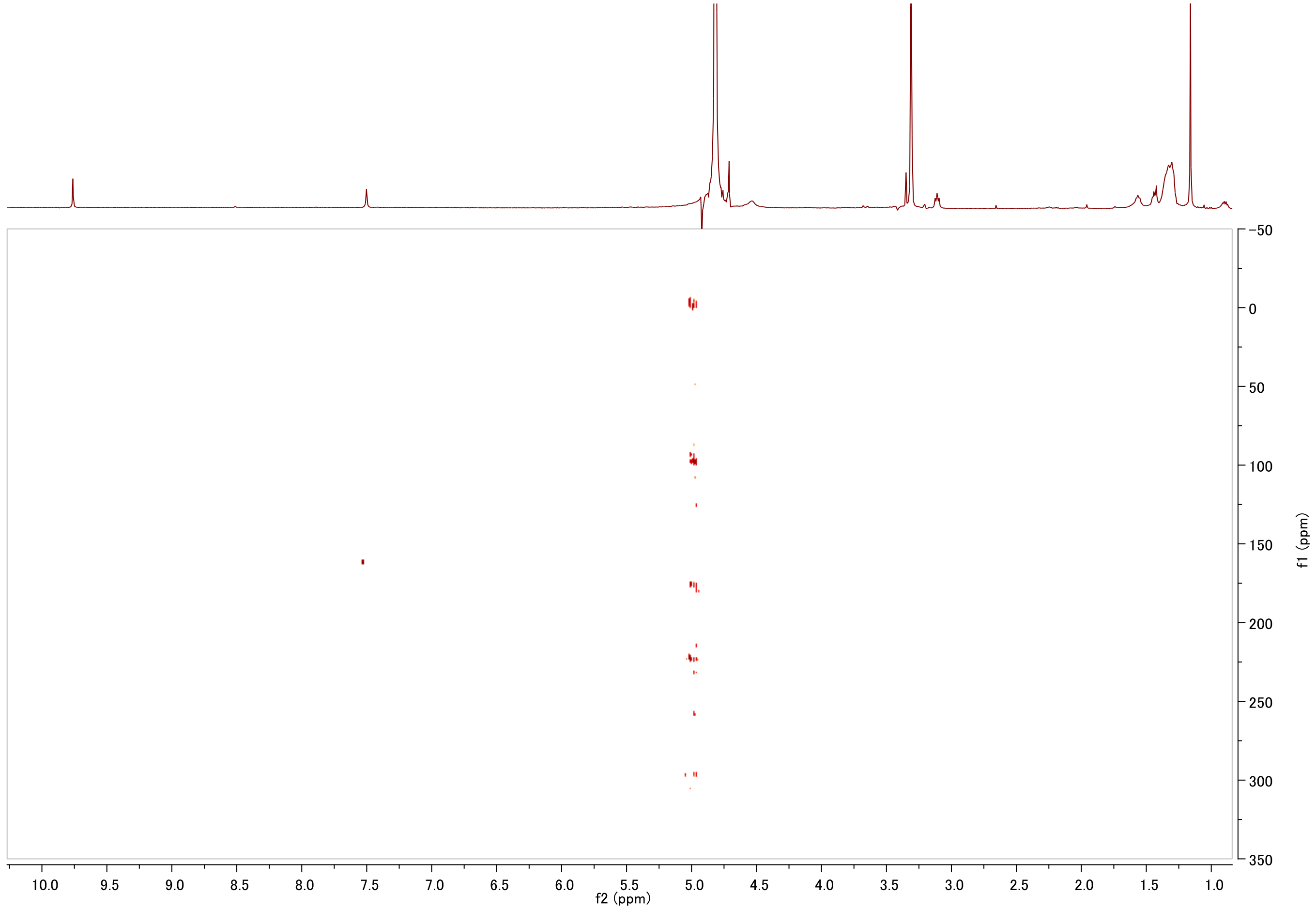

Figure S50. Positive HRESITOF mass spectrum of **5**

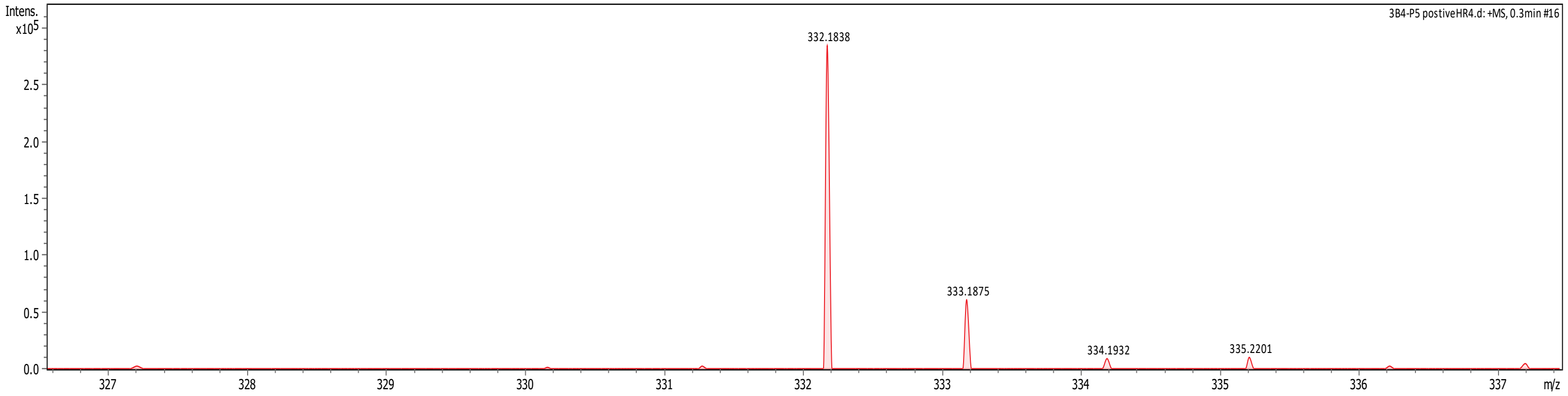

Figure S51. Negative ESITOF mass spectrum of **5**

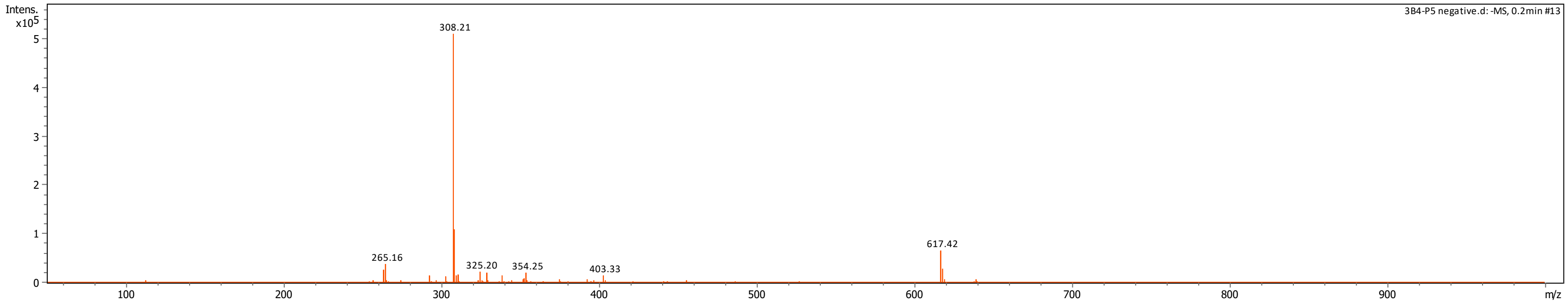

Figure S52. IR spectrum of **5**

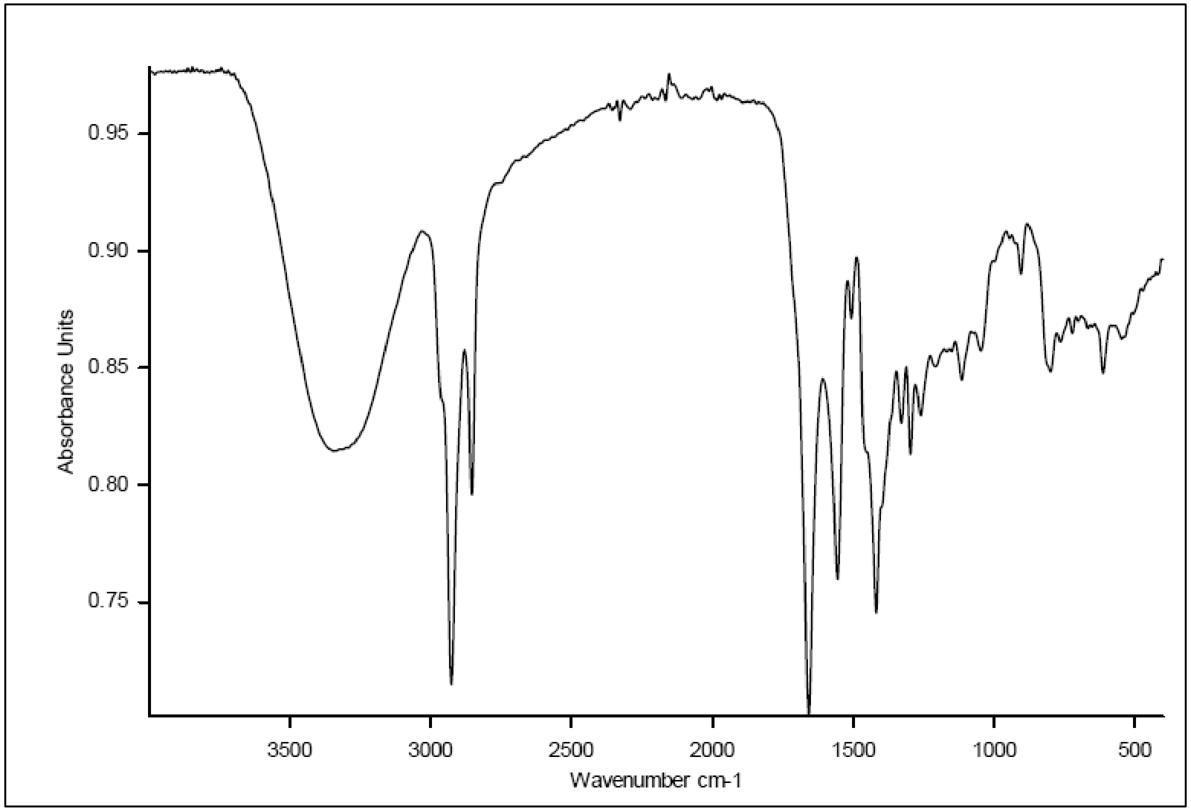

Figure S53. UV spectrum of **5**

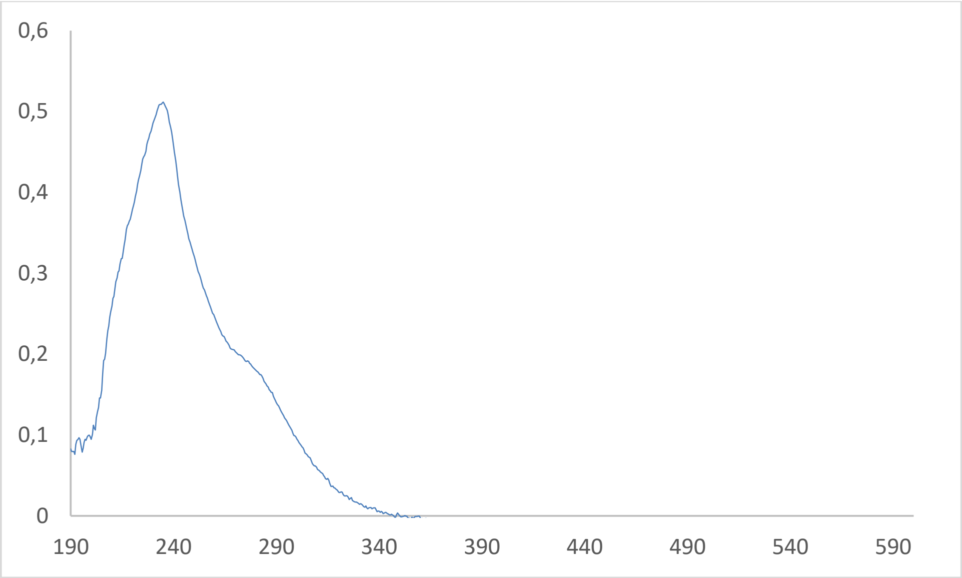

Figure S54. Representatives for natural products incorporating a β-alkylpyrrole scaffold

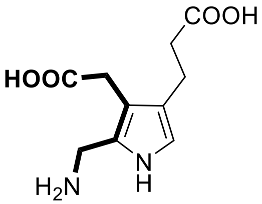

porphobilinogen

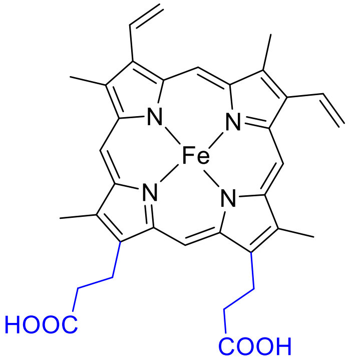

heme B

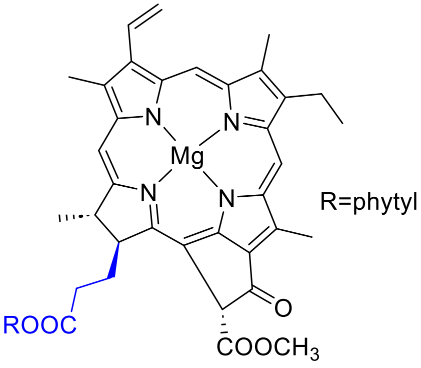

chlorophyll a

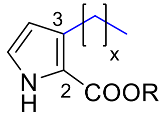

x=18 to 22
R=CHO, $COOCH_3$

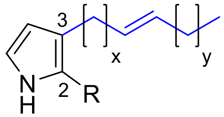

x+y=18, 20 R=COOH, $COOCH_3$
x=11, y=7 R=CHO

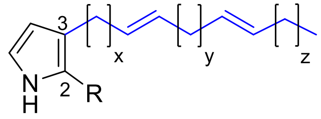

x+y+z=18 R=$COOCH_3$
x=6, y=7, z=5 R=CHO

reported structures for alkylpyrroles from the sponge *Oscarella lobularis*

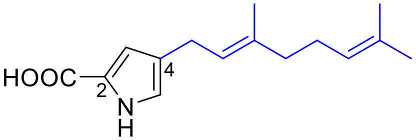

pyrrolostatin
Streptomyces chrestomyceticus EC40

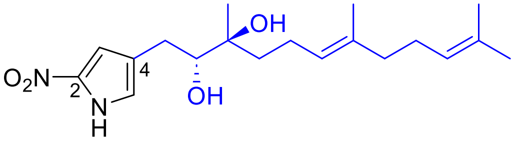

nitropyrrolin A
Streptomyces CNQ-509

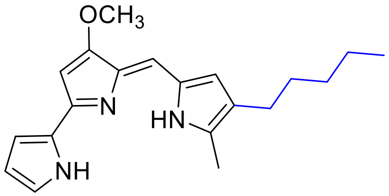

prodigiosin
Serratia strains

Figure S55. Representatives for secondary metabolites incorporating a pyrrole-2-carboxylic acid scaffold

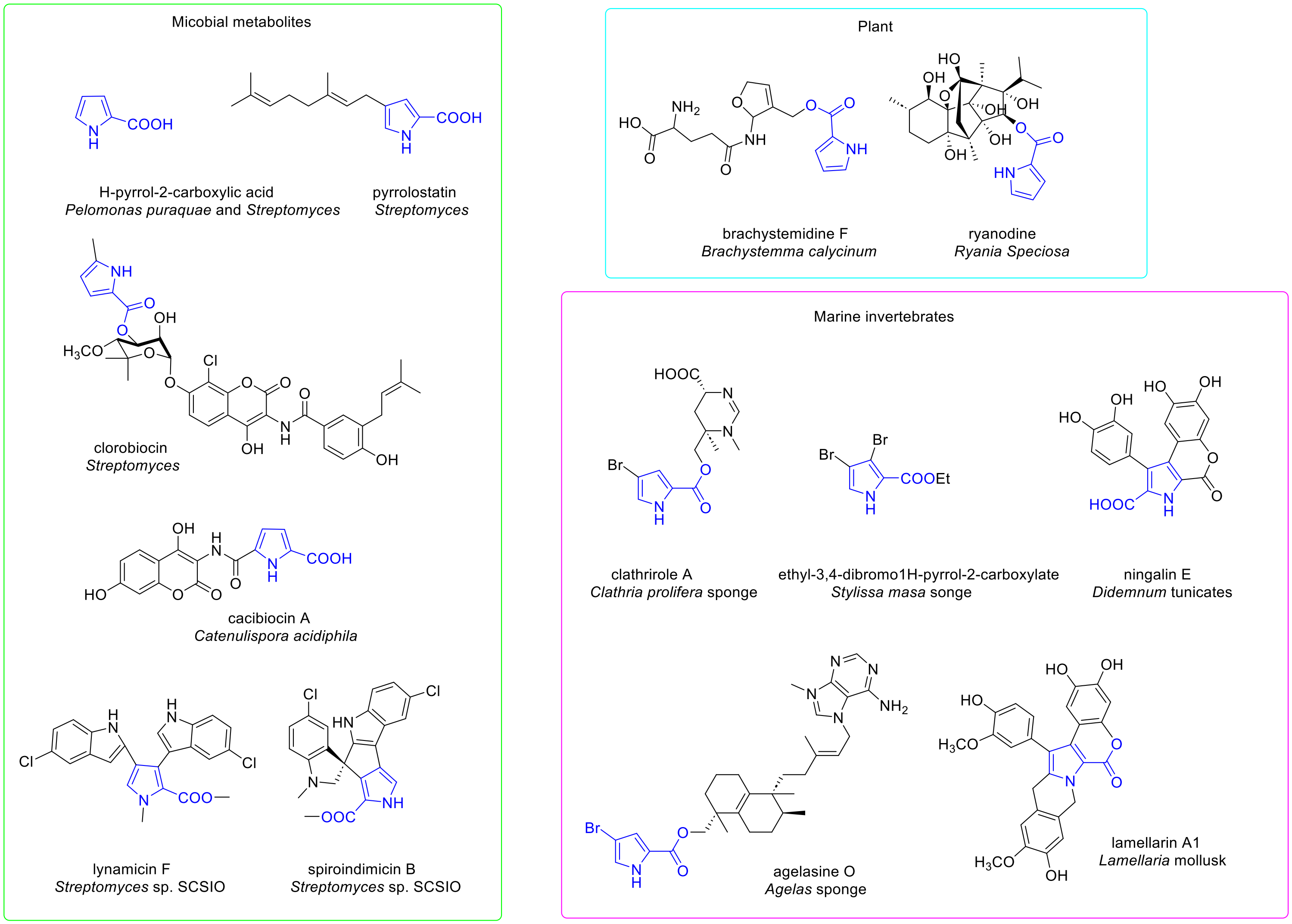

Table S1. HMBC (^{1}H to ^{13}C) for compounds (**1**–**5** and **1a**) at 500 MHz

**Pos.**	**1**	**1***	**1a**	**2**	**3**	**4**	**5***
5	2, 3, 4, 7	2, 3, 4, 7	3, 4, 6, 7, <u>C</u>H_3-N	2, 3, 4, 7	–	2, 3, 4, 6, 7	2, 3, 4, 6, 7
7	3, 4, 5	3, 4, 5	3, 4	3, 4, 5	3, 4, 5	3, 4, 5	3, 4, 5
8	2, 3, 4, 9, 10	3, 4, 9, 10	2, 3, 4, 9, 10	2, 3, 4, 9, 10	3, 9	2, 3, 4, 9, 10	2, 3, 4, 9, 10
9	3, 8	8, 10	3, 8, 11	3, 8, 11	–	3, 8, 10	3, 8
10	n.d.	n.d.	n.d.	n.d.	n.d.	n.d.	n.d.
11	n.d.	n.d.	n.d.	n.d.	n.d.	n.d.	n.d.
12	n.d.	n.d.	n.d.	n.d.	n.d.	n.d.	n.d.
13	12, 14, 15, 16	11, 12, 14, 15, 16	12, 14, 15, 16	n.d.	n.d.	n.d.	n.d.
14	–	–	–	n.d.	12	13, 15, 16, 17	–
15	12, 13, 14, 16	13, 14, 16	12, 13, 14, 16	13, 14	13, 14, 16	–	13, 14, 16, 17, 18
16	12, 13, 14, 15	13, 14, 15	12, 13, 14, 15	14, 15	14, 15	13, 14, 15, 17	–
17	–	–	–	–	–	13, 14, 15, 16	14, 15, 16, 18
18	–	–	–	–	–	–	14, 15, 16, 17
C*H*$_3$-N	–	–	2, 5	–	–	–	–
C*H*$_3$	–	–	6	–	–	–	–

Recorded in CD_3COCD_3.

* Recorded in CD_3OD.

n.d. Not determined.

Experimental

General experimental procedures

Optical rotation was measured using a JASCO P-1030 polarimeter. IR spectra were measured with a Bruker ALPHA II FTIR Spectrometer. UV spectra were recorded on a Shimadzu U-1601PC spectrophotometer. NMR experiments were performed on a Bruker AVANCE NEO 500 spectrometer. HRESITOFMS were recorded on a Bruker micrOTOF focus. Cosmosil 75C18-PREP (Nakalai Tesque, Inc., 75 μm) was used for octadecyldimethylsilyl (ODS) silica gel column chromatography. HPLC analysis and purification were performed on an Agilent HP1200 system equipped with a diode array detector.

Microorganism

Allostreptomyces sp. RD068384 was obtained from Biological Resource Center, National Institute of Technology and Evaluation, Chiba, Japan.

Fermentation

Strain RD068384 was cultured at 30 °C on a Bn-2 medium [soluble starch 0.5%, glucose 0.5%, meat extract 0.1% (Kyokuto Pharmaceutical Industrial Co., Ltd), yeast extract 0.1% (Difco Laboratories), NZ-case 0.2% (Wako Chemicals USA, Inc.), NaCl 0.2%, $CaCO_3$ 0.1%, and agar 1.5% in distilled water of pH 7.0]. The strain was inoculated into a 500 mL K-1 flask containing 100 mL of V-22 seed medium [soluble starch 1%, glucose 0.5%, NZ-case 0.3%, yeast extract 0.2%, Tryptone 0.5% (Difco Laboratories), K_2HPO_4 0.1%, $MgSO_4 \cdot 7H_2O$ 0.05%, and $CaCO_3$ 0.3% in distilled water of pH 7.0]. The flasks were shaken on a rotary shaker (200 rpm) at 30 °C for 6 days. Then, the seed culture (3 mL) was transferred into 500 mL K-1 flasks, each containing 100 mL of A-3M production medium [soluble starch 2.0%, glycerol 2.0%, glucose 0.5%, Pharmamedia 1.5% (Archer-Daniels-Midland Company, TX, USA), yeast extract 0.3%, and Diaion HP-20 1.0% (Mitsubishi Chemical, Tokyo, Japan) in distilled water, pH 7.0]. The inoculated flasks were placed on a rotary shaker (200 rpm) at 30 °C for 8 days.

Extraction and isolation

After fermentation, 100 mL of 1-BuOH were added to each flask, and the flasks were agitated on a rotary shaker for 1 h. The mixture was centrifuged at 6,000 rpm for 10 min, and the organic layer was separated from the aqueous layer containing the mycelium. Evaporation of the organic layer gave ca. 10.9 g of the extract from 4 L of culture. The extract was subjected to silica gel column chromatography with a step gradient of $CHCl_3$/MeOH (1:0, 20:1, 10:1, 4:1, 2:1, 1:1, and 0:1, v/v). Fraction 3 (10:1) was concentrated to give 395 mg of a brown solid, which was next fractionated by ODS column chromatography with a gradient of MeCN/0.1% HCO_2H solution (2:8, 3:7, 4:6, 5:5, 6:4, 7:3, and 8:2, v/v). The ODS fractions 3 (4:6) and 4 (5:5) were concentrated to afford 35 mg of semipure material, which was further purified by preparative HPLC (Cosmosil C18 AR-II, 10 × 250 mm, 4 mL/min, UV detection at 254 nm) with 33% MeCN/0.1% HCO_2H solution to yield **1** (1.2 mg, t_R 10.1 min), **2** (0.9 mg, t_R 12.1 min), **3** (0.8 mg, t_R 13.7 min), and **4** (2.3 g mg, t_R 17.3 min). After 22 min of isocratic elution, MeCN concentration was raised to 35% to yield **5** (1.7 mg, t_R 28.9 min). The above operation was repeated twice to increase the yields of compounds **1**–**5**, which overall yielded 6.5 mg of **1**, 3.1 mg of **2**, 2.6 mg of **3**, 7.2 mg of **4** and 5.6 mg of **5** from 12 L culture.

Cytotoxic activity

Kasumi-1 human acute myeloblastic leukemia cells were cultured in RPMI-1640 (Serana MCL-041, Germany), supplemented with 20% fetal bovine serum and 1% penicillin/streptomycin. In a 96 well plate 1×10^5 cells/mL (100 μL/well) were incubated at 37 °C for 24 h in an 100% humidity 5% CO_2 atmosphere to develop a complete monolayer sheet. The growth medium was decanted after confluent sheet of cells were formed; the cell monolayer was washed twice with media. Cisplatin and test compounds were dissolved in DMSO at 10 mM concentration, then 10 μL of this stock solution were added to 90 μL RPMI medium with 2% serum (maintenance medium). Half-fold serial dilutions of the tested sample were made in the maintenance medium. Each dilution (100 μL) was tested in different wells leaving 3 wells as control, receiving only maintenance medium. The cells were incubated at 37 °C and examined for any physical signs of toxicity including partial or

complete loss of the monolayer, rounding, shrinkage, or cell granulation. After, 20 μL of MTT solution (5mg/mL, Bio Basic Canada Inc.) were added to each well, shaken at 150 rpm for 5 min to thoroughly mix the MTT into the media. The cells were incubated (37 °C, 5% CO_2) for 4 h to allow the MTT to be metabolized. Media is decanted completely, then 200 μL DMSO were added and the plate was shaken at 150 rpm for 5 min to thoroughly dissolve the formazan (MTT metabolic product). The optical density was read at 560 nm, optical density should be directly correlated with cell quantity.

Tyrosinase inhibitory assay

Mushroom tyrosinase enzyme (Sigma Aldrich, St. Louis, USA), kojic acid (Pionner, Delhi, India) and L-tyrosine (Nice chemicals, Kerala, India) and phosphate buffer (50 mM, pH 6.8) were used in this assay. Kojic acid as well as the isolated compounds were dissolved in DMSO to prepare a 10 mM stock solution. Then, 100 μL of stock solution were added to 400 μL phosphate buffer (50 mM, pH 6.8) to get 2000 μM concentration. After, half-fold serial dilutions were prepared in phosphate buffer. The final DMSO concentration in the reaction mixture should not exceed 2%. The enzyme mushroom tyrosinase buffer (0.5 mg/mL) and the substrate L-tyrosine (2 mM) were dissolved in phosphate

Briefly, 25 μL sample (2000 μM), 50 μL mushroom tyrosinase solution and 75 μL phosphate buffer were mixed and incubated for 10 min at 37 °C. After, 100 μL of L-tyrosine was added. Then, the reaction mixture was further incubated at 37 °C for 20 min. The amount of dopachrome formed is proportional to the color formation which was measured at 475 nm. Kojic acid was used as a positive control. The extract inhibitory potential towards tyrosinase enzyme was calculated as follows: % of inhibition = [(Abs control − Abs sample)/Abs control] × 100.
